# Supplementary material for: Selene-Ethylenelacticamides and N-Aryl-Propanamides as Broad-Spectrum Leishmanicidal Agents
Source: Pathogens. 2023 Jan 13;12(1):136. doi: 10.3390/pathogens12010136 (PMC9860784; doi:10.3390/pathogens12010136)
Supplement: Supplementary file 1 [file pathogens-12-00136-s001.zip › pathogens-2046240-supplementary.pdf]

pathogens

Article

Selene-Ethylenelacticamides and *N*-Aryl-Propanamides as Broad-Spectrum Leishmanicidal Agents

Natália Ferreira de Sousa [1], Helivaldo Diógenes da Silva Souza [2], Renata Priscila Barros de Menezes [1], Francinara da Silva Alves [2], Chonny Alexander Herrera Acevedo [1], Thaís Amanda de Lima Nunes [3], Zoe L. Sessions [4], Luciana Scotti [1], Eugene N. Muratov [4], Francisco Jaime Bezerra Mendonça-Junior [1], Klinger Antônio da Franca Rodrigues [3], Petrônio Filgueiras de Athayde Filho [2] and Marcus Tullius Scotti [1,*]

[1] Post-Graduate Program in Natural and Synthetic Bioactive Products, Federal University of Paraíba, João Pessoa 58051-900, PB, Brazil
[2] Post-Graduate Program in Chemistry, Federal University of Paraíba, João Pessoa 58051-900, PB, Brazil
[3] Infectious Diseases Laboratory, Federal University of Delta of Parnaíba, Av. São Sebastião, nº 2819-Nossa Sra. de Fátima, Parnaíba 64202-020, PI, Brazil
[4] Laboratory for Molecular Modeling, Division of Chemical Biology and Medicinal Chemistry, UNC Eshelman School of Pharmacy, University of North Carolina, Chapel Hill, NC 27599, USA
* Correspondence: mtscotti@ccae.ufpb.br; Tel.: +55-83-99869-0415

SUPPLEMENTARY MATERIAL

S1. SM Homology Models

Twelve homology models were generated, as crystallography was not available for all targets. The models generated are as follows: two models regarding *L. infantum* and its interactions with Pteridine Reductase 1 (LiPTR1) and UDP-glucose-pyrophosphorylase (LiUD-Pase); four models of *L. amazonensis* with, respectively, Trypanothione Reductase (LaTR), Dihydroorotate Dehydrogenase (LaDHODH), Arginase (LaARG) and Lanosterol (LaLNT); four models between *L. braziliensis* and each of the following enzymes: Glycerol-3-Phosphate Dehydrogenase (LbGPD), Pteridine Reductase 1 (LbPTR1), Trypanothione Reductase (LbTR) and UDP-Glucose-Pyrophosphorylase (LbUDPase); and finally, models analyzing *L. major* with Glycerol-3-Phosphate Dehydrogenase (LmGPD) and Trypanothione Reductase (LmTR).

The enzymes mentioned above were chosen because they participate in mechanisms related to the survival of the parasite. The enzyme Pteridine Reductase 1 (LbPTR1) is a key enzyme of the folate pathway in parasitic protozoa of the genera *Leishmania* and *Trypanosoma.* This enzyme is able to catalyze the NADPH-dependent reduction of conjugated (folate) and unconjugated (biopterin) pterins to their terahydro forms, from substrates in an oxidized or dihydro state [62]; The enzyme UDP-glucose-pyrophosphorylase (LiUD-Pase) is responsible for the synthesis of the sugar nucleotide UDP-glucose, being located in the cytosol and in the bloodstream glycosomes of procyclic trypanosomes. This enzyme is essential for the growth of trypanosomes, since both the glycosomal and cytosolic metabolic pathways involving UGP are functional [63]; Dihydroorotate Dehydrogenase (LaDHODH) is involved in the synthesis of pyrimidines, which are essential for the survival and cell proliferation of parasitic organisms [64]; The Trypanothione Reductase (LaTR) enzyme is considered one of the most important targets for the treatment of Leishmaniasis, since this enzyme has been shown to be essential for the survival of these parasites by protecting them from oxidative stress [65]; Arginase (LaARG) is a trimeric enzyme that contains manganese and is responsible for hydrolyzing the amino acid L-arginine into L-ornithine and urea. L-ornithine is used to synthesize polyamines and is essential for parasite growth and host infection [66-67]. Polyamines are also essential for the production of trypanothione, an important antioxidant agent in the control of reactive oxygen species (ROS) [68]. In fact, the expression and activity of arginase in *Leishmania* contribute to a greater infectivity of the parasite and play an important role in the pathogenicity of the infection. An increase in arginase activity decreases the availability of L-arginine for nitric oxide synthase (NOS), and reduces the formation of NO and uncouples NOS, reducing the defensive capacity of the host and increasing the infectivity of the parasite [69-71]; The enzyme Lanosterol (LaLNT) is necessary for the biosynthesis of sterols, which serve as essential components of plasma membranes (bulk sterols, cholesterol in humans, ergosterol in fungi) and also as precursors of regulatory molecules that modulate growth processes, division, differentiation and development (sparkling sterols). Rapid sterol production is most crucial for rapid cell multiplication [72]. The last mechanism studied refers to the enzyme glycerol-3-phosphate dehydrogenase (LmGPD) from *Leishmania* and Trypanosoma, which plays an important role in the glycolytic pathway and also indirectly participates in the pentose phosphate pathway, catalyzing the reversible oxidation (redox reaction) of L-glycerol-3-phosphate (L-G3P) to dihydroxyacetone phosphate (DHAP) using a nicotinamide adenine dinucleotide (NADH) as a cofactor. Pentose phosphate plays an important role in parasite defense against reactive oxygen species produced by mammalian hosts, which are neutralized by NADH-dependent reactions. In addition, the glycolytic pathway produces amino acids and sugars, which are a rich source of carbon and energy for the amastigote forms (parasite form) [73].

Ramachandram plots, which evaluate all possible combinations of the dihedral angles Ψ (psi) and ϕ (phi) for each of the amino acids of a protein, were used to analyze the homology models. The model is considered acceptable when more than 90% of the amino

acids present are in the regions considered allowed and/or favored in the graphs (corresponding to the colored regions) seen in Figures S1, S2, S3 and S4. Regions in white represent outliers and improbable conformations. The plots revealed that the main-chain conformations presented at least 90% of residues in the most favored regions in all enzymes studied herein. This was likely due to the high similarity between the model sequences and the high resolution of the models.

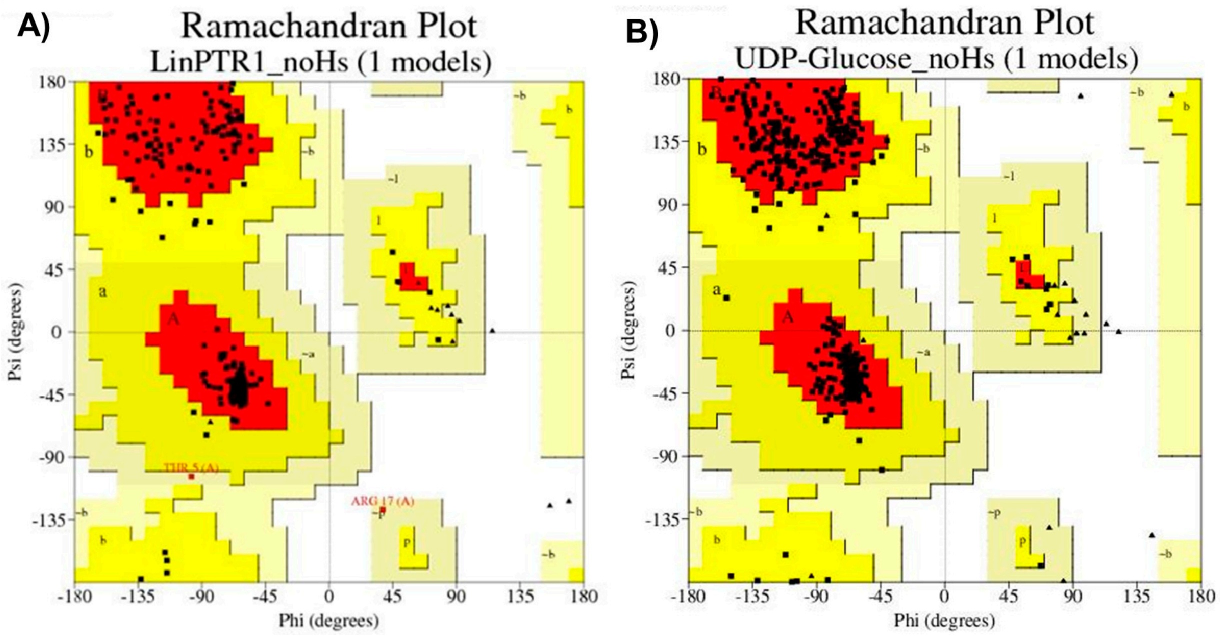

Figure S1. Ramachandran Graphic. Caption: I) *L. infantum* **A**) Pteridine Reductase, **B**) UDP Glucose Pyrophosphorylase;.

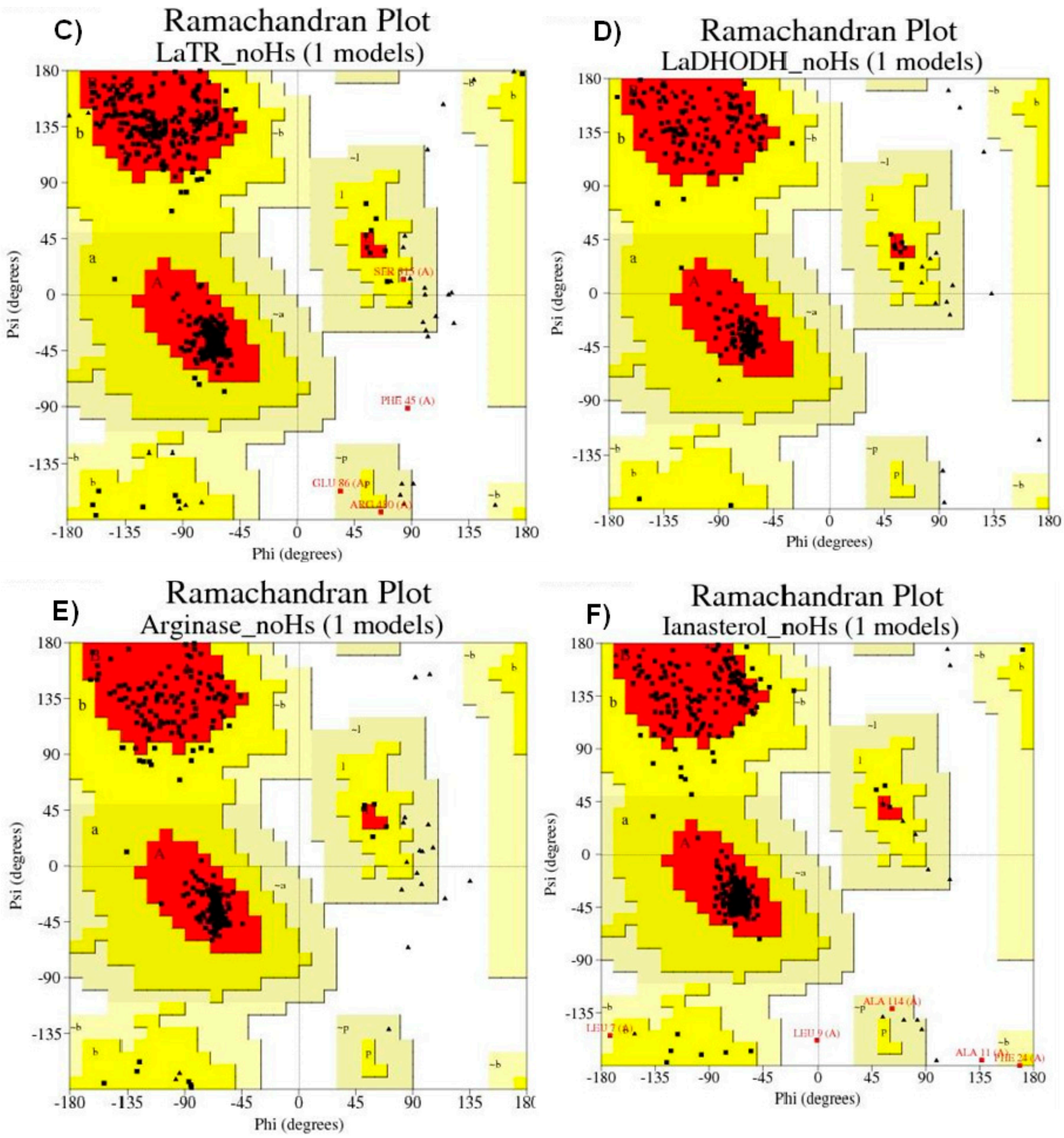

Figure S2. Ramachandran Graphic. Caption: II) *L. amazonensis* **C**) Trypanothione Reductase, **D**) Dihydrorotate Dehydrogenase, **E**) Arginase, **F**) Lanosterol.

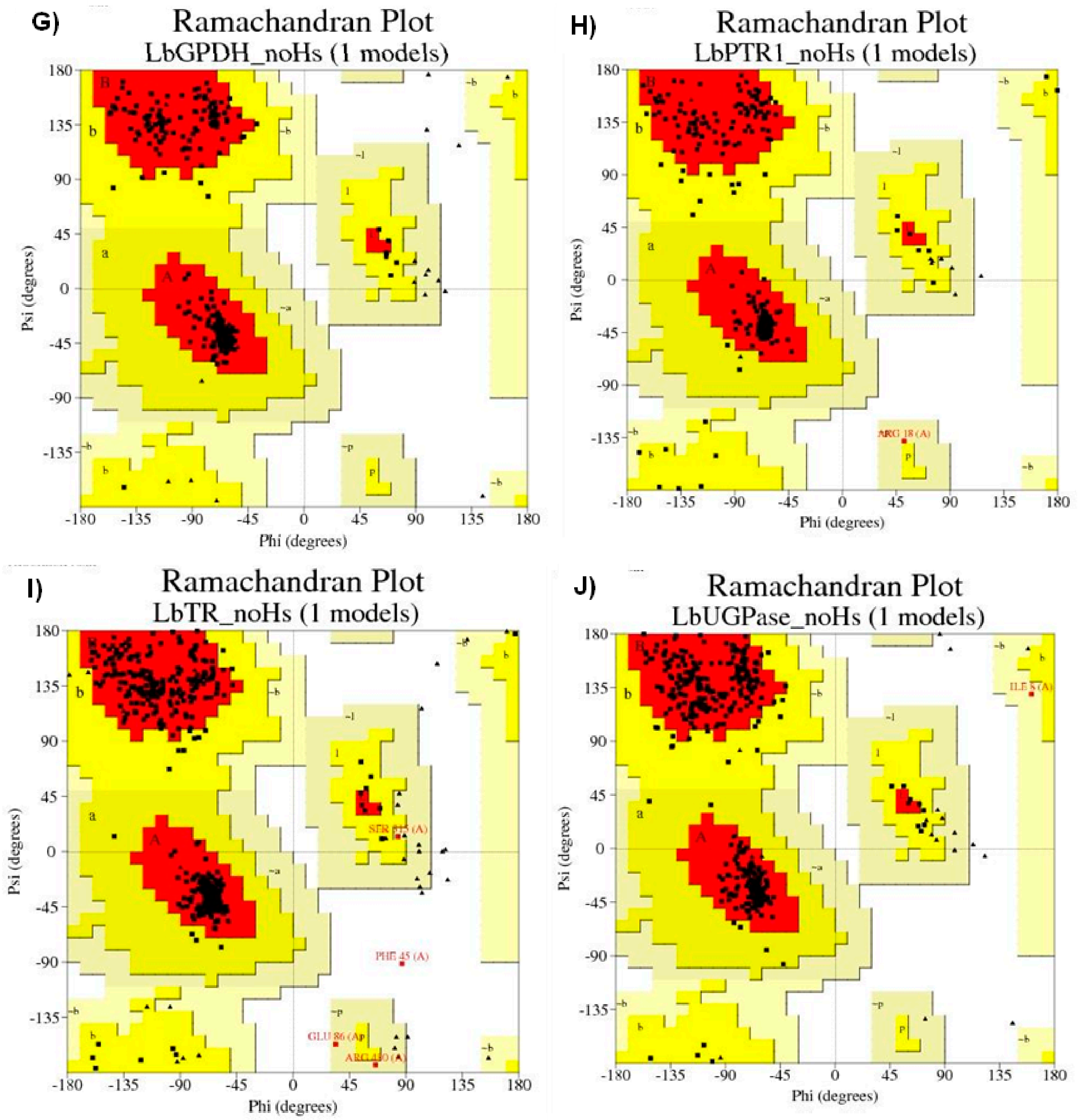

Figure S3. Ramachandran Graphic. Legend: III) *L. braziliensis* **G**) Glycerol-3-Phosphate Dehydrogenase, **H**) Pteridine Reductase 1, **I**) Trypanothione Reductase, **J**) UDP-Glucose-Pyrophosphorylase.

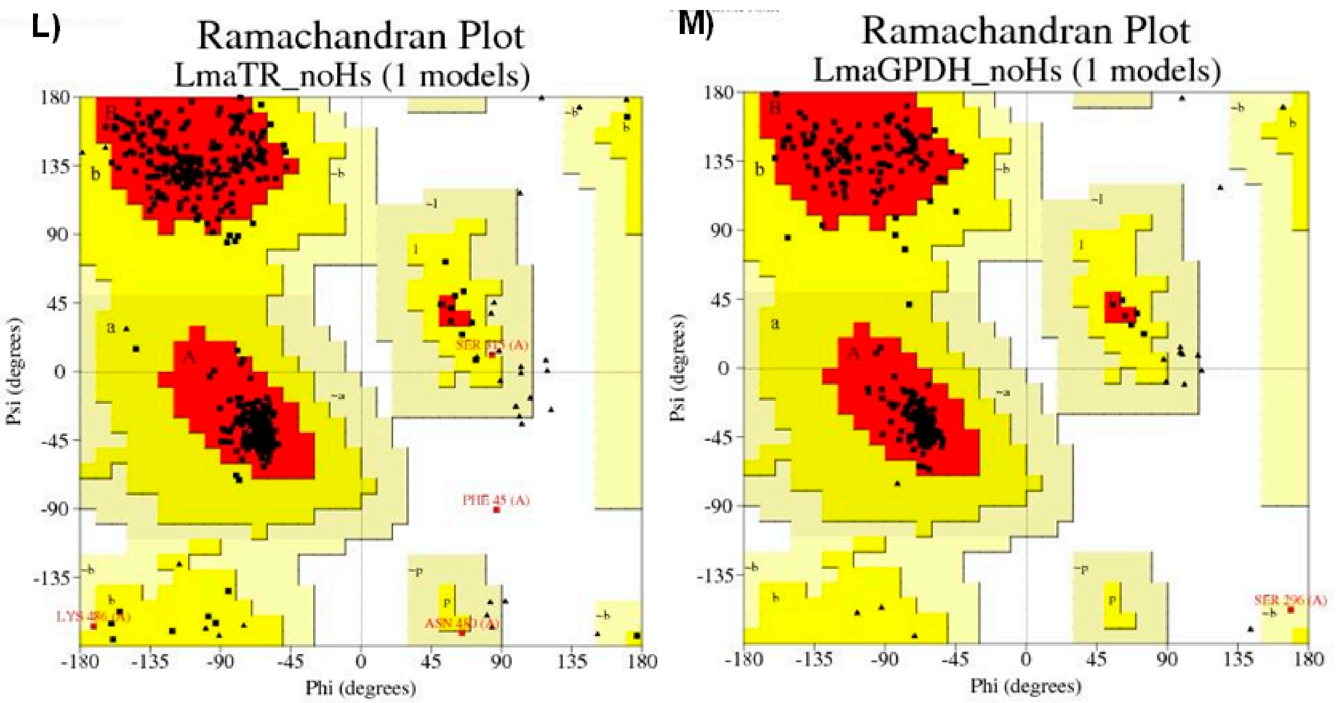

Figure S4. Ramachandran Graphic. Caption: IV) *L. major* **L**) Glycerol-3-Phosphate Dehydrogenase and **M**) Trypanothione Reductase.

Another parameter used was the WHAT IF Coarse Packing Quality Control module, which compares the distribution of the positions of the atoms around each residue. An average score that is less than –5.0 indicates bad or unusual atomic contacts. All homology models had mean score values above −5.0, with the mean score of all residues being respectively equivalent to: *L. infantum:* Pteridine Reductase (−0.962) and UDP Glucose Pyrophosphorylase (−0.620); *L. amazonensis:* arginase (−2.28), Lanosterol (−0.958), Dihydrorotate Dehydrogenase (−0.016), Trypanothione Reductase (−0.919); for *L. braziliensis:* Glycerol-3-Phosphate Dehydrogenase (−1.86), Trypanothione Reductase (−0.919), Pteridine Reductase (−0.952) and UDP-Glucose-Pyrophosphorylase (−0.609); *L. major:* Trypanothione Reductase (−0.894) and Glycerol-3-Phosphate Dehydrogenase (−1.87).

S2. SM. Molecular Docking

The selenoethylenelactamides that showed the highest combined probability (above 50% after calculation of combined probability between the two types of descriptors used, the Dragon 7 Descriptors and the VolSurf Descriptors) were analyzed through molecular docking simulations. The simulations were performed with 16 enzymes chosen to evaluate the potential mechanism of action for *L. infantum* through 14 alpha demethylase (CP51) (PDB: 3L4D) and Trypanothione reductase (PDB: 5EBK), for *L. major* through N-myristoyltransferase in complex with thienopyrimidine inhibitor IMP-0000081 (PDB: 6QDB), and using UDP-glucose pyrophosphorylase from *L. major* in complex with mur-rayamine-I (PDB: 5NZM). The rest of the proteins were obtained by homology, using the procedure described above.

Anchoring results were generated using the Moldock Score calculation algorithm and were validated by redocking the PDB ligand with enzymes for each species. More negative values indicated better predictions for the scoring function in consideration. As the MolDock Score performs the calculations, taking into account the molecular mass of the compound, standardization by Docking Score Correction (DSC) was performed in order to assess the influence of this parameter in the simulations under study. The calculation of the DSC is shown below:

$$\text{Docking Score Correction} = \frac{\text{Score}}{\text{Molecular Mass}}$$

In general, all the compounds interacted with all the enzymes tested, as negative energies were observed in all simulations performed. The molecular mass of the ligand for the proteins under study was directly linked to the final result, and the higher the molecular mass ratio of the endogenous or standard ligand, the lower the energy score of the tested compound. The energies obtained in the molecular docking simulations are described below.

Leishmania infantum

For *L. infantum,* the studied structures were subjected to molecular docking simulations using the CYP 51 (PDB: 3L4D) and Trypanothione reductase (PDB: 5EBK) proteins, as well as the enzymes obtained by homology, which correspond to Pteridine Reductase and UDP-Glucose pyrophosphorylase. the enzyme CYP-51 (PDB: 3L4D) catalyzes the removal of the 14α-methyl group from sterol precursors. The reaction is essential for membrane biogenesis and therefore has great potential to become a target for antileishmanial chemotherapy [74]; Trypanothione reductase (PDB: 5EBK), since trypanosomatid parasites have trypanothion as the main defense system against oxidative damage. Trypanothione, [N1,N8-bis(glutathionyl)spermidine] (TS2), synthesized by trypanothione synthase (TryS) and reduced to T(SH) 2 by trypanothione reductase (TR), is used by the double trypanethione/tryparedoxine peroxidase I (TXN/TXNPx) to neutralize hydrogen peroxide produced by macrophages during infection [75]; Pteridine reductase (PTR1) enzyme (obtained by homology), which represents a key enzyme of the folate pathway in protozoan parasites of the genera Leishmania and Trypanosoma and is a valuable drug target for tropical diseases. This enzyme is able to catalyze the NADPH-dependent reduction of conjugated (folate) and non-conjugated (biopterin) pterins to their tetrahydro forms, from oxidized substrates or dihydrostates [72] and UDP-Glucose Pyrophosphorylase which has been genetically validated as a target for antileishmanial drugs and represents the biosynthesis of uridine diphosphate-glucose (UDP-Glc) and its direct derivative UDP-galactose (UDP-Gal). The de novo biosynthesis of these two nucleotide sugars is controlled by the specific UDP-glucose pyrophosphorylase (UGP). Leishmania parasites also express a UDP-sugar pyrophosphorylase (USP) responsible for the recovery of monosaccharides that is capable of generating UDP-Gal and UDP-Glc. Inactivation of the parasite's two pyrophosphorylases, UGP and USP, results in parasite death [76]. Table S1 shows the MolDock Score scores for enzymes related to *L. infantum*. All tested compounds resulted in negative scores and therefore suggest interactions. Among others, NC70-133.28 Kcal/mol, NC62-136.46 Kcal/mol, NC76-126.16 Kcal/mol, NC73-128.52 Kcal/mol, NC69 -131.09 Kcal/mol and NC74-128.52 Kcal/mol consistently resulted in better binding scores than the crystallized ligand in the sterol 14 alpha-demethylase (CYP51) enzyme (PDB: 3L4D). NC74-147.19 Kcal/mol, NC76-151.16 Kcal/mol and NC60-152.19 Kcal/mol scored lower than the crystallized ligand for UDP-Glucose Pyrophosphorylase (homology derived). For the enzyme Trypanothione reductase, the compound NC74-128.52 Kcal/mol showed the highest affinity, while for the target Pteridia Reductase the lowest energy was obtained by the compound NC76-163.37 Kcal/mol, but it should be noted that NC74 and NC76 showed lower energies than the ligands under study in all studied structures. This indicates that these compounds may demonstrate multitarget potential and aid the modulatation of several relevant targets simultaneously. Theoretically, this could be very useful in increasing the therapeutic efficacy of the compound all while maintaining the limited safety concerns associated with using fewer compounds [77–79]. The residue interactions present between these compounds and the respective structures can be seen in Figure S5 I and II.

Table S1. The MolDock Score scores for enzymes related to *L. infantum.*

ID	PM	CYP-51 (PDB: 3L4D)		Trip. Reductase (PDB: 5EBK)		Pteridine Reductase		UDP-Glucose	
		MS	MS/PM	MS	MS/PM	MS	MS/PM	MS	MS/PM
NC04	208,04	–82,395	–0,39	–14,57	–0,07	–92,14	–0,44	–95,86	–0,46
NC05	208,04	–84,163	–0,40	–12,15	–0,05	–84,58	–0,40	–96,97	–0,46
NC06	208,04	–80,398	–0,38	–6,25	–0,03	–84,01	–0,40	–87,54	–0,42
NC19	262,53	–73,40	–0,27	–8,81	–0,03	–83,65	–0,31	–88,30	–0,33
NC31	347,04	–105,77	–0,30	–26,93	–0,07	–122,65	–0,35	–117,65	–0,33
NC32	361,05	–110,82	–0.30	–28,91	–0,08	–128,17	–0,35	–119,86	–0,33
NC33	362,05	–118,00	–0,32	–29,72	–0,08	–130,34	–0,36	–123,28	–0,34
NC34	363,04	–112,01	–0,30	–31,17	–0,08	–121,31	–0,33	–119,91	–0,33
NC35	363,04	–115,51	–0,31	–30,87	–0,08	–133,99	–0,36	–118,70	–0,32
NC36	366,99	–105,78	–0.28	–21,90	–0,05	–113,47	–0,30	–114,53	–0,31
NC37	372,03	–116,09	–0,31	–31,94	–0,08	–127,66	–0,34	–126,93	–0,34
NC39	372,03	–114,72	–0,30	–30,90	–0,08	–121,85	–0,32	–122,15	–0,32
NC40	375,07	–112,43	–0,29	–39,39	–0,10	–125,45	–0,33	–122,66	–0,32
NC41	378,01	–109,28	–0,28	–35,85	–0,09	–129,44	–0,34	–127,23	–0,33
NC42	383,01	–124,08	–0,32	–36,70	–0.09	–126,97	–0,33	–132,85	–0,34
NC43	383,01	–122,27	–0,31	–30,98	–0.08	–126,78	–0,33	–133,44	–0,34
NC44	383,01	–119,03	–0,31	–34,98	–0.09	–130,22	–0,33	–135,26	–0,35
NC46	392,02	–114,64	–0,29	–25,11	–0,06	–131,93	–0,33	–129,83	–0,33
NC47	392,02	–123,80	–0,31	–37,05	–0,09	–136,21	–0,34	–123,43	–0,31
NC48	392,02	–106,68	–0,27	–31,11	–0,07	–124,20	–0,31	–113,37	–0,28
NC51	404,32	–110,33	–0,27	–45,75	–0,11	–128,68	–0,31	–125,22	–0,30
NC53	410,94	–106,83	–0,25	–22,91	–0.05	–119,18	–0,29	–110,73	–0,26
NC55	422,99	–115,55	–0,27	–32,59	–0,07	–133,94	–0,31	–124,22	–0,29
NC56	422,99	–111,28	–0,26	–35,26	–0,08	–130,53	–0,30	–132,81	–0,31
NC57	422,99	–110,78	–0,26	–29,96	–0,07	–117,97	–0,27	–127,32	–0,30
NC58	424,95	–115,22	–0,27	–29,45	–0,06	–121,31	–0,28	–124,47	–0,29
NC60	433,05	–119,60	–0,27	–27,99	–0,06	–142,99	–0,33	–152,19	–0,35
NC61	434,36	–106,17	–0,24	–47,37	–0,10	–147,38	–0,33	–141,16	–0,32
NC62	446,07	–136,46	–0,30	–38,18	–0,08	–146,82	–0,39	–143,91	–0,32
NC64	469,00	–121,66	–0,25	–34,67	–0,07	–138,96	–0,29	–135,00	–0,28
NC66	478,41	–122,31	–0,25	–49,34	–0,10	–143,82	–0,30	–140,39	–0,29
NC69	513,10	–131,09	–0,25	–49,37	–0,09	–152,83	–0,29	–138,92	–0,27
NC70	516,38	–133,28	–0,25	–59,62	–0,11	–156,17	–0,30	–146,01	–0,28
NC73	530,41	–128,15	–0,24	–56,63	–0,10	–159,02	–0,29	–143,72	–0,27
NC74	531,07	–128,52	–0,24	–65,01	–0,12	–157,28	–0,29	–147,19	–0,27
NC76	543,14	–126,16	–0,23	–63,02	–0,11	–163,37	–0,30	–151,16	–0,27

Ligante	306,27	–116,85	–0,38	–45,11	–0,14	–141,03	–0,51	–146,61	–0,42

Legend: MS = MolDock Score [kj.mol–1]; MW = Molecular Weight; MS/PM = Relationship between interaction energy and molecular weight [kj.mol–1].

I)Compound NC74

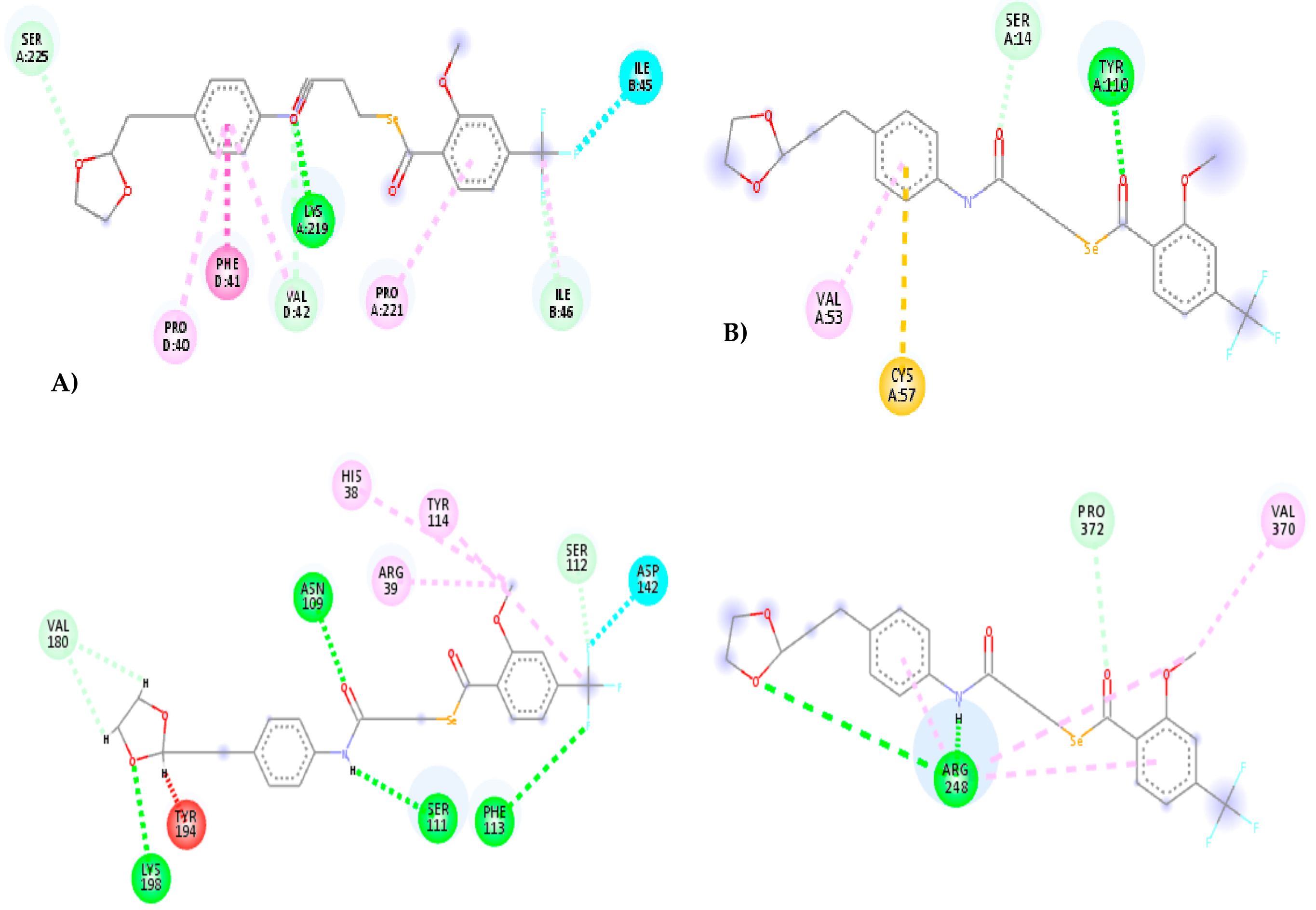

A) B) C) D)

II) Compound NC76

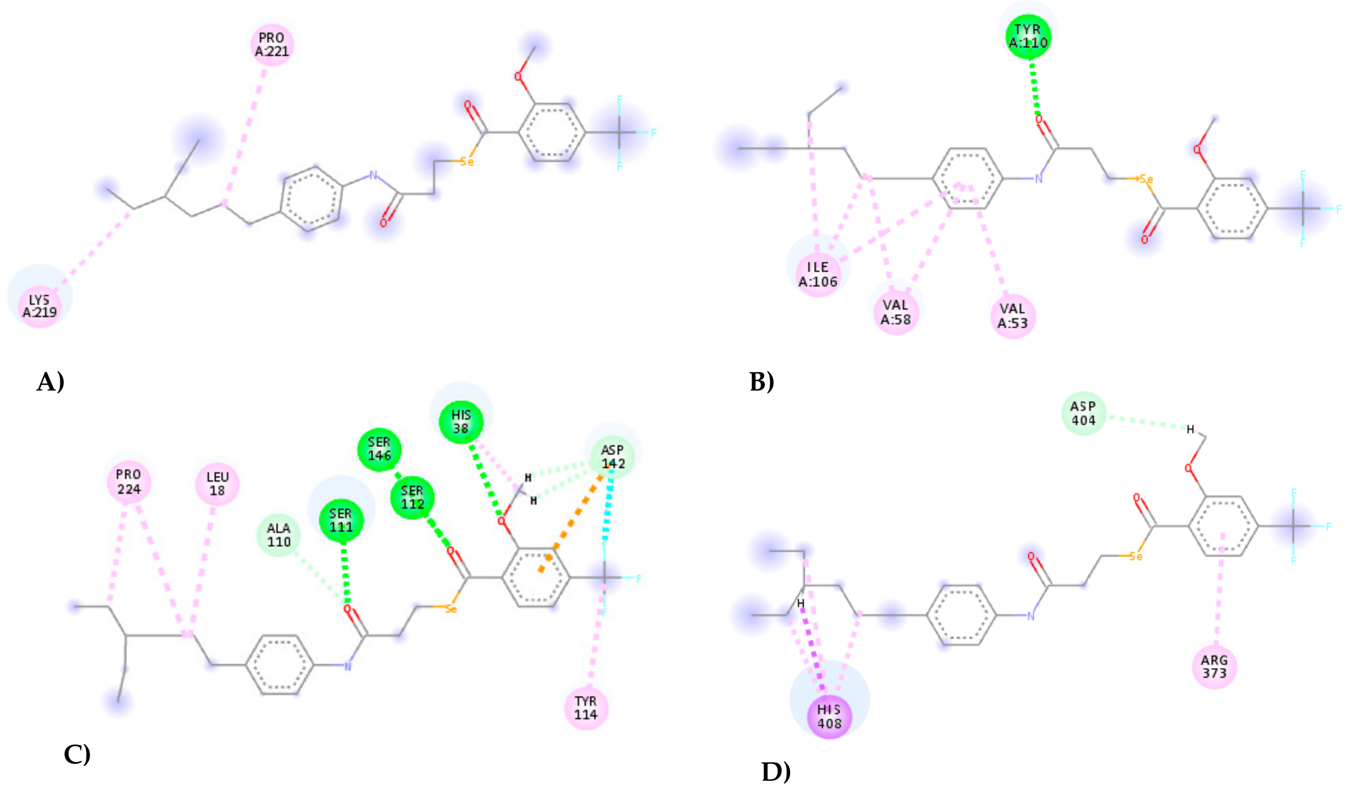

Figure S5. Interactions performed by compounds NC74 and NC76 with the corresponding amino acid residues. Legend: I) Compound NC74 and II) Compound NC76. **A**) CYP 51 (PDB: 3L4D), **B**) Trypanothione Reductase (PDB: 5EBK), **C**) Pteridine Reductase, **D**) UDP. **Colors:** Green and Blue (Hydrogen Interaction), Pink and Lilac (Hydrophobic Interaction) and Orange and Red (Steric Interactions). **Residues:** Ser (Serine), Pro (Proline), Phe (Phenylalanine), Val (Valine), Lys (Lysine), Ile (Isoleucine), Cys (Cysteine), Tyr (Tyrosine), His (Histidine), Asp (Aspartate), Arg (Arginine), Asn (Asparagine), Leu (Leucine), Ala (Alanine).

When observing the interactions of the compounds NC74 (I) and NC76 (II), it can be seen that the atoms that interact with amino acid residues were mostly oxygen, benzene ring carbons, and fluorine atoms. This can be seen, for example, with the residues of Tyr 110 in compound NC74, as well as residues of Ser 111, Ser 112, Ser 146 and Leu 18 in compound NC76. Steninbreinner (2013) reports the occurrence of tyrosine, leucine and serine residues in the antioxidant functions and in the metabolism of selenium, through selenoproteins [80]. NC74 compound showed hydrogen bonding interactions (dashed lines in green and blue), hydrophobic interactions (dashed lines in pink) and steric interactions (dashed lines in red and orange). For the enzyme CYP-51 (3L4D), hydrogen bonds were visualized in the fluorine atoms (F) and in the oxygen atoms (O) of the carbonyl group, as well as in the oxygen atoms (O) of the rings, the residues involved were Ser 225 (1 interaction), Val 42 (1 interaction), Lys 219 (1 interaction), Ile 45 (1 interaction) and Ile 16 (1 interaction). Hydrophobic interactions were observed on the carbon (C) atoms of the aromatic rings through residues Pro 80 (1 interaction), Phe 41 (1 interaction), Val 42 (1 interaction), Pro 221 (1 interaction), and Ile 16 (1 interaction). For the enzyme Trypanothione Reductase (PDB: 5EBK), hydrogen bonds were observed with the oxygen atoms (O) of the carbonyl groups through the residues Tyr 110 (1 interaction), Ser 14 (1

interaction), and two interactions were also observed in the aromatic ring, with a hydrophobic interaction established by residue Val 53 and a steric interaction established by residue Cys 57. In the Pteridine Reductase target, only hydrogen bonds and hydrophobic interactions were observed, hydrogen bonds were observed in oxygen atoms (O) through residues Asn 109 (1 interaction), Lys 198 (1 interaction), Ser 111 (1 interaction), Phe 113 (1 interaction), Asp 142 (1 interaction) and Ser 112 (1 interaction), with hydrophobic interactions being observed in the methoxy group that constitutes the aromatic ring through residues His 38 (1 interaction), Arg 39 (1 interaction) and Tyr 114 (1 interaction). The steric interaction occurred in smaller numbers and was visualized by the residue Tyr 194 (1 interaction). For the UDP-Glucose enzyme, a reduced number of interactions were observed that included the Hydrogen (H) atoms of the amine group (NH) and the Oxygen (O) atoms through the residues Pro 372 (1 interaction) and Arg 248 (2 interactions). The hydrophobic interactions were established by residues Val 370 (1 interaction) and Arg 248 (three interactions). For the compound NC76, interactions of the hydrophobic type (dashed lines in pink), interactions of the type of hydrogen bonds (dashed lines in green) and steric interactions (dashed lines in orange) were visualized in the CYP-51 enzyme (PDB : 3L4D) hydrophobic interactions were observed with the groups (CH2) through the residue Pro 221 (1 interaction) and Lys 210 (1 interaction), demonstrating a greater contribution of the non-polar groups of the compound in the interaction with the target. For the enzyme Trypanothione Reductase (PDB: 5EBK), hydrophobic interactions were observed with the carbon atoms (C) of the aromatic rings and with the groups (CH2) through residues Ile 106 (3 interactions), Val 58 (2 interactions) and Val 53 (1 interaction). Hydrogen bonding was observed on the Oxygen atom of the carbonyl group through the residue Tyr 110 (1 interaction). For the enzyme Pteridine reductase, a much larger number of interactions were observed, which corresponded to hydrophobic interactions with the groups (CH2) of the compound through residues Pro224 (2 interactions), His 38 (1 interaction) Leu 18 (1 interaction). Hydrogen bonds were more prevalent being observed in the Oxygen atoms (O) of the carbonyl groups, in the Oxygen atoms (O) of the hydroxyl groups (OH) and in the Fluorine atoms (F), the residues involved with -understood Ala 110 (1 interaction), Ser 111 (1 interaction), Ser 146 (1 interaction), Ser 112 (1 interaction), His 38 (1 interaction) and Asp 142 (2 interactions). Only one steric interaction was observed with the carbon atoms (C) of the aromatic ring through the residue Asp 142 (1 interaction).

For these and the rest of the ligand interactions, DSC analysis was performed to ensure the scores were independent of the molecular weight. This measure was successful and suggested reliable results. Overall, molecules with lower energy scores typically featured a lower MW, as well as hydrogren bonding and steric interactions. UDP-Glucose binding displayed the ligand and the molecule NC60 with the highest score (−146.61 Kcal/mol and −152.19 Kcal/mol respectivelly) presented all three types of interactions investigated (hydrogen interactions, interactions hydrophobic and steric interactions). The ligand showed Hydrogen bonds to the Oxygen (O) atoms of the structure through the residues Gly 409 (1 interaction) and Asp 404 (1 interaction).) of the aromatic rings through Ser 374 (1 interaction) and Arg 248 (1 interaction). The steric interactions were the most comprehensive, comprising the oxygen atoms (O) of the hydroxyl groups (OH), as well as the carbon atoms (C) of the rings through residues His 410 (1 interaction), Arg 248 (1 interaction) and Ser 374 (1 interaction). The compound NC60 presented Hydrogen bonds with the Nitrogen atoms (N) of the amine group (NH), with the Hydrogen atoms (H) of the aromatic rings and with the Oxygen atoms (O) of the carbonyl group, being the residues involved corresponding to Gly 409 (1 interaction) and Arg 373 (1 interaction), whereas hydrophobic and steric interactions were observed in the carbon atoms (C) of the aromatic rings through residues His 410 (2 interactions), Arg 248 (2 interactions), Leu 403 (1 interaction), Arg 373 (1 interaction) and His 408 (2 interactions). Here in, we found that NC60 bound to the four structures mentioned above through Gly 409, His 410 and His 408 (as seen with other binding sites in Figure S6). Trypanothione

reductase (PDB: 5EBK) binding occurred presented a score of −45.11 Kcal/mol, in addition to hydrophobic (dashed lines in pink) and steric (dashed lines in orange) interactions that occurred with the chlorine atom (Cl) and with the carbon atoms (C) of the aromatic ring that involved residues Cys 57 (2 interactions), Cys 52 (1 interaction) and Val 53 (2 interactions). The compound NC74 (H) showed an affinity score of -65.01 Kcal/mol, in addition to hydrogen interactions with the oxygen atoms (O) of the carbonyl group through residues Ser 14 (1 interaction) and Tyr 310 (1 interaction), in addition, it was observed that the carbon atoms of the aromatic ring presented hydrophobic interactions through the residue Val 53 (1 interaction) and steric interactions through the residue Cys 57 (1 interaction). Similar interactions occurred between the PDB linker and the NC74 compound that spanned residues Val 53 and Cys 57. This corresponds to literature reported NC 74 binding at Tyr 110 and Val 53 that was suggested to control for the correct positioning of the cavity in the enzyme [24]. Finally, Pteridine Reductase (E) binding occurred at Leu 18, in addition, the ligand presented a score of -141.03 Kcal/mol establishing hydrogen interactions with the Oxygen (O) atoms of the hydroxyl (OH) and methoxyl (OCH3) groups through the residues Asn 109 (2 interactions), Pro 224 (1 interaction) and Gly 225 (1 interaction), the steric interactions occurred only once through the residue Asp 161 (1 interaction) with the atoms of the aromatic ring demonstrating the internal interactions, while the hydrophobic interactions were the most numerous occurring on the Carbon (C) atoms of the aromatic rings through the residues Leu 18 (1 interaction), Pro 224 (1 interaction), Leu 188 (1 interaction), Tyr 283 (1 interaction), Leu 226 (1 interaction) and Met 183 (2 interactions). The compound NC76 (F) presented hydrogen bonds that demonstrate the polar characteristics of the compounds in the oxygen atoms (O) of the carbonyl groups and the methoxy groups (OCH3) through the residues Ala 110 (1 interaction), Ser 111 (1 interaction), Ser 146 (1 interaction), Ser 112 (1 interaction), His 38 (1 interaction) and Asp 142 (3 interactions), only one steric interaction was visualized (dotted line in orange) through the Asp residue 142 (1 interaction), the hydrophobic interactions are more numerous since they involve the carbon atoms (C) of the aromatic rings and groups (CH2) through residues Pro 224 (2 interactions), Leu 18 (1 interaction), His 38 (1 interaction) and Tyr 114 (1 interaction). while CYP 51 (PDB: 3L4D) did not display similar residue alignment with the molecules tested. Hypothesize that this phenomenon could also be caused by false negatives in molecular docking analyses, and cite the use of decoys in MD simulations. These decoys evaluate the reproducability of the binding modes and peptides, but may hinder results or also because the test compound is located in a different site than the PDB Fluconazole linker. Fluconazole presented a score of −116.85 Kcal/mol and only hydrophobic interactions (dashed lines in pink) and hydrogen bonding (dashed lines in green) were observed in the PDB ligand Fluconazole (A), the hydrogen bonds were visualized in the Nitrogen atoms (N) of the aromatic ring and the Fluorine atoms (F) through the Ala residue 290 (2 interactions), whereas the hydrophobic interaction corresponded to the Ala residue 286 (2 interactions), being visualized in the (C) atoms of the aromatic rings. The compound NC62 (B) presented a score of −136.46 Kcal/mol and, unlike the control Fluconazole, presented a steric interaction (dotted line in orange) with the carbon atom (C) of the aromatic ring through the residue Lys 219 (1 interaction), in this same group hydrophobic interactions were visualized through residues Trp 216 (1 interaction) and Leu 220 (1 interaction). Hydrogen bonds occurred only once and comprised residues Lys 219 (1 interaction).

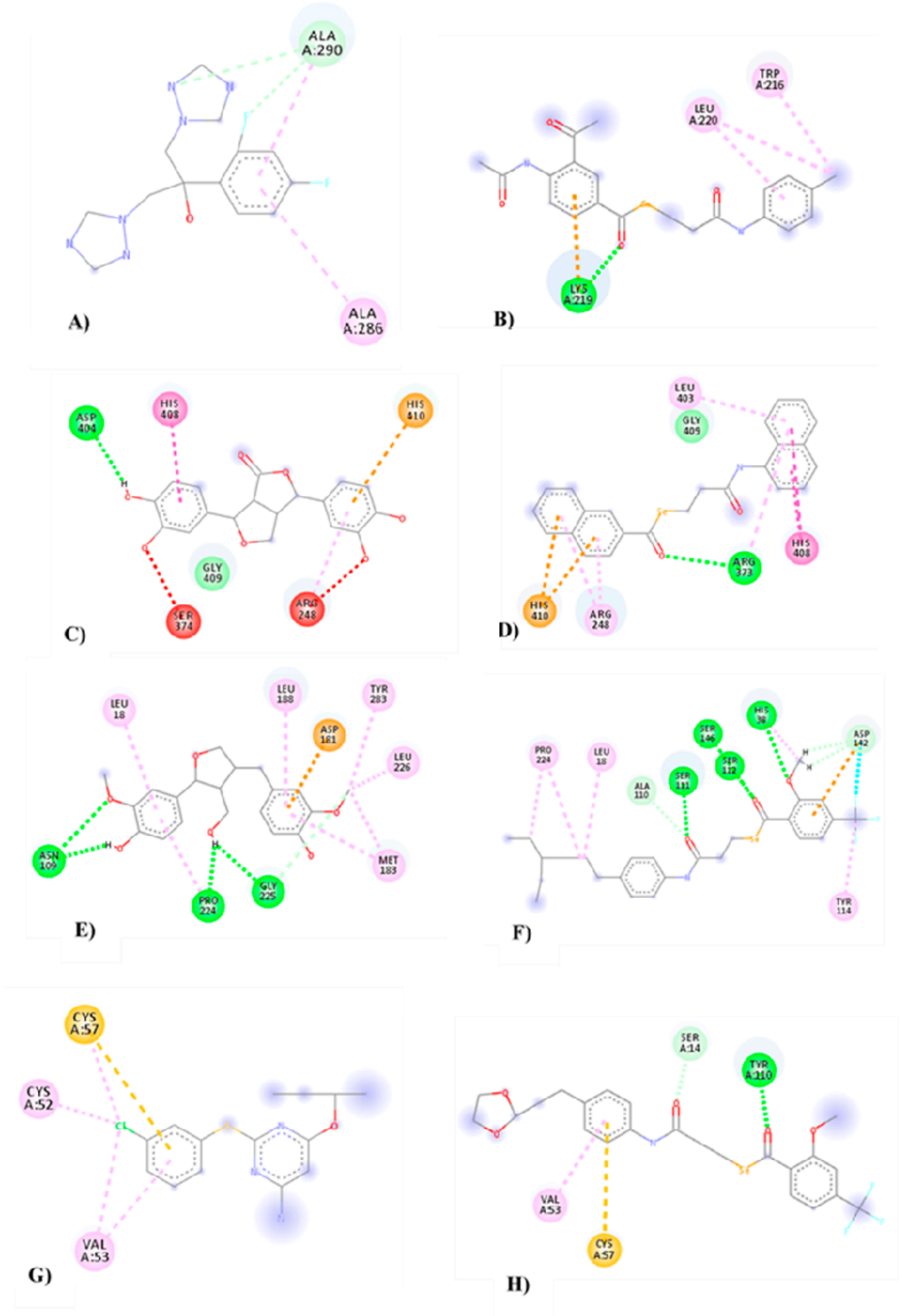

Figure S6. 2D representation of interactions against *L. infantum*. Legend: A) 3L4D protein ligand; B) NC62 molecule; C) UDP binder; D) NC60 molecule; E) Pteridine Reductase Ligand; F) NC76 molecule; G) 5EBK ligand and H) NC74 molecule. **Colors:** Green (Hydrogen Interaction), Pink (Hydrophobic Interaction), Red and Orange (Steric Interaction). **Residues:** Ala (Alanine), Lys (Lysine), Leu (Leucine), Trp (Tryptophan), Asp (Aspartate), His (Histidine), Ser (Serine), Arg (Arginine), Gly (Glycine), Asn (Asparagine), Tyr (Tyrosine), Pro (Proline), Cys (Cysteine), Val (Valine).

Leishmania amazonensis

For *L. amazonensis*, molecular docking simulations were performed with enzymes obtained by homology, since there is no X-ray crystallography available for this species, whose validation procedure was described above. The selected enzymes include Arginase, which is a trimeric manganese-containing enzyme that hydrolyses the amino acid

L-arginine into L-ornithine and urea. L-ornithine is used to synthesize polyamines and is essential for parasite growth and host infection. Polyamines are also essential for the production of trypanothione, an important antioxidant agent in the control of reactive oxygen species (ROS). In fact, the expression and activity of arginase in Leishmania contribute to a higher infectivity of the parasite and play an important role in the pathogenicity of the infection [71]; Lanosterol; Trypanothione Reductase and Dihydrorotate dehydrogenase represents a flavoenzyme that catalyzes the fourth reaction of the pyrimidine biosynthetic pathway, which plays vital roles in cells, especially in the biosynthesis of DNA and RNA. Thus, this enzyme stands out as a new key molecular target for parasites that cause Neglected Diseases [81]. Table S2 features the Moldock energy scores obtained by the compounds and the DSC calculations.

While all compounds studied delivered negative scores, none scored better than the PDB ligands. One possible explanation for this is the difference in the molecular weight of the PDB ligands and molecules in question. The rankings demonstrated a strong preference for trypanothione reductase and dihydroorotate reductase by these ligands and identified these as the best targets among all those studied in regards to *L. amazonensis*. The major residues and interactions of these and the other proteins is displayed in Figure S7. In general, lower energy combinations featured hydrogen bonds and steric interactions, similar to trends in the *L. infantum* simulations. While no coinciding residues were identified, this may be due to the factors aforementioned and/or the fact that the enzymes were constructed by homology models.

For the enzymes of the *L. amazonesis* species, Docking Score Correction (DSC) calculations were performed. In the Arginase enzyme, the ligand (A) obtained score values equivalent to −164.07 Kcal/mol DSC of −0.34, hydrogen interactions were observed (dashed lines in green) in the hydroxyl groups (OH) of the sugar units and the chain aliphatic of the compound through residues Ser 150 (1 interaction), His 154 (1 interaction), Gly 256 (2 interactions), His 28 (1 interaction), Glu 196 (1 interaction) and Gly 147 (1 interaction) , one can see a greater contribution of polar groups in the structure of the ligand. The hydrophobic interactions were visualized only in the atoms (CH2) of the compound through residue Val 149 (3 interactions). The NC37 (B) compound had a score of −106.85 Kcal/mol and a DSC of −0.29. Unlike the Arginase enzyme ligand, compound NC37 showed steric interactions (dashed lines in red) and hydrophobic interactions (dashed lines in pink), demonstrating a greater contribution of non-polar groups in its structure, hydrogen interactions were visualized in atoms of Oxygen (O) from the carbonyl groups through residues Gly 279 (1 interaction) and Leu 281 (1 interaction). Hydrophobic and steric interactions were present in similar atoms comprising Selenium atoms (Se) and Carbon atoms (C) of the cyclic groups, observing the amino acids Val 238 (1 interaction), Arg 280 (1 interaction), Ile 227 (1 interaction), Leu 281 (1 interaction) and Trp 52 (1 interaction).

For the Lanosterol enzyme, the ligand (C) showed hydrophobic interactions (pink dashed lines) and hydrogen bonding interactions (green dashed lines). Hydrogen bonds, similarly to what happened in the enzyme Arginase, were prevalent in the hydroxyl groups (OH) of the sugar unit and the aliphatic chain of the compound through residues Arg 360 (1 residue), Tyr 102 (2 residues), Met 357 (1 residue), Met 459 (1 residue) and Ala 256 (1 residue), demonstrating a greater polarity of this compound, since the hydrophobic interactions were observed in the groups (CH), (CH2) and (CH3) by through residues Cys 422 (1 interaction), Leu 358 (1 interaction), Ala 290 (1 interaction) and Ala 296 (1 interaction). The compound NC38 (D) presented a score of −141.24 Kcal/mol and DSC of −0.38, a greater collaboration of the non-polar groups in its structure is noticeable, since the prevalence of hydrophobic interactions was observed (dashed lines in pink and in purple) and steric interactions (orange dashed lines). These interactions were prevalent in the aromatic ring atoms and were established by residues Ala 345 (1 interaction), Val 432 (2 interactions), Ile 349 (1 interaction), Thr 298 (2 interactions), Leu 429 (2 interactions) and Cys 422 (1 interaction). The hydrogen interactions occurred only on the Oxygen (O)

atoms of the carbonyl groups through residues Thr 294 (1 interaction), Ser 295 (1 interaction) and Gly 428 (1 interaction).

For the enzyme Trypanothione reductase, the ligand (E) presented a Score of −156.04 Kcal/mol and DSC values −2.13, a greater apolar characteristic was noticeable since there was a greater prevalence of hydrophobic interactions through residues Val 58 (1 interaction) , Val 53 (1 interaction), Ile 106 (1 interaction) and Leu 17 (1 interaction) on the Carbon (C) atoms of the chemical structure. Hydrogen interactions were observed on the Oxygen atoms of the structure through the residues Leu 17 (1 interaction) and Ser 14 (1 interaction). The compound NC32 presented a score of −58.29 Kacal/mol and DSC of −6.19 and presented only hydrophobic interactions, demonstrating a greater contribution of non-polar groups in the interaction with the target. The residues involved comprised ile 199 (1 interaction), Arg 287 (1 interaction) and Thr 51 (1 interaction).

Dihydroorotate dehydrogenase was the last mechanism evaluated, with the ligand (G) presenting interactions like hydrogen bonds on the oxygen atoms (O) of the hydroxyl groups (OH) through residues Asp 143 (1 interaction), Ala 146 (1 interaction) and Asn 128 (1 interaction). Hydrophobic interactions were observed in groups (CH2) and (CH3) and (CH) through residues Lys 137 (1 interaction), Val 140 (2 interactions) and Ala 146 (1 interaction). The steric interaction (dotted line in orange) was visualized only on the atoms of the benzene ring through the residue Cys 150 (1 interaction). The compound presented a score of −124.91 Kcal/mol and DSC of −3.91, this one presented a greater occurrence of apolar interactions, since it presented a greater number of hydrophobic interactions and steric interactions, the groups involved were the Bromine atoms (Br) and the carbon (C) atoms of the aromatic rings through the residues Cys 249 (2 interactions), Ile 194 (1 interaction), Ile 226 (1 interaction), Lys 44 (1 interaction) and Cys 131 (1 interaction). Hydrogen bonds occurred in smaller numbers comprising the oxygen atoms (O) of the carbonyl group through residues Asn 68 (1 interaction), Gly 222 (1 interaction) and Ser 66 (1 interaction).

L. amazonensis

Table S2. Data on the relationship between ligand-receptor interaction energies and molecular weigh.

ID	PM	Arginase		Lanosterol		Trypanotione Reductase		Dihydroorotate dehydrogenase	
		MS	MS/PM	MS	MS/PM	MS	MS/PM	MS	MS/PM
NC30	333,01	–87,44	–0.26	–111.90	–0.34	–76.18	–4.37	–129.14	–2.58
NC31	347,04	–94,32	–0.27	–115.74	–0.33	–85.72	–4.05	–130.37	–2.66
NC32	361,05	–94,09	–0.26	–133.56	–0.37	–58.29	–6.19	–127.79	–2.83
NC33	362,05	–93,89	–0.26	–136.82	–0.38	–85.60	–4.23	–135.59	–2.67
NC34	363,04	–100,04	–0.28	–123.59	–0.34	–82.50	–4.40	–135.69	–2.68
NC36	366,99	–88,09	–0.24	–125.28	–0.34	–71.38	–5.14	–130.52	–2.81
NC37	372,03	–106,85	–0.29	–136.62	–0.37	–80.12	–4.64	–138.15	–2.69
NC38	372,03	–99,13	–0.27	–141.24	–0.38	–103.13	–3.61	–144.01	–2.58
NC39	372,03	–98,42	–0.26	–127.59	–0.34	–95.34	–3.90	–130.82	–2.84
NC40	375,07	–98,68	–0.26	–128.58	–0.34	–91.19	–4.11	–135.26	–2.77
NC41	378,01	–91,87	–0.24	–133.66	–0.35	–78.40	–4.82	–134.97	–2.80
NC42	383,01	–109,44	–0.29	–141.52	–0.37	–97.53	–3.93	–146.75	–2.61
NC44	383,01	–102,24	–0.27	–132.19	–0.35	–109.59	–3.50	–140.01	–2.74
NC45	390,08	–102,07	–0.26	–132.13	–0.34	–78.65	–4.96	–141.08	–2.77
NC46	392,02	–91,53	–0.23	–127.31	–0.32	–91.96	–4.26	–141.81	–2.76

NC47	392,02	–98,40	–0.25	–129.65	–0.33	–81.70	–4.80	–130.45	–3.01
NC48	392,02	–86,41	–0.22	–129.38	–0.33	–68.93	–5.69	–121.40	–3.23
NC49	393,03	–90,82	–0.23	–132.96	–0.34	–78.18	–5.03	–134.88	–2.91
NC50	397,05	–103,40	–0.26	–138.16	–0.35	–81.86	–4.85	–144.80	–2.74
NC51	404,32	–102,29	–0.25	–127.57	–0.32	–103.25	–3.92	–139.07	–2.91
NC53	410,94	–91,04	–0.22	–123.92	–0.30	–76.66	–5.36	–135.18	–3.04
NC58	424,95	–87,86	–0.21	–137.96	–0.32	–81.75	–5.20	–140.78	–3.02
NC59	425,00	–101,82	–0.24	–131.79	–0.31	–76.85	–5.53	–136.73	–3.11
NC60	433,05	–115,20	–0.27	–150.21	–0.35	–97.57	–4.44	–148.78	–2.91
NC67	488,84	–96,87	–0.20	–127.94	–0.26	–91.32	–5.35	–124.91	–3.91
Ligante	477,32	–164,07	–0.34	–185.23	–0.39	–156.04	–2.13	–163.78	–2.24

Legend: MS = MolDock Score [kj.mol-1]; MW = Molecular Weight; MS/PM = Relationship between interaction energy and molecular weight [kj.mol-1].

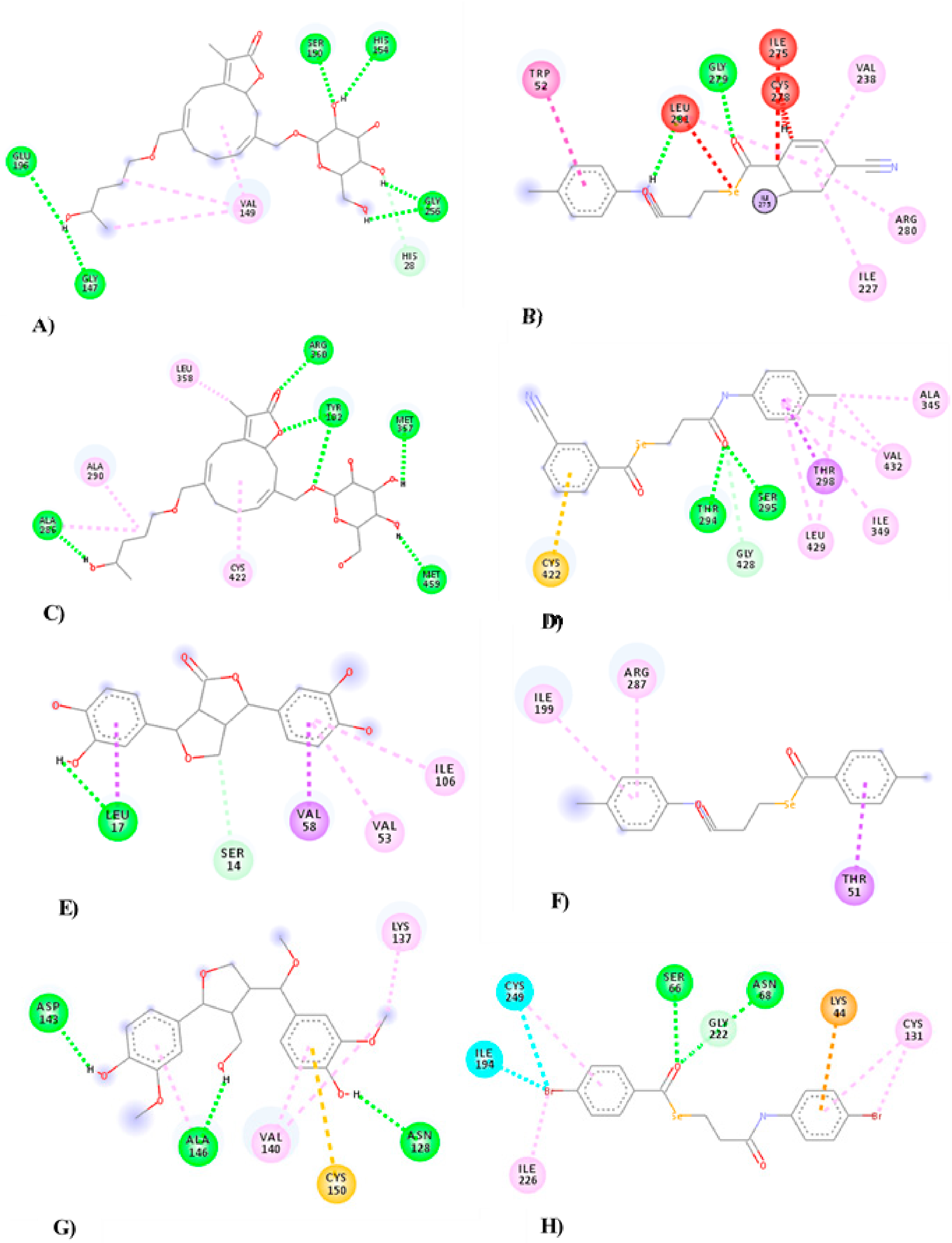

Figure S7. 2D representation of interactions against *L. amazonensis*. Legend: **A**) Arginase protein ligand; **B**) NC37 molecule; **C**) Lanosterol Binding; **D**) NC38 molecule; **E**) Trypanotione reductase ligand; **F**) NC32 molecule; **G**) Dihydroorotate dehydrogenase ligand and **H**) NC67 molecule. **Colors:** Green and Blue (Hydrogen Interaction), Pink and Lilac (Hydrophobic Interaction) and Orange and Red (Steric Interaction). **Residues:** Glu (Glutamic Acid), Gly (Glycine), Val (Valine), Ser (Serine), His (Histidine), Trp (Tryptophan), Leu (Leucine), Ile (Isoleucine), Cys (Cysteine), Arg (Arginine), Ala (Alanine), Tyr (Tyrosine), Met (Methionine), Thr (Threonine), Asp (Aspartic Acid), Lys (Lysine), Asn (Asparagine).

Leishmania braziliensis

The MD simulations of *L. braziliensis* were similarly obtained by homology, because it does not present a 3D structure of specific enzymes for this species, with the previously described validation procedure. The evaluated enzymes included: Trypanothione Re-

ductase, Pteridine Reductase, UDP-Glucose Pyrophosphorylase and Glycerol 3-phosphate Dehydrogenase, this enzyme plays an important role in the glycolytic pathway, and also indirectly participates in the via pentose phosphate catalyzing the reversible oxidation (redox reaction) of L-glycerol-3-phosphate (L-G3P) to dihydroxy-acetone phosphate (DHAP) using a nicotinamide adenine dinucleotide (NADH) molecule as a cofactor. The pentose phosphate plays an important role in parasite defense against reactive oxygen species produced by mammalian hosts, which are neutralized by NADH-dependent reactions. In addition, the glycolytic pathway produces amino acids and sugars, which are a rich source of carbon and energy for the amastigote forms (parasitic form) [73]. Table S3 features the Moldock energy scores obtained by the compounds and the DSC calculations. Overall, all the compounds displayed negative scores. NC52 (−115.5 Kcal/mol), NC65 (−119.0 Kcal/mol) and NC66 (−117.6 Kcal/mol), as well as NC45 (−88.30 Kcal/mol), NC60 (−89.89 Kcal/mol) and NC62 (−95.81 Kcal/mol), scored lower than the ligand for Trypanothione Reductase and Glycerol-3-Phosphate, respectively. The DSC was less insightful in this area given the nearly comprable molecular mass of the ligand and the molecules in question. However, a favorable result was obtained for Glycerol-3-Phosphate with NC62 (−0.21), NC45 (−0.22) and NC28 (−0.22).

The interactions between the test molecules and the active site of the enzyme are shown in Figure S8. In general, all enzymes had coinciding residues and lower energy scores were partial to hydrogen bonds and hydrophobic interactions. Specifically, NC52 coincided with the Trypanothione Reductase enzyme at Cys 52 and Asp 357, and the ligand had a score of −115.2 Kcal/mol and its interactions comprised hydrogen bonds (dotted line in green) with the Oxygen (O) atoms of the compound through Glu residues 202 (1 interaction), Asp 327 (1 interaction), Cys 57 (1 interaction) guaranteed a more polar characteristic to the compound, in addition, hydrophobic interactions (dashed line in pink) and steric interactions (dashed line in orange) were visualized) with the atoms of the benzene ring through the residues Cys 52 (1 interaction) and Asp 327 (1 interaction). The compound NC52 (B) presented an affinity score of −115.5 Kcal/mol of hydrogen bonding interactions through the Hydrogen (H) atoms of the amine group (NH) through the residues Ala 365 (1 interaction) and Lys 60 (1 interaction), Thr 335 (1 interaction), in addition, hydrophobic and steric interactions were observed in the atoms of the aromatic rings through the residues Cys 57 (1 interaction), Cys 52 (1 interaction), Asp 327 (1 interaction) and Ward 338 (1 interaction).

NC66 (D) aligned with the Pteridine Reductase enzyme (C) at the Pro 230 and Leu 18. The ligand (C) showed a greater amount of nonpolar interactions, since it showed hydrophobic interactions (dashed lines in pink) and steric interactions (dashed lines in red and orange) on the atoms of the aromatic rings through the residues Leu 188 (1 interaction), Phe 191 (1 interaction), Asp 181 (1 interaction), Phe 299 (1 interaction) and Pro 230 (1 interaction). The hydrogen interactions were observed in the Oxygen atoms of the Hydroxyls (OH) through the residues Ser 227 (1 interaction), Met 183 (1 interaction) and Tyr 241 (1 interaction). The compound NC66 presented a score of -138.8 Kcal/mol, in addition, it presented hydrophobic interactions, mostly being located in the methyl groups (CH3) and in the atoms of the aromatic rings through the residues Tyr 194 (1 interaction), Leu 188 (2 interactions), pro 230 (1 interaction) and Tyr 114 (2 interactions). Hydrogen interactions were observed in residues Arg 18 (1 interaction), Leu 226 (1 interaction), Tyr 241 (1 interaction), Gln 186 (1 interaction).

UDP-Glucose pyrophosphorylase and NC62 overlapped at Ser 375, Arg 248, and His 411. The ligand had a score of −125.0 Kcal/mol, making it possible to see hydrogen interactions (dashed lines in green) with the oxygen atom (O) of the hydroxyl group (OH) through the residue Ser 75, in addition, hydrophobic (dashed lines in pink) and steric (dashed lines in orange) interactions were observed, mostly located in the aromatic groups through the residues Arg 374 (1 interaction), Arg 248 (1 interaction) and His 411 (1 interaction). The compound NC62 presented a score of −87.70 Kcal/mol, with hydrogen interactions being visualized on the Hydrogen atoms (H) of the amine group (NH) and

on the Oxygen atoms (O) of the carbonyl group through Ser 375 residues (1 interaction), Asp 406 (1 interaction) and His 409 (1 interaction), the Selenium atom (Se) and the aromatics ring atoms were involved in the steric interactions (dashed lines in orange and red) and in the hydrophobic interactions (pink dashed lines) through the amino acids His411 (1 interaction), Arg248 (2 interactions) and Arg 374 (2 interactions).

Finally, glycerol-3-phosphate and NC62 had identical residues at Phe 97 and Met 46. The ligand showed affinity values around −47.67 Kcal/mol, in addition it showed hydrophobic interactions (dashed lines in pink with the atoms of Bromine (Br) and with the atoms of the aromatic rings through the residues Phe 101 (1 interaction), Trp 44 (1 interaction) Phe 97 (3 interactions), Met 46 (2 interactions) and Ile 93 (1 interaction), also steric interactions were observed (dashed lines in orange) through the Met 46 residues (2 interactions). The compound NC62 presented a score value of -95.81 Kcal/mol, showing hydrogen interactions with the oxygen atoms (O) carbonyl group through residue Lys 104 (1 interaction). The aromatic rings were the groups that most formed interactions, these being hydrophobic interactions (dashed lines in li-lac and pink through residues Phe 97 (1 interaction) and Trp 44 (1 interaction), plus a hydrophobic interaction with r Met residue 46 (1 interaction).

L. braziliensis

Table S3. Data on the relationship between ligand-receptor interaction energies and molecular weight for *L. braziliensis.*

ID	PM	Trypanothione Reductase		Pteridine Reductase		UDP-Glucose-Pyrophosphorylase		Glycerol-3-Phosphate	
		MS	MS/PM	MS	MS/PM	MS	MS/PM	MS	MS/PM
NC28	295,17	−95.27	−0.32	−99.14	−0.33	−82.46	−0.27	−66.83	−0.22
NC32	361,05	−87.16	−0.24	−116.8	−0.32	−87.70	−0.24	−57.83	−0.16
NC33	362,05	−90.59	−0.25	−116.2	−0.32	−95.31	−0.26	−55.19	−0.15
NC35	363,04	−87.07	−0.23	−116.8	−0.32	−101.2	−0.27	−44.46	−0.12
NC37	372,03	−88.34	−0.23	−117.4	−0.31	−95.41	−0.25	−68.74	−0.18
NC40	362,05	−95.12	−0.26	−111.8	−0.32	−91.96	−0.26	−25.69	−0.07
NC41	378,01	−86.21	−0.22	−126.7	−0.33	−89.72	−0.23	−42.16	−0.11
NC45	390,08	−92.92	−0.23	−117.1	−0.30	−94.03	−0.24	−88.30	−0.22
NC46	392,02	−96.43	−0.24	−118.3	−0.30	−91.70	−0.23	−53.14	−0.13
NC47	392,02	−98.37	−0.25	−104.6	−0.26	−100.3	−0.25	−83.99	−0.21
NC48	392,02	−93.53	−0.23	−107.2	−0.27	−92.55	−0.23	−30.25	−0.07
NC49	393,03	−89.47	−0.22	−118.5	−0.30	−89.06	−0.22	−83.7	−0.21
NC50	397,05	−96.65	−0.24	−118.5	−0.29	−97.14	−0.24	−69.85	−0.17
NC51	404,32	−99.78	−0.24	−108.8	−0.26	−105.3	−0.26	−41.08	−0.10
NC52	406,04	−115.5	−0.28	−115.0	−0.28	−104.9	−0.25	−73.30	−0.18
NC53	410,94	−82.53	−0.2	−111.2	−0.27	−85.27	−0.20	−80.34	−0.19
NC54	419,11	−93.38	−0.22	−117.5	−0.28	−82.90	−0.19	−65.02	−0.15
NC55	422,99	−91.49	−0.21	−125.2	−0.29	−91.29	−0.21	−44.06	−0.10
NC56	422,99	−104.9	−0.24	−136.1	−0.32	−85.67	−0.20	−65.11	−0.15
NC57	422,99	−87.20	−0.2	−119.9	−0.28	−107.1	−0.25	−77.79	−0.18
NC60	433,05	−101.1	−0.23	−120.8	−0.27	−96.66	−0.22	−89.89	−0.20
NC61	434,36	−99.21	−0.22	−121.8	−0.28	−102.9	−0.23	−45.41	−0.10

NC62	446,07	–113.6	–0.25	–129.4	–0.29	–108.5	–0.24	**–95.81**	–0.21
NC65	478,41	**–119.0**	–0.24	–131.3	–0.27	–94.61	–0.19	–81.00	–0.16
NC66	478,41	–117.6	–0.24	–138.8	–0.29	–101.4	–0.21	–69.60	–0.14
Lig	346,33	–115.2	–0.33	–154.1	–0.44	–125.0	–0.36	–47.67	–0.20

Legend: MS = MolDock Score [kj.mol-1]; MW = Molecular Weight; MS/PM = Relationship between interaction energy and molecular weight [kj.g-1].

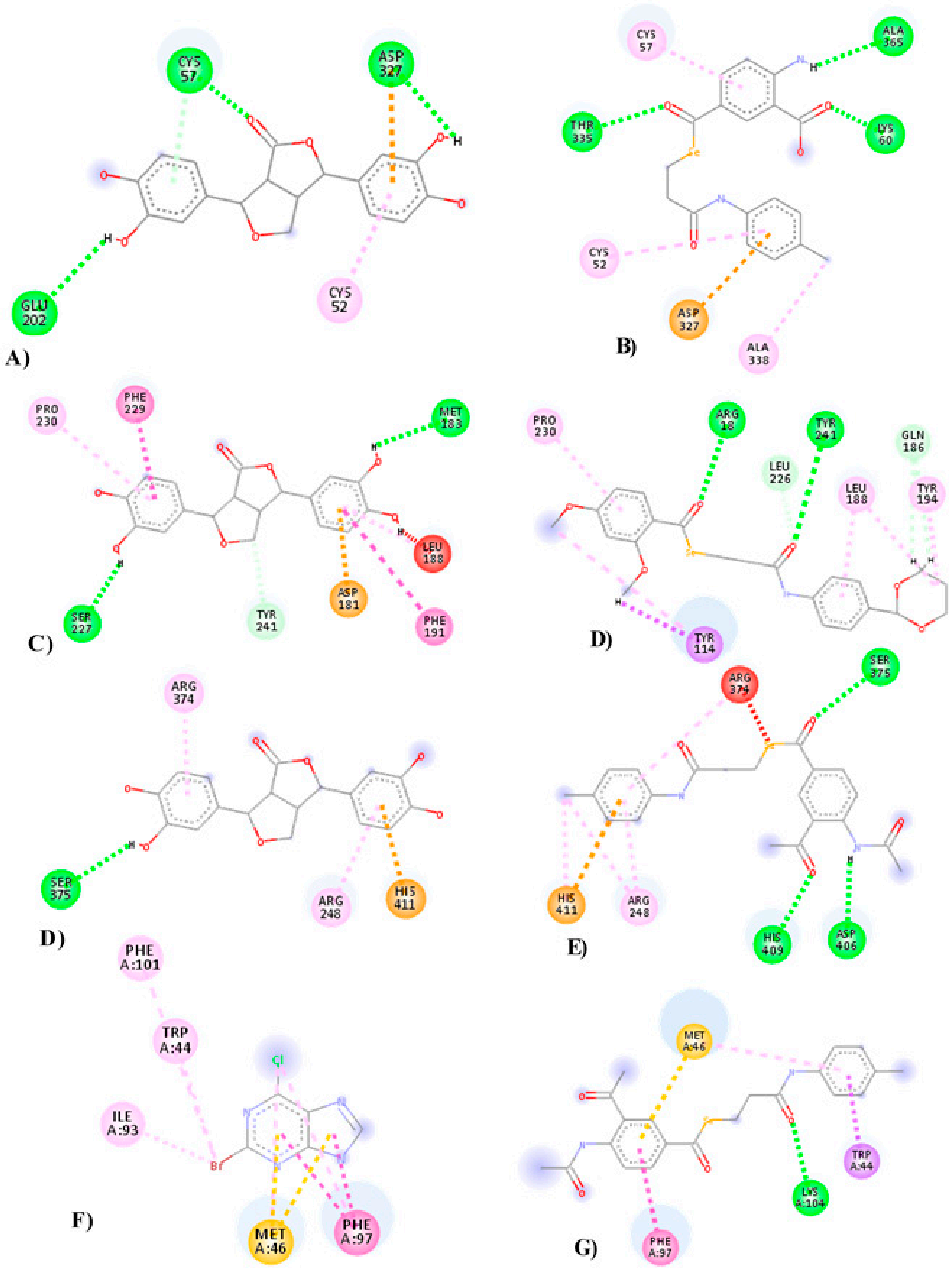

Figure S8. 2D representation of interactions against *L. braziliensis*. Legend: **A**) Trypanothione Reductase protein ligand; **B**) NC52 molecule; **C**) Pteridine Reductase ligand; **D**) NC66 molecule; **E**) UDP-Glucose pyrophosphorylase ligand; **F**) NC62 molecule; **G**) Glycerol-3-phosphate binder and **H**) NC62 molecule. **Colors:** Green (Hydrogen interaction), Pink and lilac (hydrophobic interaction) and Orange and red (steric interaction). **Residues:** Cys (Cysteine), Glu (Glutamic Acid), Asp (Aspartic Acid), Thr (Threonine), Lys (Lysine), Ala (Alanine), Pro (Proline), Phe (Phenylalanine), Met (Methionine), Ser (Serine), Tyr (Tyrosine), Leu (Leucine), Arg (Arginine), Gln (Glutamine), His (Histidine), Ile (Isoleucine), Trp (Tryptophan).

Leishmania major

For *L. major*, MD simulations were performed with enzymes obtained through homology modeling and 3D crystallographic structure. The enzymes used included: Glycerol 3-phosphate, Trypanothione Reduc-tase, N-myristoyl transferase linker (PDB: 6QDF) which catalyzes the transfer of the 14-carbon saturated fatty acid myristate of myristoyl-CoA (MyrCoA) to the ami-no -terminal glycine of a subset of proteins. This predominantly co-translational modification contributes to the targeting of substrate proteins to membrane sites, in addition to facilitating protein-protein interactions [82,83] and UDP-Glucose Pyrophosphorylase Linker (PDB: 5NZM) [84]. Table S4 features the Moldock energy scores obtained by the compounds and the DSC calculations. Again, all compounds performed preferably. For the enzyme Glycerol-3-phosphate, the best scores were obtained for the compounds NC76 (−154.1 Kcal/mol), NC72 (−142.3 Kcal/mol) and NC74 (−138.8 Kcal/mol), and likewise NC72 (−137.3 Kcal/mol) and NC60 (−130.0 Kcal/mol) were the best for the Trypanothione Reductase enzyme. For N-myrystoiltransferase (PDB: 6QDB), NC69 (−110.0 Kcal/mol) presented better scores than the ligand while NC74 (−73.70 Kcal/mol), NC69 (−69.43 Kcal/mol), and NC60 (−69.06 Kacl/mol) were the strongest with UDP-Glucose-Pyrophosphorylase (PDB: 5NZM). The DSC methodology was only helpful in highlighting the interactions between UDP-Glucose-Pyrophosphorylase (5NZM) and NC04 (−0.27), and N-Myrystoyltransferase with NC04 (−0.20), NC32 (−0.15) and NC40 (−0.16).

The interactions between the test molecules and the active site of the enzyme are depicted in Figure S9. All enzymes had coinciding residues, and as identified with *L. braziliensis*, strong interactions favored hydrogen bonds and hydrophobic interactions, but also steric interactions.

More specifically, Glycerol-3-phosphate and NC76 coincided over the Pro 95, Trp 45 and Ile 47 residues. The ligand showed a score of -136.3 Kcal/mol and hydrophobic interactions (dashed lines in pink), mostly which configures a nonpolar character, these were visualized in the aromatic rings through the residues Tyr 64 (1 interaction), Pro 95 (1 interaction), Ile 94 (1 interaction), Trp 45 (1 interaction), Ile 47 (1 interaction). Hydrogen interactions were observed in the Oxygen atoms (O) of the rings and hydroxyl groups (OH) through the residues Phe 102 (1 interaction) and Ser 24 (1 interaction). The NC76 compound, similarly to the PDB ligand, presented a more apolar character, with hydrophobic interactions (pink dashed lines) being more comprehensive, these were visualized in the atoms of the aromatic rings and in the groups (CH3), (CH2) and (CH) through residues Pro 95 (2 interactions), Phe 27 (1 interaction), Cys 124 (1 interaction), Val 93 (2 interactions), Ile 94 (1 in-interaction), Trp 45 (3 interactions), Ile 47 (1 interaction) Phe 102 (1 interaction) and Phe 93 (1 interaction). Hydrogen bonding (blue dashed line) with Fluorine (F) atoms by Phe 98 residues (1 interaction) were established in smaller amounts.

For the Trypanothione reductase and NC72, Ile 199, Lys 60, Ala 365 and Leu 334 were the strongest. The ligand (C) presented a score of −127.0 Kcal/mol, also showing hydrogen interactions in the hydroxyl groups (OH) through residues Val 55 (1 interaction), Arg 287 (1 interaction), Met 133 (1 interaction) and Ward 365 (1 interaction). In the aromatic rings, it was possible to visualize the occurrence of hydrophobic interactions through residues Ile 199 (1 interaction), Lys 60 (1 interaction), Gly 56 (1 interaction), Cys 57 (1 interaction), Leu 334 (1 inte -ration) and Pro 336 (1 interaction). The compound NC72 presented a score of −137.3 Kcal/mol, showing the importance of the Oxygen and Fluorine (F) atoms of the structure, as well as the Nitrogen (N) atoms of the amine group (NH) for the occurrence of hydrogen bonds through residues Cys 57 (1 interaction), Thr 335 (1 interaction), Ala 395 (1 interaction), Ser 178 (1 interaction) and Gly 56 (1 interaction). The hydrophobic interactions were more numerous and were established by the amino acids Ile 199 (3 interactions), Phe 203 (1 interaction), Lys 60 (1 interaction), Leu 334 (1 interaction), Ala 365 (1 interaction), Cys 364 (1 interaction), Phe 367 (1 interaction) and Cys 57 (1 interaction).

The enzyme N-myristoyl transferase (PDB: 6QDF), correlated to the His 219, Tyr 217, Tyr345 and Leu 399 of NC 69. The ligand had a score of -108.1 Kcal/mol, and it was also noticeable that the non-polar characteristics prevailed, since hydrophobic interactions were observed (pink dashed lines) through residues ile 328 (1 interaction), Tyr 345 (2 interactions), Leu 399 (1 interaction), Tyr 217 (3 interactions), His 219 (2 interactions) and Phe 232 (1 interaction). The only hydrogen interaction visualized corresponded to the Nitrogen (N) atom of the amine group (NH) through the residue His 398 (1 interaction). Still, steric interactions were visualized (red dashed lines) across Tyr 217 residues (2 interactions). The compound NC69 presented a score of -110.9 Kcal/mol. In addition to interactions of Hydrogen with Fluorine (F) atoms and with Oxygen (O) atoms of the carbonyl group through residues His 219 (1 interaction), Tyr 92 (1 interaction). Similarly to the ligand, the hydrophobic interactions were broader and involved the amino acids Tyr 217 (2 interactions), Tyr 345 (2 interactions), Leu 399 (1 interaction), His 219 (1 interaction), Val 419 (1 interaction) and Val 378 (1 interaction).

Finally, UDP-Glucose pyrophosphorylase (PDB: 5NZM) and NC74 featured identical residues at Arg 248 and Arg 373. The PDB ligand had a score of −63.86 Kcal/mol and was nonpolar, as only hydrophobic interactions were seen (lines dashed in pink) through residues Arg 373 (4 interactions) and Arg 248 (2 interactions). The compound NC74, on the other hand, showed an affinity of −73.70 Kcal/mol, where hydrogen interactions were visualized (dashed lines in green) through the Oxygen and Fluorine atoms of the structure through the amino acids His 410 (1 interaction), Pro 411 (1 interaction), Arg 248 (1 interaction), Arg 373 (1 interaction), Val 371 (1 interaction). In addition, a steric interaction was observed through the residue Pro 372 (1 interaction), as well as hydrophobic interactions with the carbon atoms (C) of the structure through the residues Cys 247 (1 interaction), Arg 373 (3 interactions) and Arg 248 (3 interactions).

L. major

Table S4. Data on the relationship between ligand-receptor interaction energies and molecular weight *L. major*.

ID	PM	Glycerol–3–Phosphate		Trypanothione Reductase		6QDO		5NZM	
		MS	MS/PM	MS	MS/PM	MS	MS/PM	MS	MS/PM
NC04	208.04	–72.29	–0.34	–62.87	–0.30	–57.80	–0.27	–41.95	–0.20
NC30	333.01	–97.72	–0.29	–80.93	–0.24	–74.12	–0.22	–39.34	–0.11
NC31	347.04	–99.05	–0.28	–92.23	–0.26	–70.32	–0.20	–48.74	–0.14
NC32	361.05	–107.6	–0.29	–94.26	–0.26	–102.5	–0.28	–57.09	–0.15
NC33	362.05	–109.9	–0.30	–98.60	–0.27	–98.27	–0.27	–43.29	–0.11
NC36	366.99	–101.0	–0.27	–91.86	–0.25	–69.97	–0.19	–47.53	–0.12
NC39	372.03	–113.8	–0.30	–103.4	–0.27	–71.21	–0.19	–39.30	–0.10
NC40	346.33	–107.7	–0.31	–101.4	–0.29	–98.92	–0.28	–56.77	–0.16
NC44	383.01	–106.9	–0.27	–101.3	–0.26	–90.27	–0.23	–43.81	–0.11
NC45	390.08	–109.6	–0.28	–98.86	–0.25	–88.90	–0.22	–38.67	–0.09
NC49	393.03	–114.0	–0.29	–102.0	–0.25	–98.57	–0.25	–54.21	–0.13
NC50	397.05	–107.2	–0.27	–114.2	–0.28	–93.01	–0.23	–52.11	–0.13
NC52	406.04	–117.8	–0.29	–113.1	–0.27	–90.71	–0.22	–53.01	–0.13
NC53	410.94	–105.9	–0.25	–90.01	–0.21	–82.01	–0.19	–56.49	–0.13
NC58	424.95	–107.3	–0.25	–96.26	–0.22	–82.05	–0.19	–49.65	–0.11
NC59	425.0	–118.8	–0.27	–96.22	–0.22	–93.16	–0.21	–51.21	–0.12
NC60	433.05	–114.6	–0.26	–130.0	–0.30	–106.1	–0.24	–69.06	–0.15

NC62	446.07	−132.3	−0.29	−117.2	−0.26	−90.82	−0.20	−61.80	−0.13
NC67	488.84	−107.8	−0.22	−96.22	−0.19	−95.29	−0.19	−53.42	−0.10
NC69	513.1	−132.6	−0.25	−121.8	−0.23	−110.9	−0.21	−69.43	−0.13
NC72	530.41	−141.3	−0.26	−137.3	−0.25	−102.8	−0.19	−54.78	−0.10
NC74	531.07	−138.8	−0.26	−118.7	−0.22	−107.5	−0.20	−73.70	−0.13
NC76	543.14	−154.1	−0.28	−126.2	−0.23	−86.91	−0.16	−46.77	−0.08
Lig	346.33	−136.3	−0.39	−127.0	−0.36	−108.1	−0.27	−63.86	−0.18

Legend: MS = MolDock Score [kj.mol-1]; MW = Molecular weight; MS/PM = Relationship between interaction energy and molecular weight [kj.g-1].

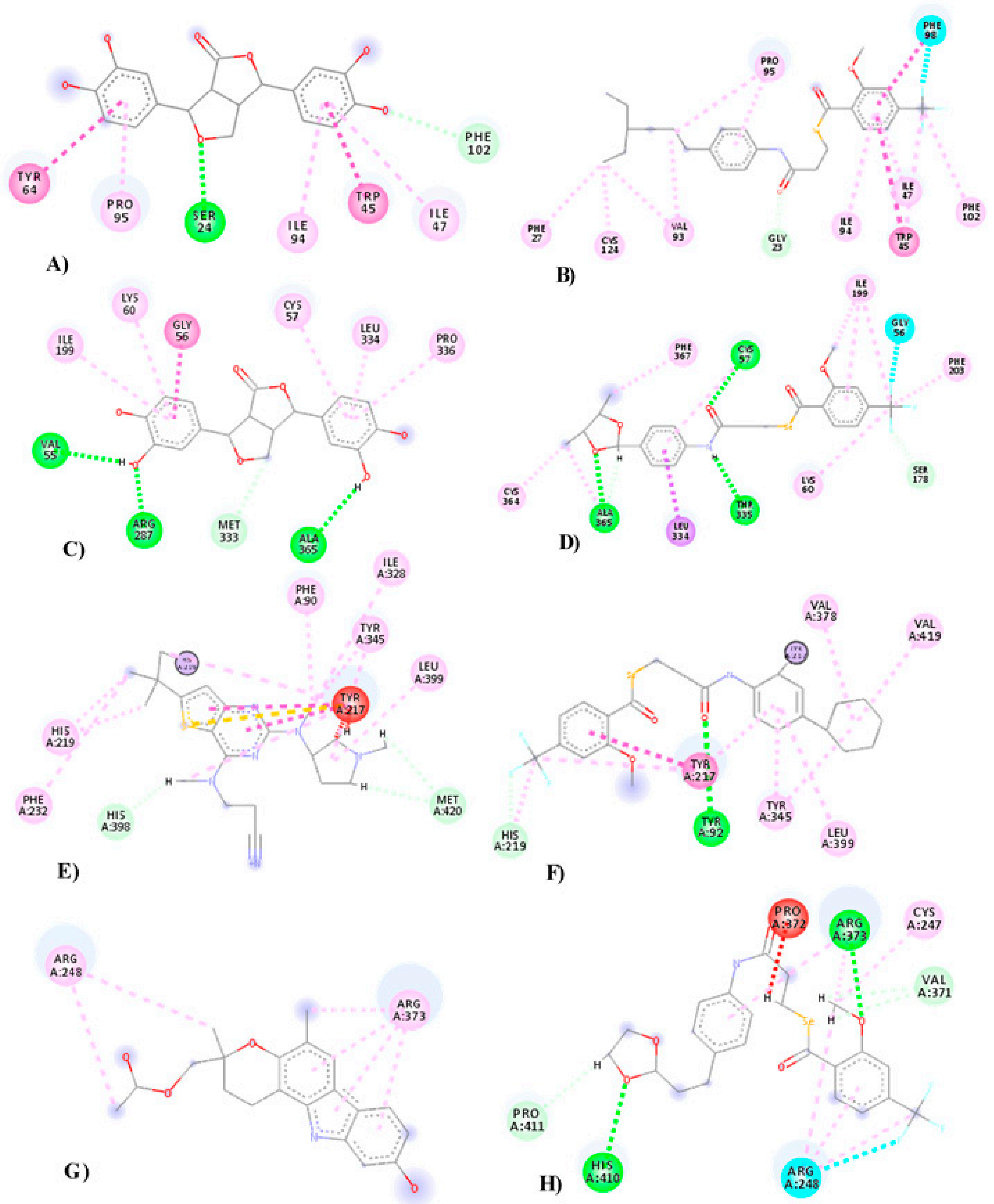

Figure S9. 2D representation of interactions with *L. major*. Legend: **A**) Glycerol 3-phosphate protein ligand; **B**) NC76 molecule; **C**) Trypanothione Reductase Ligand; **D**) NC72 molecule; **E**) N-myristoyl transferase linker (PDB: 6QDF); **F**) NC69 molecule; **G**) UDP-Glucose Pyrophosphorylase Linker (PDB: 5NZM) **H**) NC74 molecule. **Colors:** Green and Blue (Hydrogen Interactions), Pink and Lilac (Hydrophobic Interaction) and Red (Steric Interaction). **Residues:** Tyr (Tyrosine), Pro (Proline), Ser (Serine), Ile (Isoleucine), Trp (Tryptophan), Phe (Phenylalanine), Cys (Cysteine), Val (Valine), Gly (Glycine), Lys (Lysine), Leu (Leucine), Ala (Alanine), Met (Methionine), Arg (Arginine), His (Histidine).

S3. SM. Re-Docking and Validation

To validate the results of the test compound docking, the ligands and co-crystallized compounds were re-docked. The first ligand, fluconazole 2-(2,4-Difluorophenyl)-1,3-di(1H-1,2,4-Triazol-1-yl)propan-2-ol, was in complex with the crystal structure of the enzyme 14-alpha demethylase (CYP 51) to L. infantum (PDB: 3L4D). The second ligand was 6-(sec-butoxy)-2-(3-chlorophenyl)thio)pyrimidine-4-amine in complex with the crystal structure of Trypan-thione reductase for L. infantum (5EBK). For *L. major,* two ligands were used, the first being 3-[[6-~{tert}-butyl-2-[methyl-[(3~{S})-1-methylpyrrolidin-3-yl]amino]-7~{H}-thieno[3,2-d]pyrimidin-4-yl]-methyl-amino]propanenitrile, a ligand of the protein N-myristoyltransferase (PDB: 6QDF). The second ligand was Murayamine-1 complexed to the enzyme UDP-glucose-Pyrophosphorylase (PDB: 5NZM). The other proteins were obtained via homology modeling and validated via the Ramachandran plots described above.

The re-docked values of CYP 51 (PDB: 3L4D), Trypanothione Reductase (PDB: 5EBK), N-myristoyltransferase (PDB: 6QDF) and UDP-glucose-Pyrophosphorylase (PDB: 5NZM) are described in Table S5. The energy values were calculated using the MolDock Score (KJ.mol-1) and RMSD value (Root Mean Square Deviation). The RMSD value calculates the mean square distance between the ligand atoms in the crystal structure and the corresponding atoms in the anchored pose. This value is valuable in considering the prefered poses of bound ligands. For docking to be considered reliable, the RMSD value must be equal to or less than 2.0 Å. Thus, an RMSD less than 2.0 Å is widely accepted as an indication as to whether the method was successful or not [85,86].

Table 5. MolDock Score scores for binder, redocking, and RMSD achieved.

Protein	CYP 51 (PDB: 3L4D)	Trip Red (PDB: 5EBK)	*N*myristoyltransferase (PDB: 6QDF)	UDPglucosePyrofosforilase (PDB: 5NZM)
Ligand	−116.85	45.11	108.17	72.54
Redocking	−117.11	36.59	73.48	55.98
RMSD	0.25	0.20	1.23	0.23

The results obtained strongly support the reliability of the docking models, as the RMSD values obtained were no higher than 1.23 Å, and below 0.25 Å for the remaining 3 verification models. Furthermore, strongly negative values of energy were observed and further suggest strong interactions and affinity of the ligand for the protein.

Table S6. Analysis of toxicity risks for the three parameters (mutagenicity, toxic effect on the reproductive system and skin irritability).

ID	Mutagenic	Reproductive system	Skin Irritability
NC04	Low	High	None
NC05	Low	High	None
NC06	Low	High	None
NC19	Low	High	None
NC30	None	None	None
NC31	None	None	None
NC32	None	None	None
NC33	None	None	None
NC34	None	None	None
NC35	Low	High	Low
NC36	None	None	None
NC37	None	None	None
NC38	None	None	None
NC39	None	None	None
NC40	None	None	None
NC41	None	None	None

NC42	None	None	None
NC43	None	None	None
NC44	None	None	None
NC45	None	None	None
NC46	None	None	None
NC47	None	None	None
NC48	None	None	None
NC49	None	None	None
NC50	None	None	None
NC51	None	None	None
NC53	None	None	None
NC55	None	None	None
NC56	None	None	None
NC57	None	None	None
NC58	None	None	None
NC59	None	None	None
NC60	High	None	None
NC61	None	None	None
NC62	High	None	None
NC64	None	None	None
NC66	None	None	None
NC67	None	None	None
NC69	None	None	None
NC70	None	None	Low
NC73	None	None	None
NC74	None	None	None
NC76	None	None	None

Table S7. Data on the number of violations of Lipinski's rule and the rate of oral absorption.

ID	% (ABS)	Violation of Lipinski's rule
NC04	90.75	0.0
NC05	90.75	0.0
NC06	90.75	0.0
NC19	98.96	0.0
NC30	93.07	0.0
NC31	93.07	0.0
NC32	93.07	0.0
NC33	84.09	0.0
NC34	89.89	0.0
NC35	93.07	0.0
NC36	93.07	0.0
NC37	84.86	0.0
NC38	84.86	0.0
NC39	84.86	0.0
NC40	93.07	0.0
NC41	77.26	0.0
NC42	76.66	0.0
NC43	76.66	0.0
NC44	76.66	0.0
NC45	91.95	0.0
NC46	77.26	0.0
NC47	77.26	0.0
NC48	77.26	0.0
NC49	84.34	0.0
NC50	93.07	0.0
NC51	84.00	0.0

NC53	93.07	0.0
NC55	61.46	0.0
NC56	61.46	0.0
NC57	61.46	0.0
NC58	93.07	0.0
NC59	75.61	0.0
NC60	93.07	0.0
NC61	83.52	0.0
NC62	77.14	0.0
NC66	80.33	0.0
NC67	93.07	0.0
NC69	89.89	2.0
NC70	83.52	1.0
NC73	83.52	1.0
NC74	83.52	1.0
NC76	89.89	2.0

Table S8. Distribution of selected compounds with potential activity against promastigote form for the organic synthesis step.

ID	*L. braziliensis* (p%)	*L. infantum* (p%)	*L. major* (p%)	*L. amazonensis* (p%)
NC01	8	**59**	26	18
NC02	5	**59**	32	19
NC07	5	**58**	37	10
NC08	4	**61**	32	26
NC09	16	**58**	32	19
NC13	9	**52**	42	8
NC18	25	**54**	46	14
NC19	4	**60**	16	27
NC30	44	**69**	**58**	**64**
NC31	44	**68**	**56**	**61**
NC34	45	**67**	49	**72**
NC36	48	**68**	**59**	**70**
NC40	**58**	**74**	**60**	**68**
NC41	**53**	**59**	46	**61**
NC51	**61**	**68**	47	**63**
NC53	**54**	**71**	**64**	**70**

Legend: *L.b: L. braziliensis, L.i.: L. infantum, L.a.: L. amazonensis, L.m.: L. major*. Pro: Promastigote.

S4. SM. Synthesis and Characterization of 3-Chloro-N-arylpropanamide

Reactions between amines and carboxylic acid derivatives like acid halides, carboxylic acid anhydrides, and esters are common ways to prepare amides. 3-Chloro-*N*-arylapropanamides were prepared using the reaction scheme of p-substituted amines, 3-chloropropanoyl chloride (with triethylamine (Et_3N) as a base, and dichloromethane (CH_2Cl_2) as the solvent shown in Figure 14.

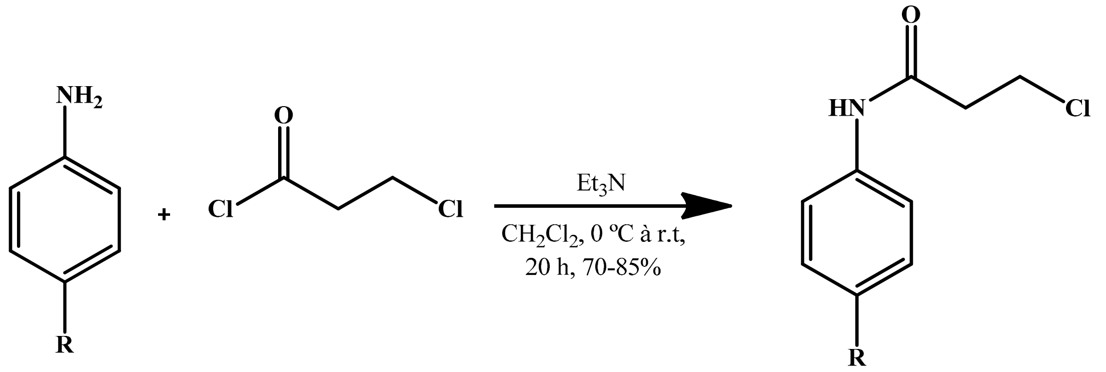

Figure S10. Reaction scheme of p-substituted amines and 3-chloropropanoyl chloride**. Subtitle:** NC01: R = H; NC02: R = CH_3; NC07: R = OCH_3; NC08: R = Cl; NC09: R = $(CH_3)_2CH$; NC13: R = NO_2; NC19: R = Br; NC18: R = $CO_2CH_2CH_3$.

The 3-chloro-*N*-arylpropanamides were purified through recrystallization using an ethanol/water solvent mixture. The structures of 3-chloro-*N*-arylpropanamides were determined by infrared, ^{1}H, and ^{13}C nuclear magnetic resonance (NMR) spectroscopic methods. In the infrared spectra of the 3-chloro-*N*-arylpropanamides, the stretches referring to the functional group (C = O) of secondary amide ranged between 1703-1651 cm^{-1}. The stretches of absorption of the N-H bonds varied between 3367-3238 cm^{-1}. The C-H sp^2 stretches of the aromatic ring ranged from 3144-3005 cm^{-1}. The C = C stretches of aromatic rings ranged from 1606–1406 cm^{-1}. The sp^3 C-H stretches ranged from 2983–2862 cm^{-1}. For the compound containing the nitro group (NC13) there were two absorption bands, one asymmetric and the other symmetric of the N = O bond around 1500 and 1319 cm^{-1}, respectively.

In the ^{1}H and ^{13}C nuclear magnetic resonance (NMR) spectra analyses of all 3-chloro-*N*-arylapropanamides, we can observe three characteristic signals that confirm the formation of the compounds. In the ^{1}H NMR spectra of all the compounds obtained, we can observe three general signals: a triplet for two hydrogens attributed to the methylene protons of (H-3) in the range of 3.89-3.85 ppm with a coupling constant (*J*) ranging between 6.5–6.2 Hz, one triplet for two hydrogens assigned to the methylene protons of (H-2) in the range of 2.90–2.77 ppm with a coupling constant (*J*) ranging between 6.5–6.2 Hz, and a broad band assigned to the protons of the amide NH in the region of 10.68–7.39 ppm.

For the ^{13}C NMR spectrum, we can observe C = O signals from the amide (C-1) between 169.12–167.70 ppm, a signal at 40.16–39.43 ppm referring to methylene carbon (C-2) and a signal between 40.67–40.45 ppm referring to the methylene carbon (C-3).

S5. SM. Synthesis and Characterization of Selene-Ethylenelactamides

Given the broad spectrum of biological activities demonstrated by organo-selenium compounds and the *in silico* screening, eight selenoethylenelactamides were synthesized (Figure S11).

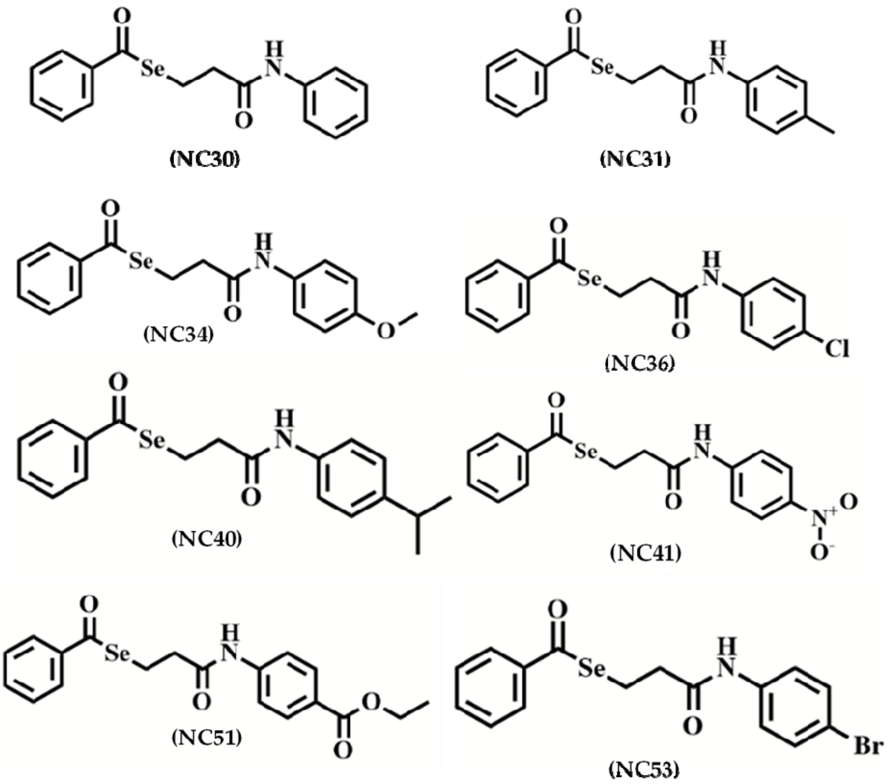

Figure S11. Structures of selected seleno-ethylenelactamides.

Souza et al (2019) reported the synthesis of selenoglycolicamides using the "one pot" process by reacting sodium benzoselenoate with 2-chloro-*N*-arylacetamides and a water solvent at room temperature. The preparation of selenoethylenelactamides was performed according to the methods described by Souza et al (2019) with small modifications in the last step, where the reaction is heated to a temperature of 60 °C overnight. The synthesis scheme can be seen in Figure S12.

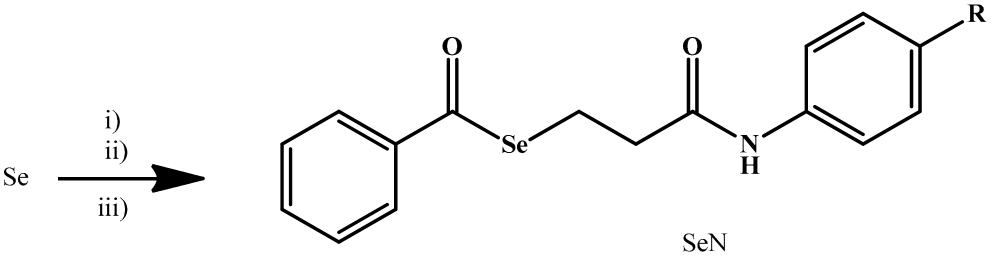

Figure S12. Synthesis of target seleno-ethylenelactamides. Subtitle: Reagents and conditions: i) $NaBH_4$, H_2O, t.a, 10 min; ii) benzoyl chloride, t.a, 30 min; iii) 3-chloro-*N*-arylpropanamide, 60 °C, overnight. NC30: R = H; NC31: R = CH_3; NC34: R = OCH_3; NC36: R = Cl; NC40: R = $(CH_3)_2CH$; NC41: R = NO_2; NC51: R = $CO_2CH_2CH_3$; NC53: R = Br.

The synthesis process was planned in three stages: i) the production of sodium hydrogen selenide by the reaction of selenium (black powder) with sodium borohydride in an aqueous solution; ii) NaHSe reacts with benzoyl chloride to form sodium benzoseleneate; iii) Selenol ester derivatives are produced by the reaction of sodium benzoseleneate with 3-chloro-*N*-arylpropanamides. Compounds were purified by recrystallization using an ethanol solvent.

S6. SM. Interpretation of ^{1}H and ^{13}C NMR Spectra of Seleno-ethylenelactamides

The seleno-ethylenelactamides were confirmed through ^{1}H and ^{13}C NMR, since these compounds have very characteristic signals. In the ^{1}H NMR spectra three characteristic signals can be observed: one in the region 3.38–3.27 ppm attributed to the CH_2 methylene protons of (H-6) with a coupling constant (*J*) ranging between 6.9–6.8 Hz; one in the region 2.95–2.82 ppm attributed to the CH_2 methylene protons of (H-7) with a coupling constant (*J*) ranging between 6.9-6.8 Hz and one attributed to the N-H proton in the region 10.61–7.43 ppm. In the ^{13}C spectra, four characteristic signals can be observed that the compounds obtained are present: a C = O signal between 170.79–169.56 ppm referring to amide carbonyl (C-8), a C = O signal between 195.63–194.36 ppm referring to selenolester carbonyl (C-1), a signal between 38.50–36.78 ppm corresponding to CH_2 methylene carbon (C-7), and a signal between 20.81–19.80 ppm corresponding to CH_2 methylene carbon (C-6).

S7. SM. Interpretation of Infrared (IR) spectra of seleno-ethylenelactamides

The infrared spectra of seleno-ethylenelactamides display bands characteristic of these molecules, including a C = O stretch from the selenol ester, C = O stretch of the secondary amide, the NH stretch from a secondary amide, C = C stretching of the aromatic ring, and bands of vibrations of other groups such as halogens and the NO_2 bound to the benzene ring.

In the studied spectra, the absorption patterns of the groups discussed above were evident and provided for a simple analysis. The stretch absorption bands referring to the functional group (C = O) of the secondary amide ranged from 1651-1697 cm^{-1}. The stretch absorption bands referring to the C = O of selenol esters ranged from 1689 to 1643 cm^{-1}. The absorption bands of the N-H stretch from the secondary amides ranged from 3360-3264 cm^{-1}. Absorptions of sp^2 C-H stretches in the aromatic rings ranged from 3116-3022 cm^{-1}. Absorptions of sp^3 C-H stretches ranged between 2999–2887 cm^{-1}. Lastly, absorptions of C = C stretches of the aromatic rings ranged from 1595–1402 cm^{-1}.

For compounds containing halogens in the para position of the aromatic ring (NC36 and NC53) the stretch absorptions were observed at 1087 and 1070 cm^{-1}, respectively. NC41 contains a nitro group, seen as two stretch absorption bands, one asymmetric and the other symmetric of the N = O bond around 1504 and 1334 cm^{-1}, respectively. For full spectral information, see the supplemental material.

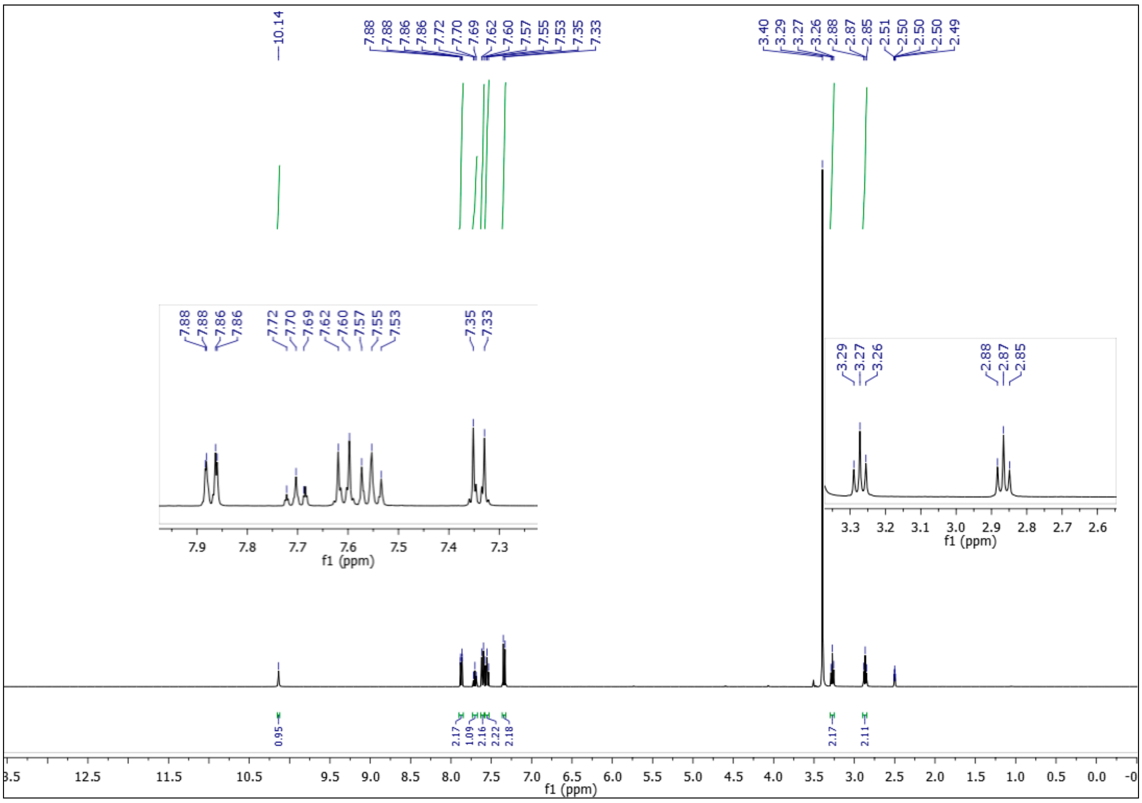

Figure S13. Hydrogen spectrum (400 MHz DMSO-d6) of N-(4-chlorophenyl)benzoselenoethylenelacticamide NC36.

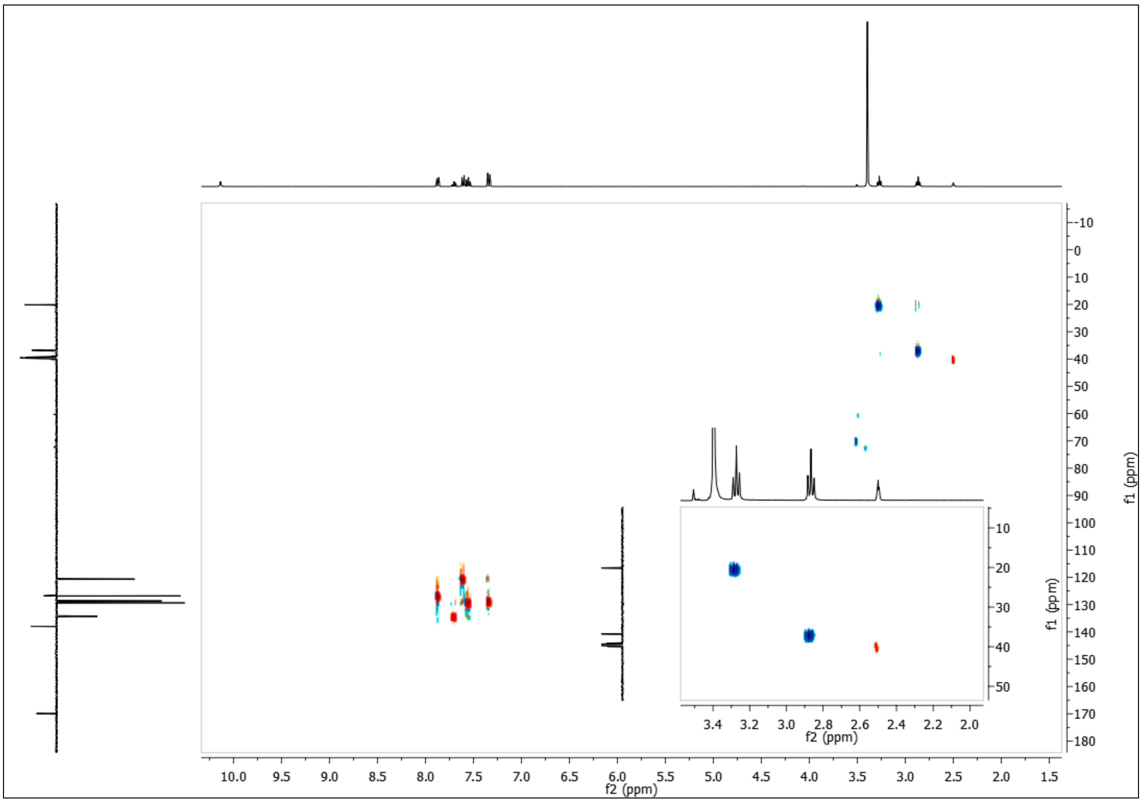

Figure S14. 1H,13C-HSQC NMR spectrum (400 MHz, CDCl3) of N-(4-chlorophenyl)benzoselenoethylenelactamide (NC36).

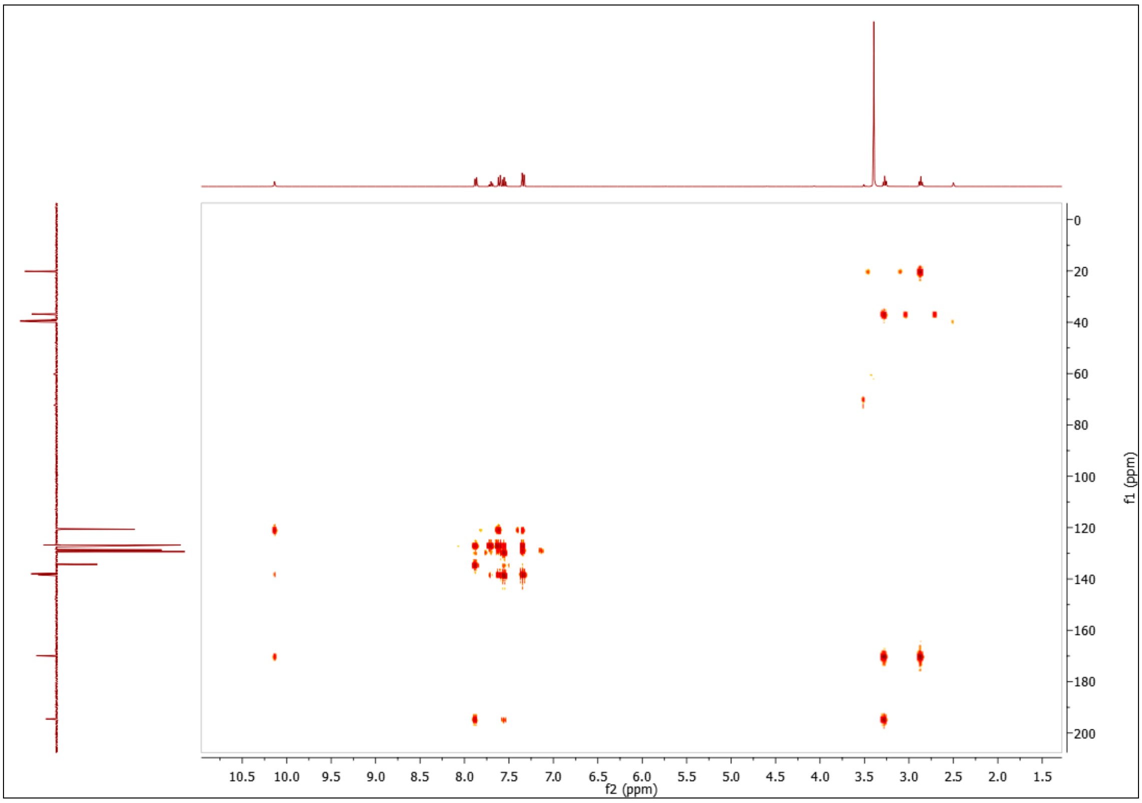

Figure S15. 1H,13C-HMBC NMR spectrum (400 MHz, DMSO-d6) of N-(4-chlorophenyl)benzoselenoethylenelactamide (NC36).

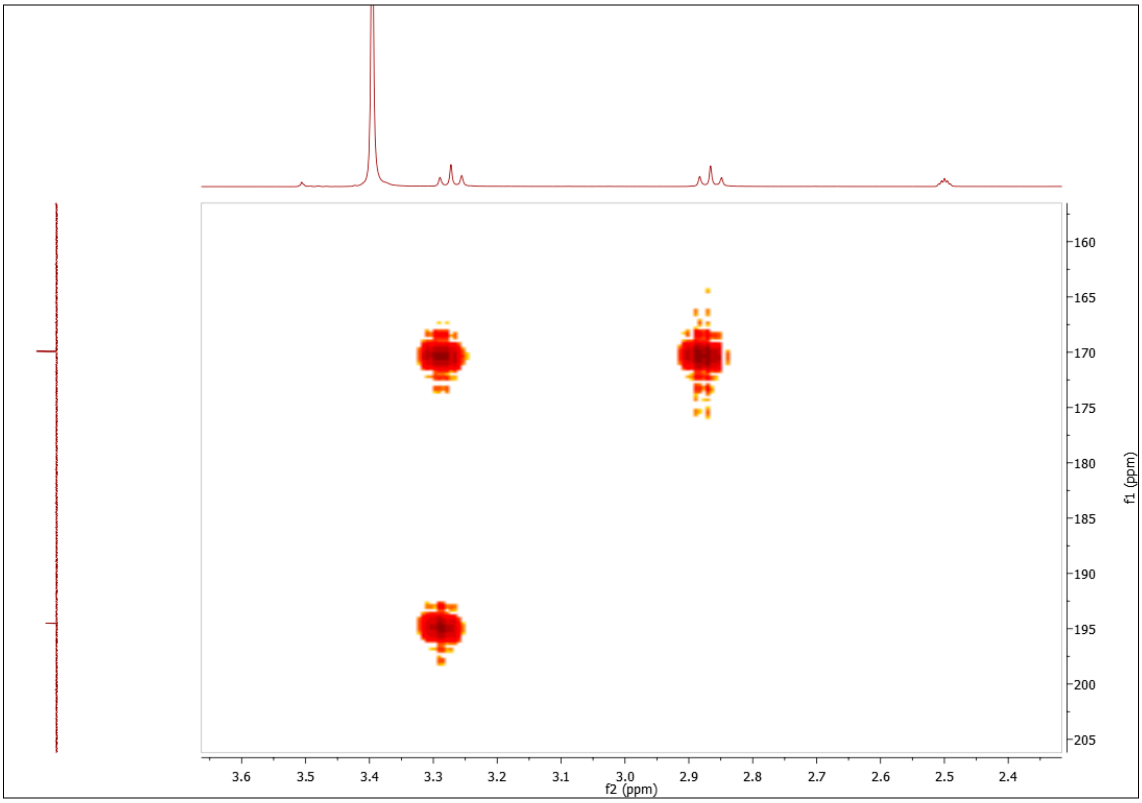

Figure S16. Expansion 1H,13C-HMBC NMR spectrum (400 MHz, DMSO-d6) of N-(4-chlorophenyl)benzoseleneethylenelactamide (NC36).

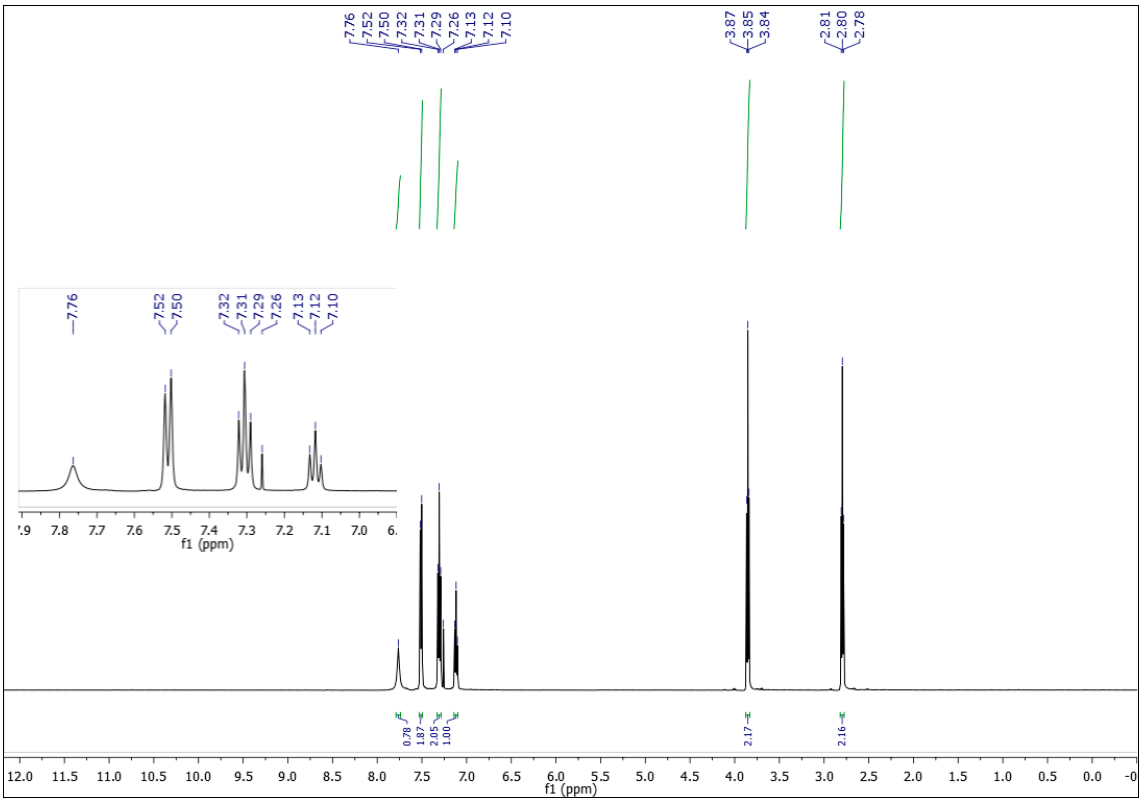

Figure S17. 1H NMR spectrum (500 MHz, CDCl 3) of 3-chloro-N-phenylpropanamide (NC01).

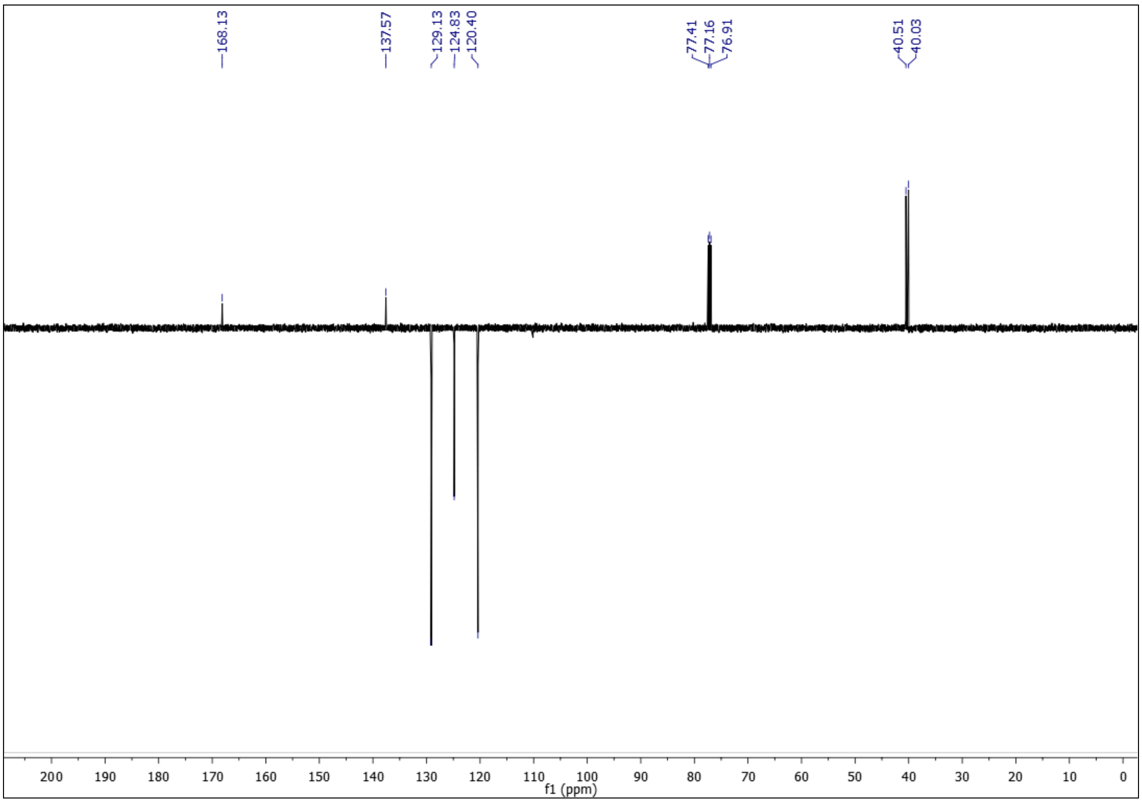

Figure S18. 13 C NMR spectrum (126 MHz, CDCl 3) of 3-chloro-N-phenylpropanamide (NC01).

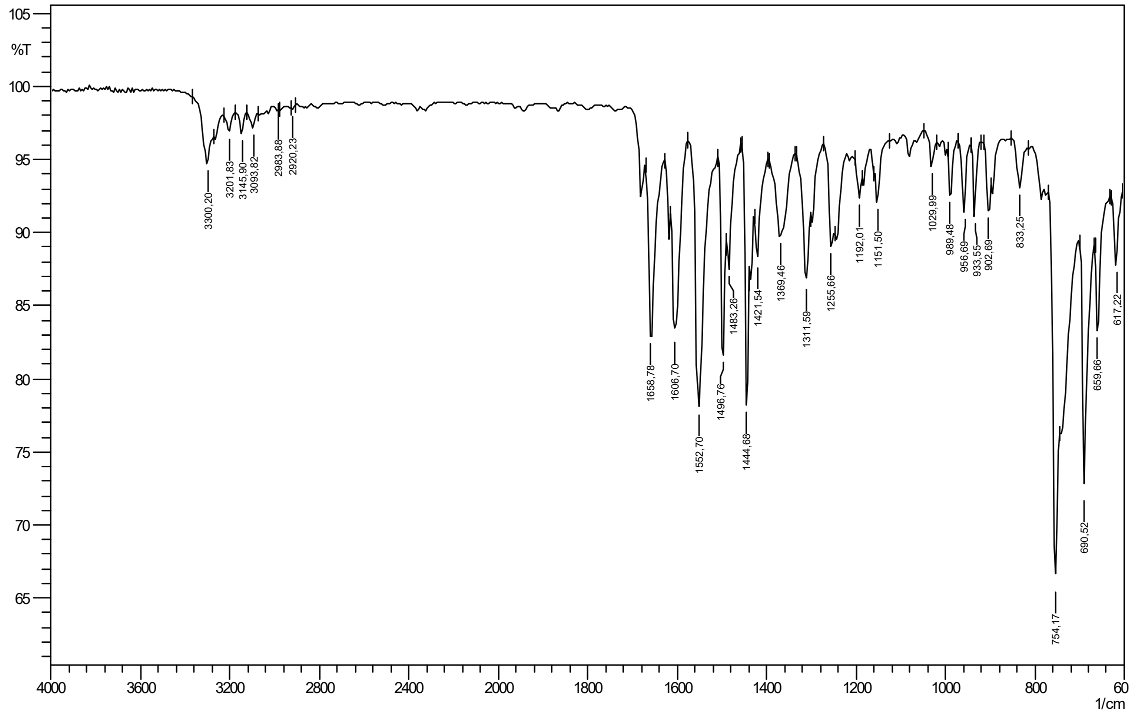

Figure S19. Infrared spectrum (ATR) of 3-chloro-N-phenylpropanamide (NC01).

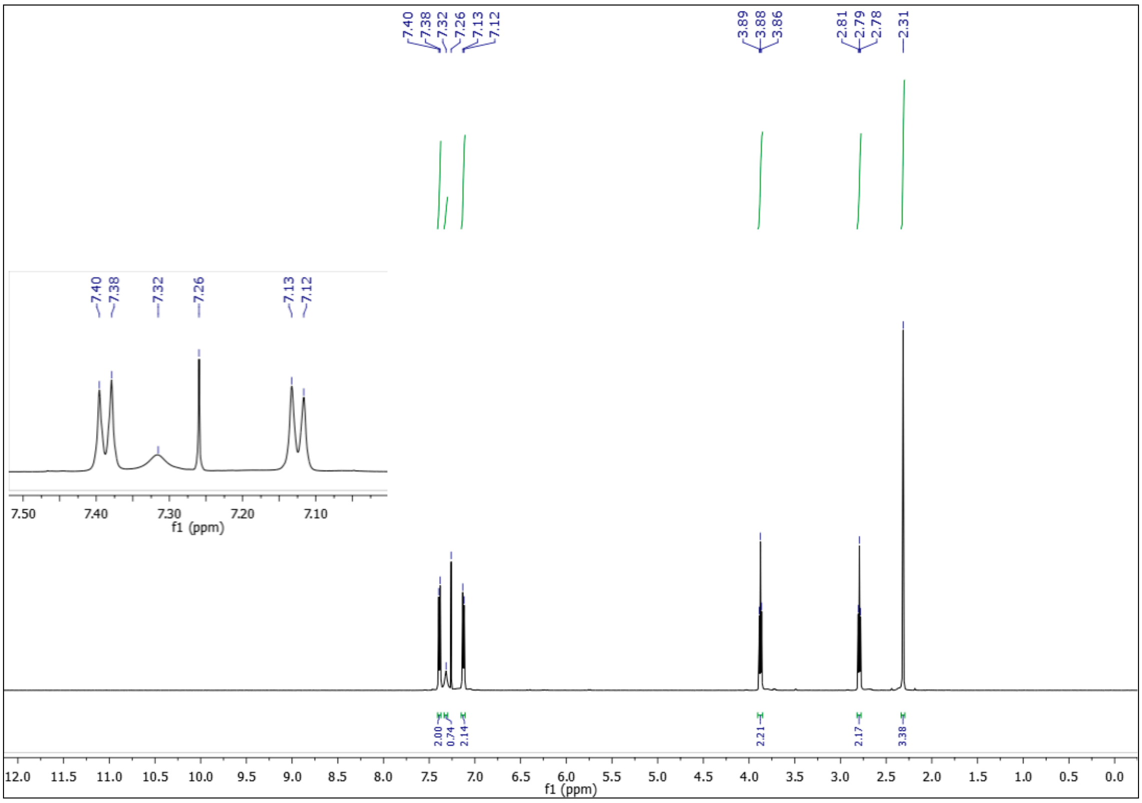

Figure S20. 1H NMR spectrum (500 MHz, CDCl 3) of 3-chloro-N-(4-methylphenyl)propanamide (NC02).

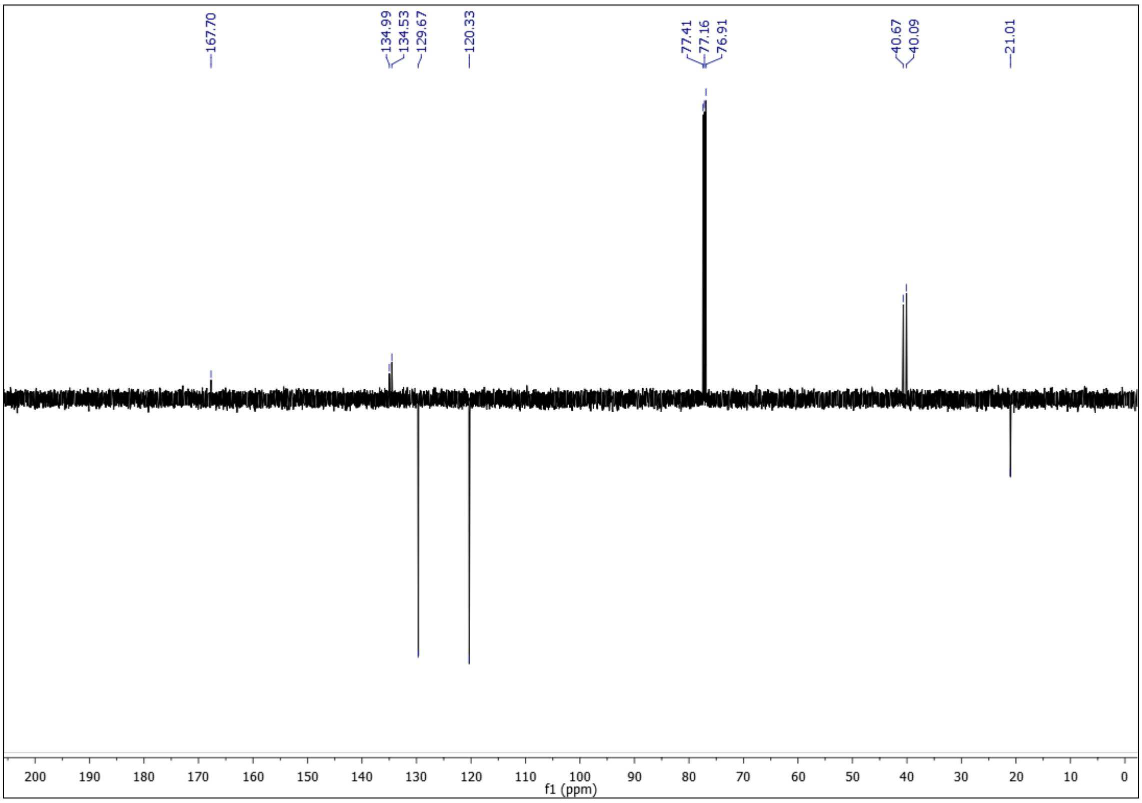

Figure S21. 1H NMR spectrum (500 MHz, CDCl 3) of 3-chloro-N-(4-methylphenyl) propanamide (NC02).

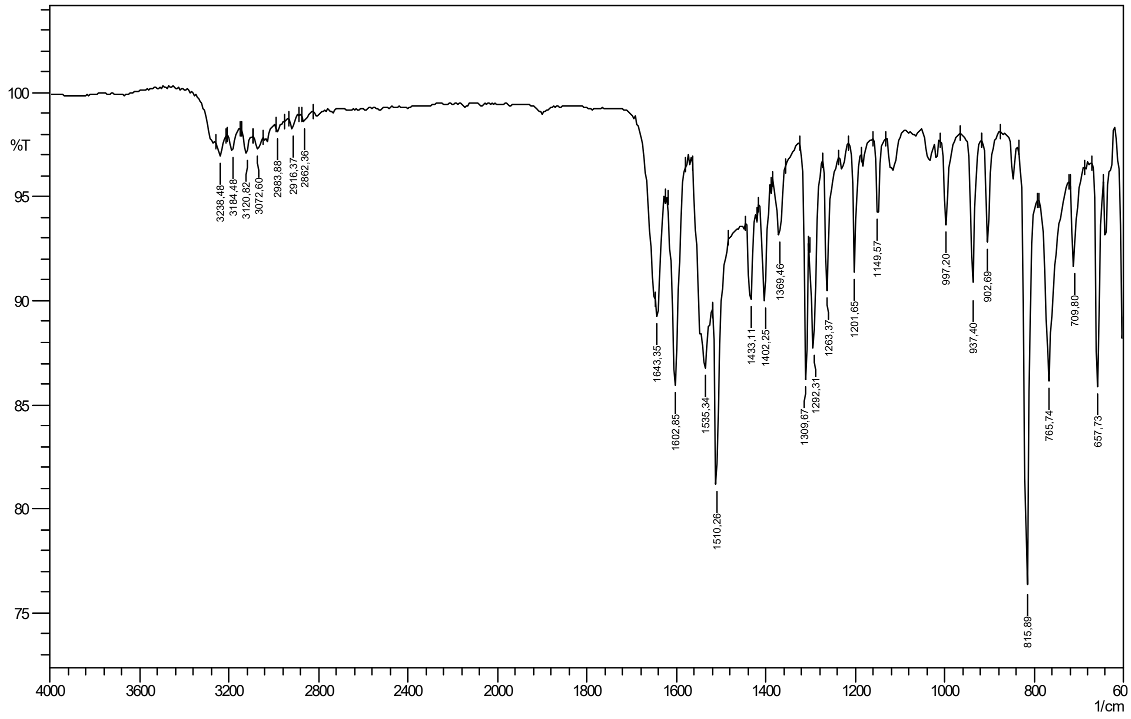

Figure S22. Infrared spectrum (ATR) of 3-chloro-N-(4-methylphenyl)propanamide (NC02).

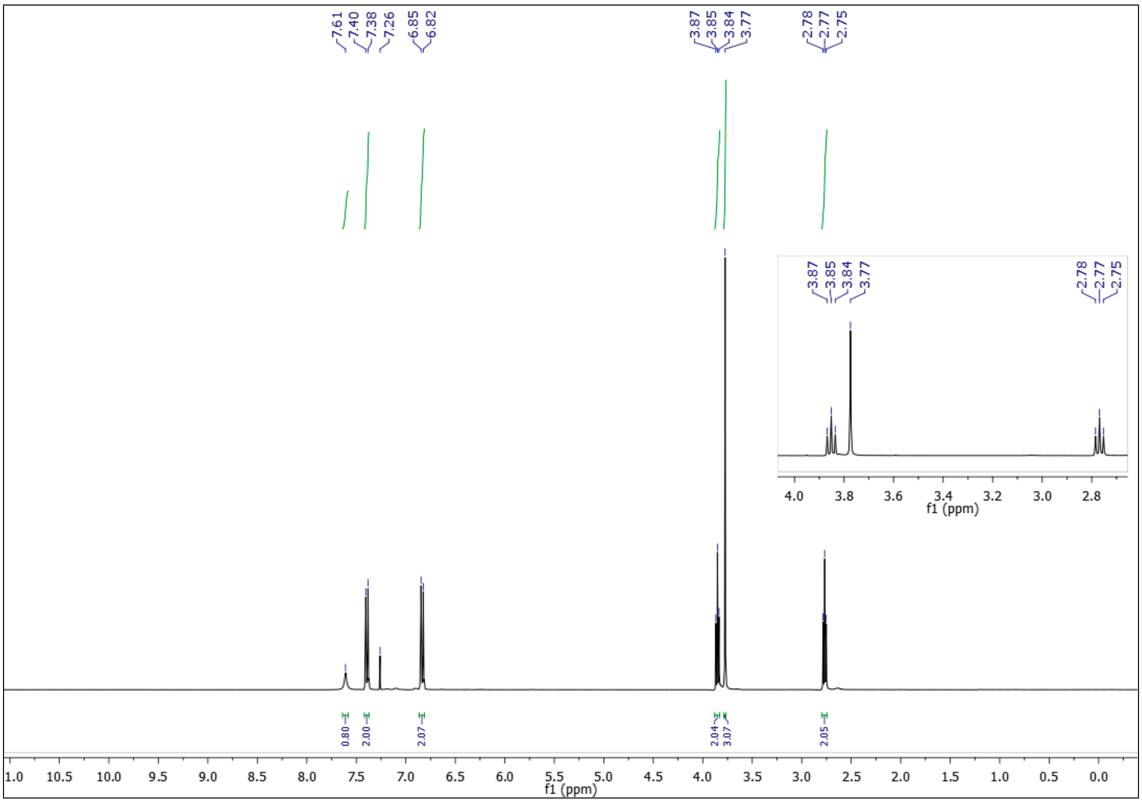

Figure S23. 1H NMR spectrum (400 MHz, CDCl3) of 3-chloro-N-(4-methoxyphenyl)propanamide (NC07).

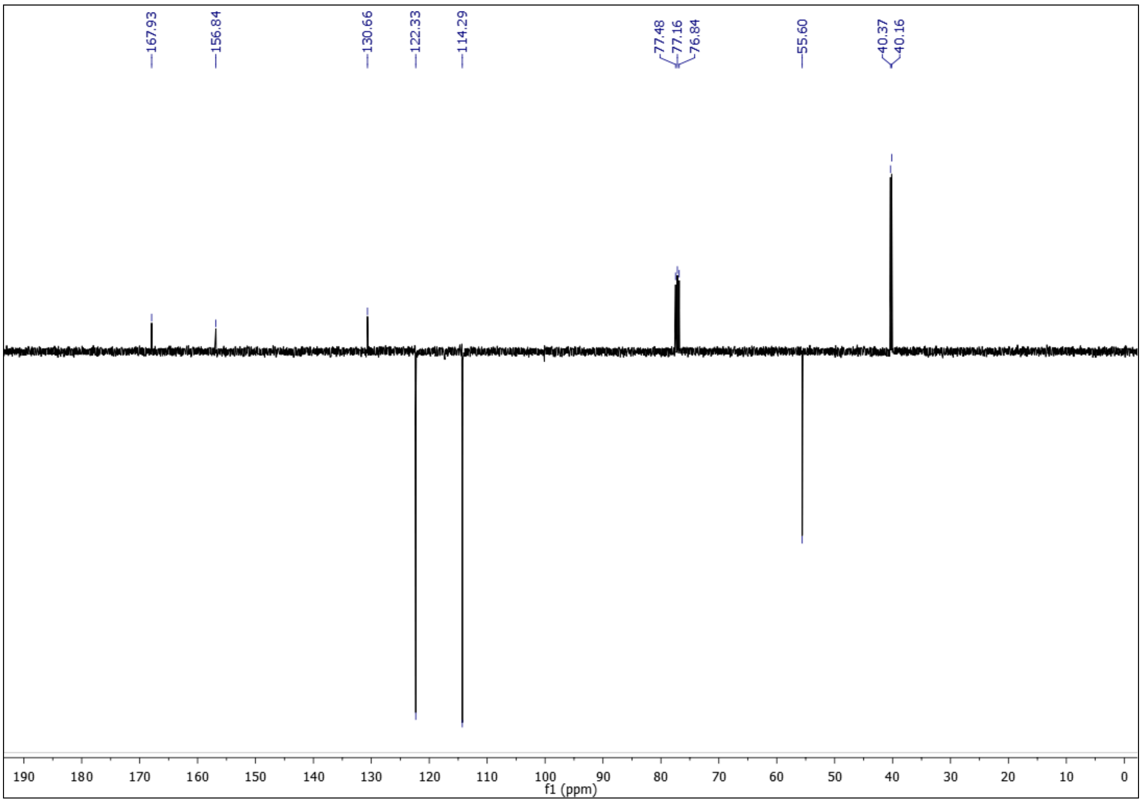

Figure S24. 13 C NMR spectrum (101 MHz, CDCl 3) of 3-chloro-N-(4-methoxyphenyl)propanamide (NC07).

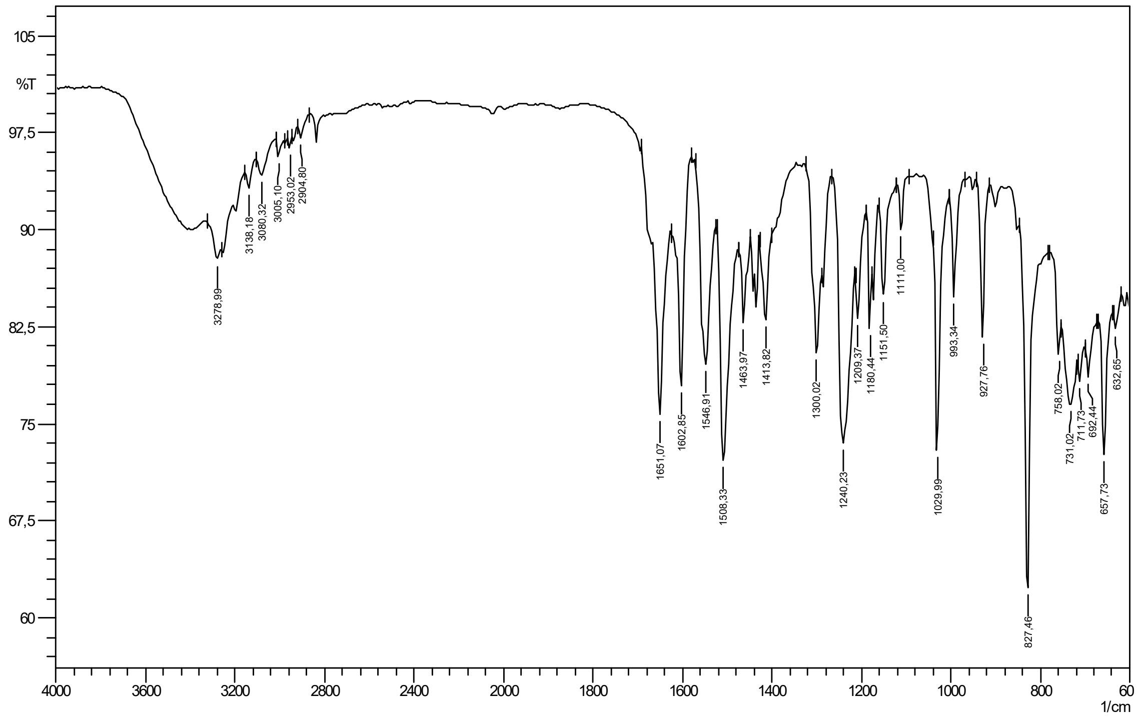

Figure S25. Infrared spectrum (ATR) of 3-chloro-N-(4-methoxyphenyl)propanamide (NC07).

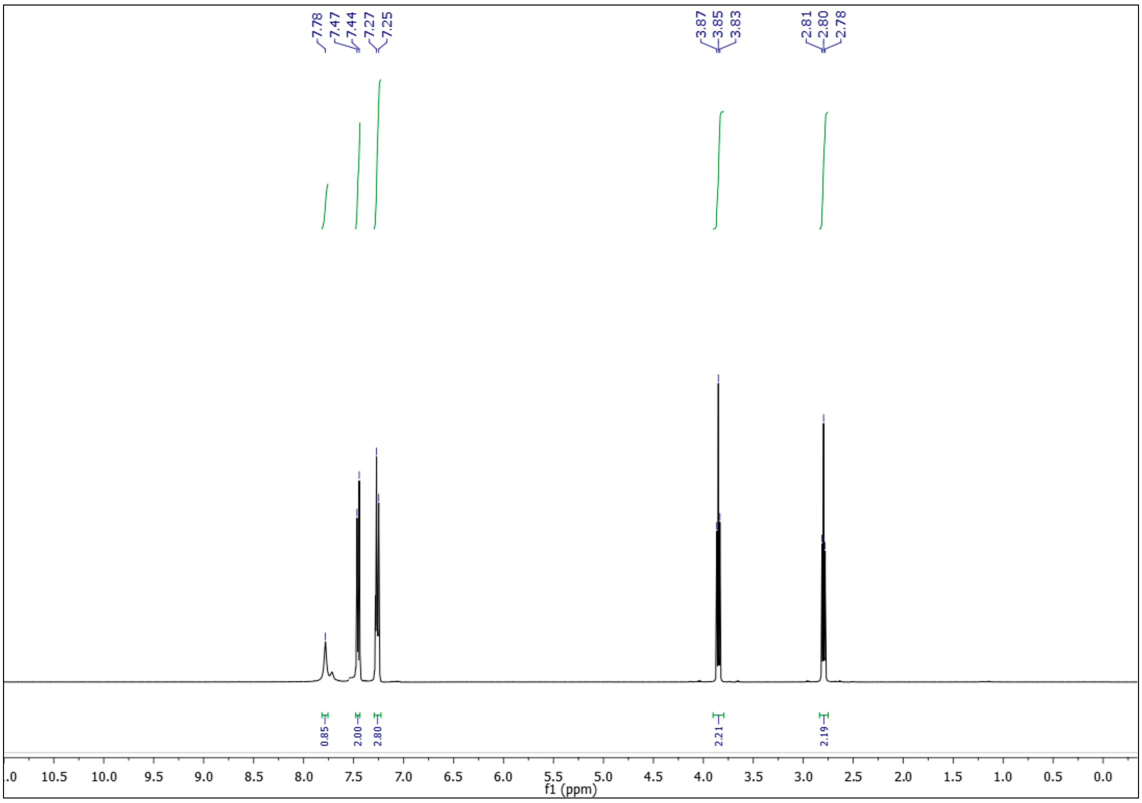

Figure S26. 1H NMR spectrum (400 MHz, CDCl 3) of 3-chloro-N-(4-chlorophenyl)propanamide (NC08) (Chlorine)**.**

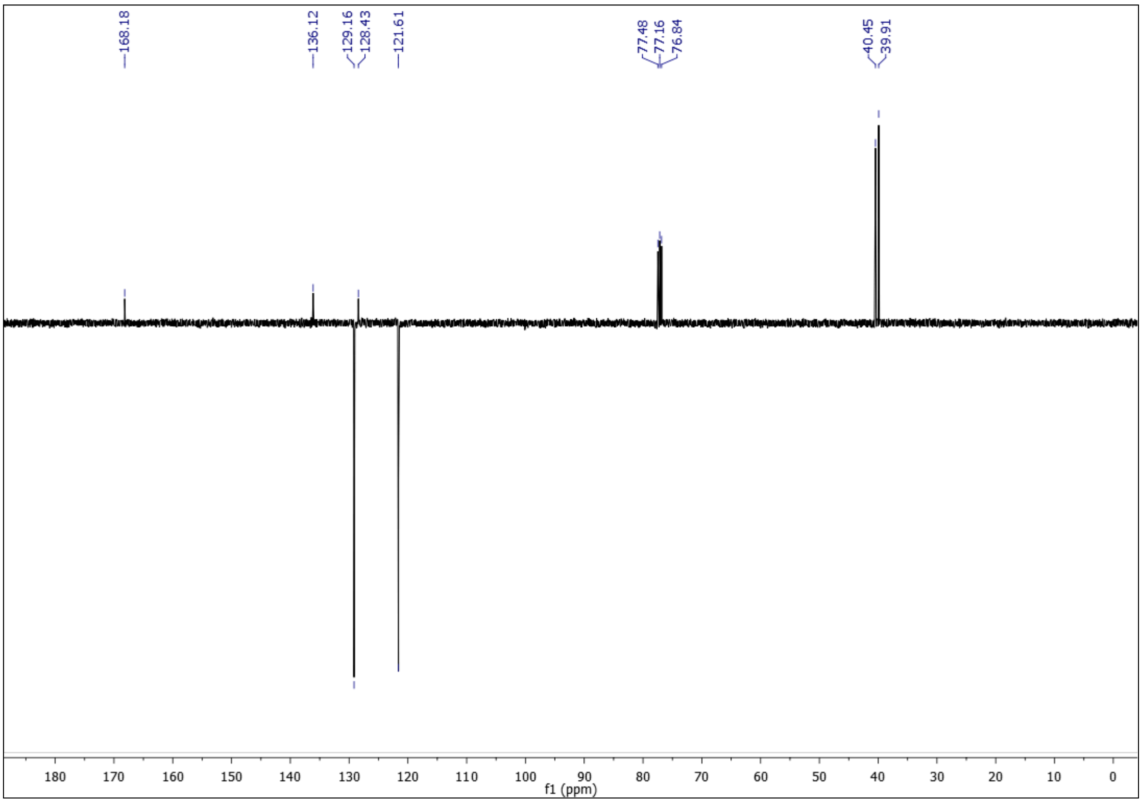

Figure S27. 13 C NMR spectrum (101 MHz, CDCl 3) of 3-chloro-N-(4-chlorophenyl)propanamide (NC08).

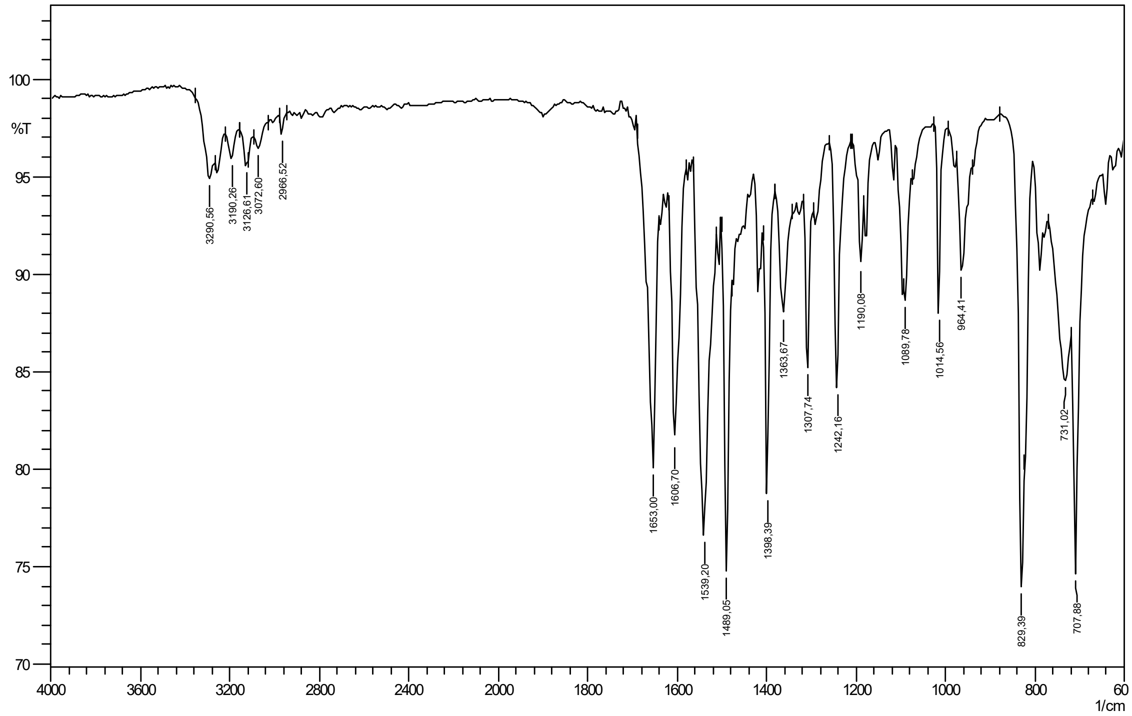

Figure S28. Infrared spectrum (ATR) of 3-chloro-N-(4-chlorophenyl)propanamide (NC08).

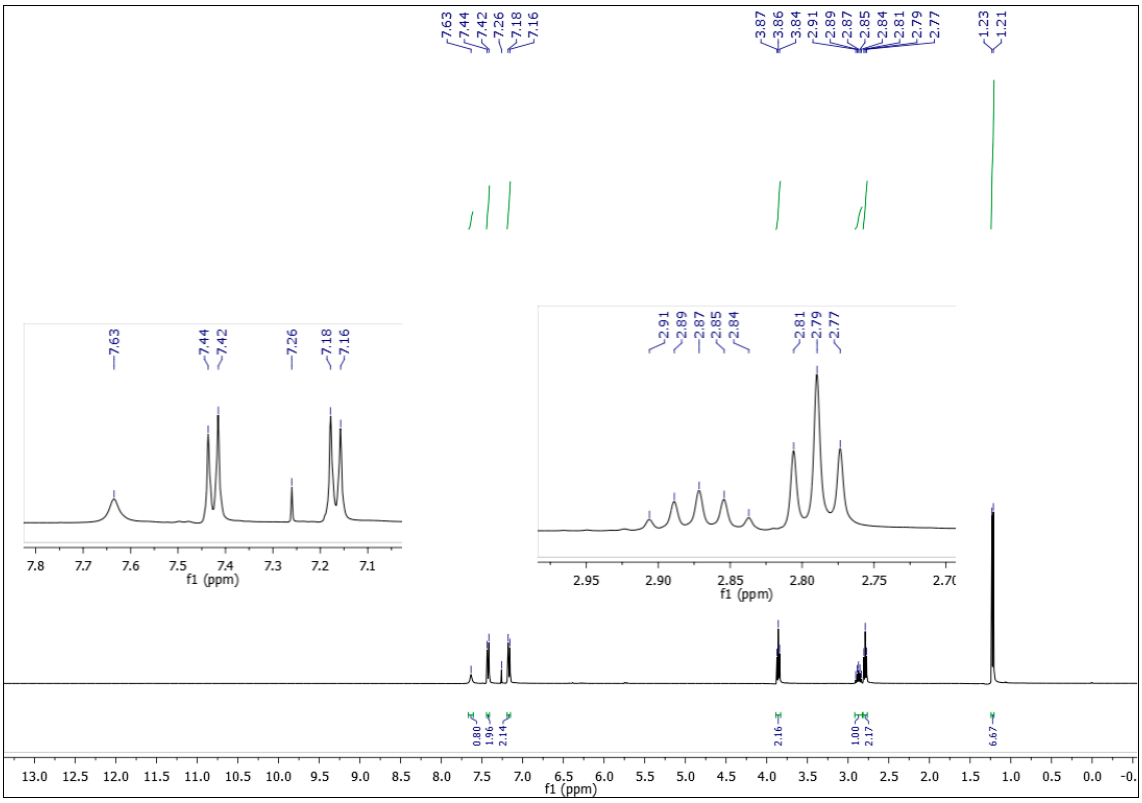

Figure S29. 1H NMR spectrum (400 MHz, CDCl3) of 3-chloro-N-(4-isopropylphenyl) propanamide NC09.

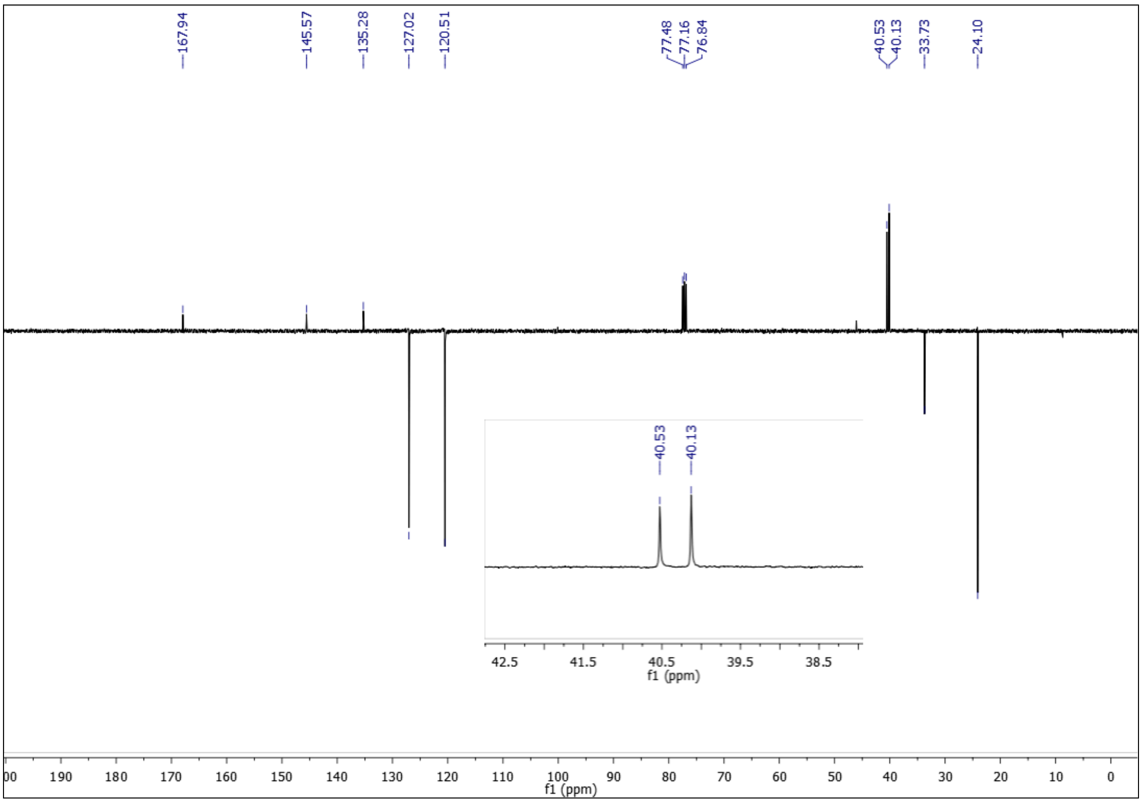

Figure S30. 13 C NMR spectrum (101 MHz, CDCl 3) of 3-chloro-N-(4-isopropylphenyl)propanamide (NC09).

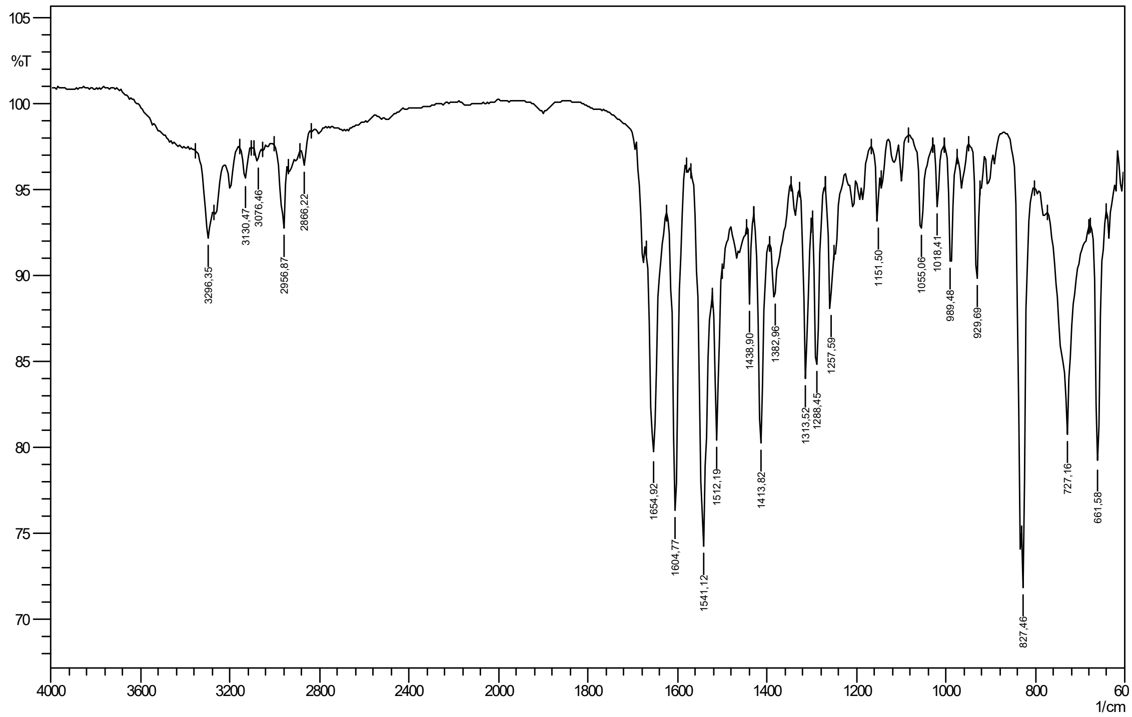

Figure S31. Infrared spectrum (ATR) of 3-chloro-N-(4-isopropylphenyl)propanamide (NC09).

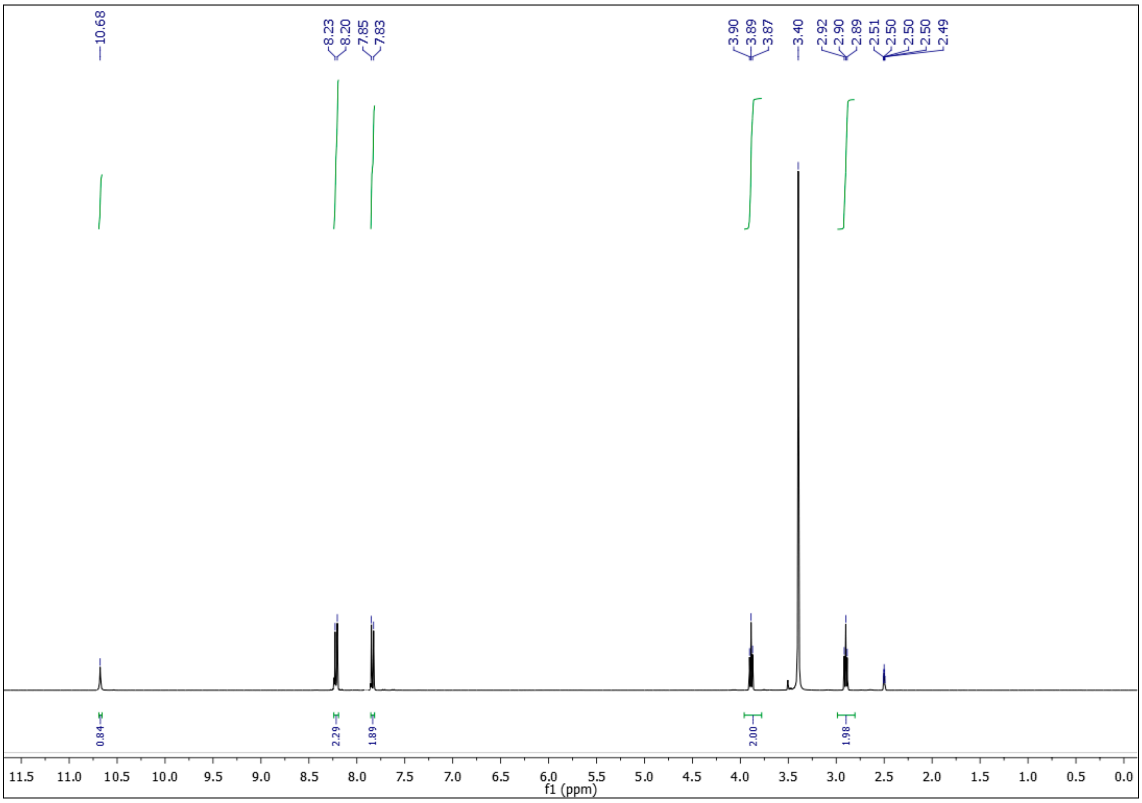

Figure S32. 1H NMR spectrum (400 MHz, DMSO-d6) of 3-chloro-N-(4-nitrophenyl)propanamide (NC13).

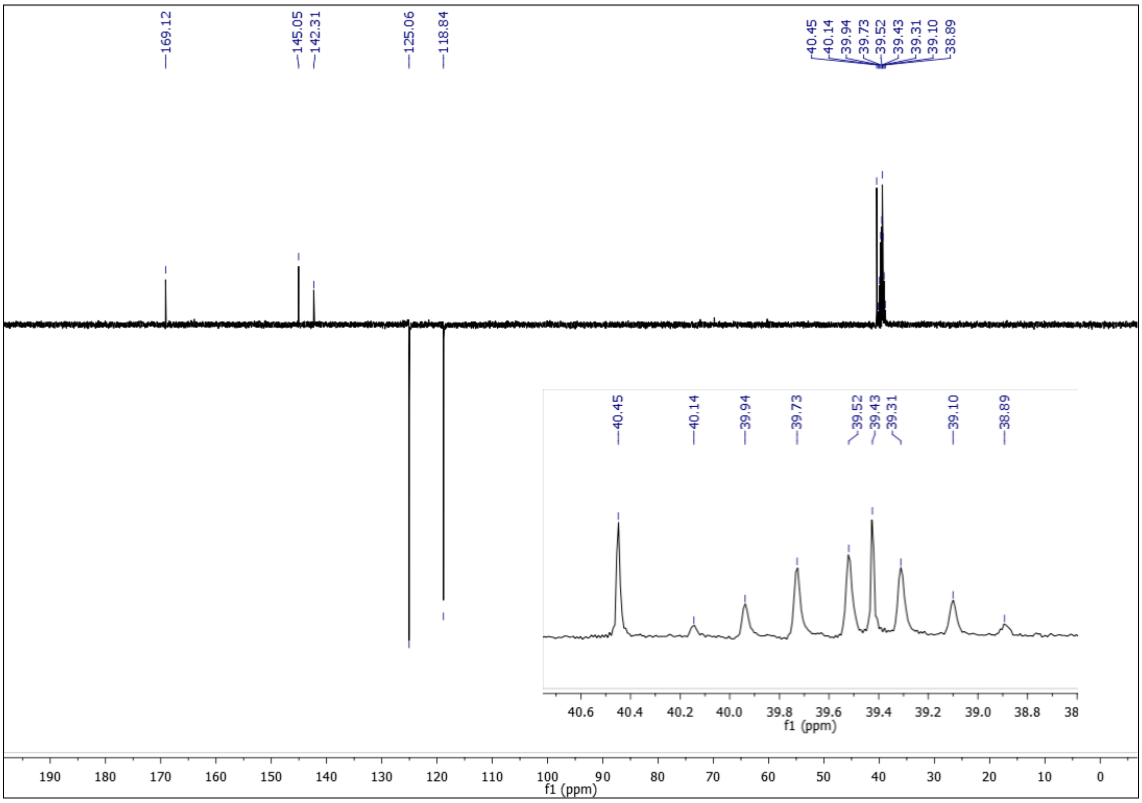

Figure S33. 13 C NMR spectrum (101 MHz, DMSO-d6) of 3-chloro-N-(4-nitrophenyl)propanamide (NC13).

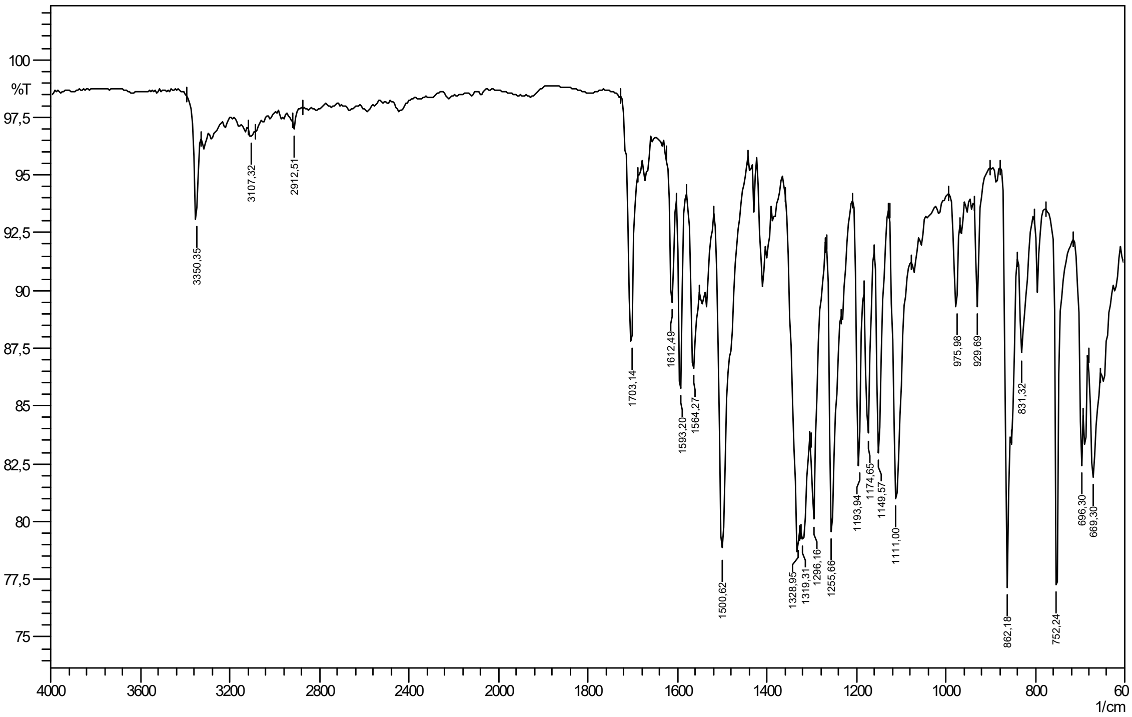

Figure S34. Infrared spectrum (ATR) of 3-chloro-N-(4-nitrophenyl)propanamide (NC13).

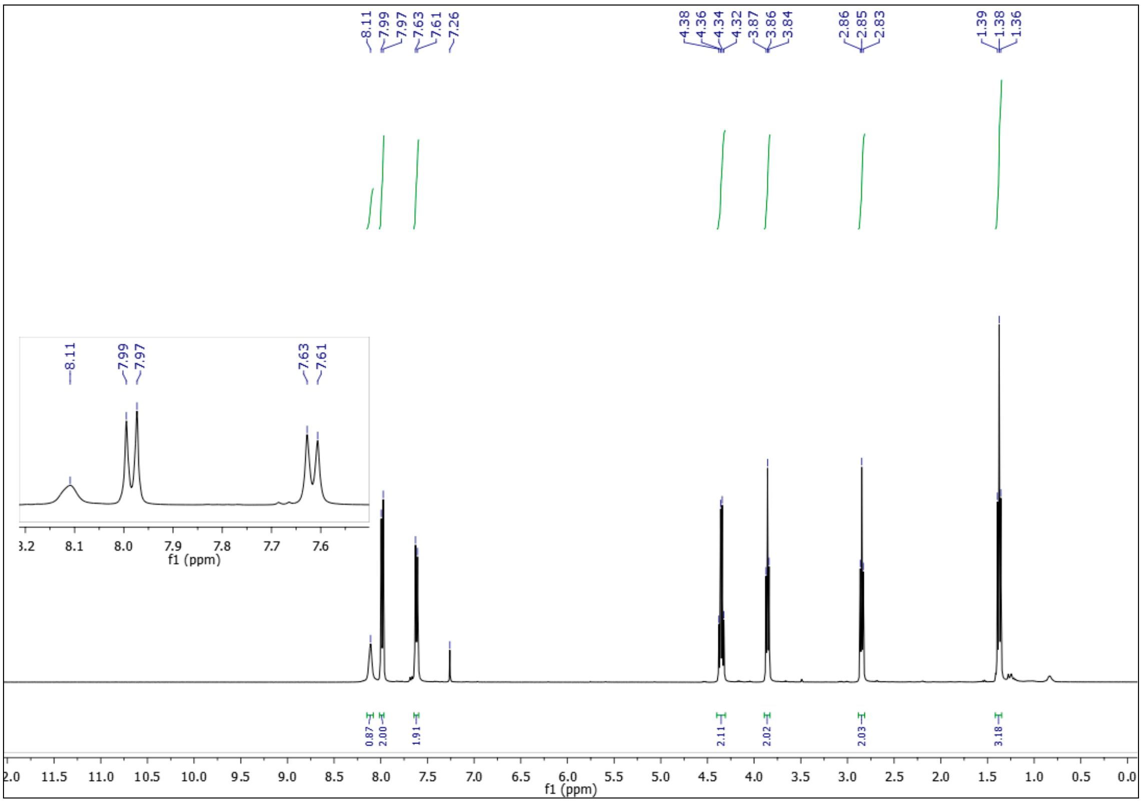

Figure S35. Hydrogen Spectrum (400 MHz, CDCl3) of Ethyl 4-(3-chloropropanamide) Benzoate (NC18).

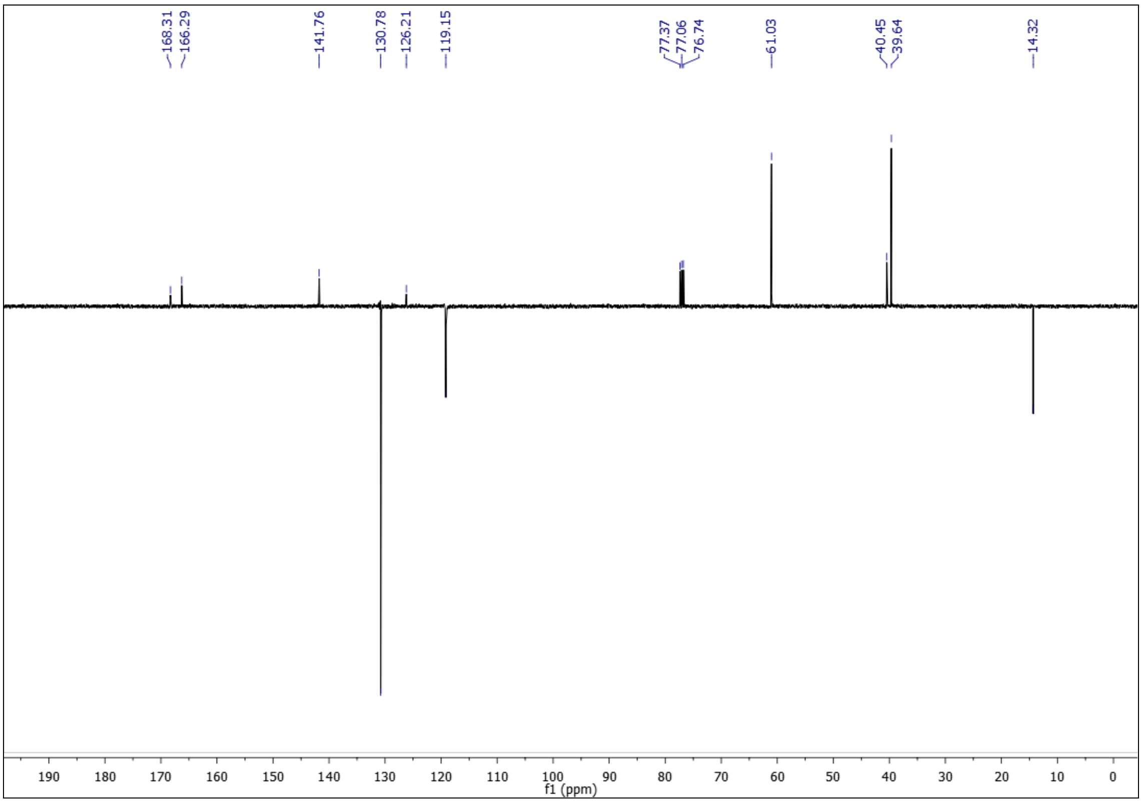

Figure S36. Carbon Spectrum (101 MHz, $CDCl_3$) of Ethyl 4-(3-chloropropanamide) Benzoate (NC18).

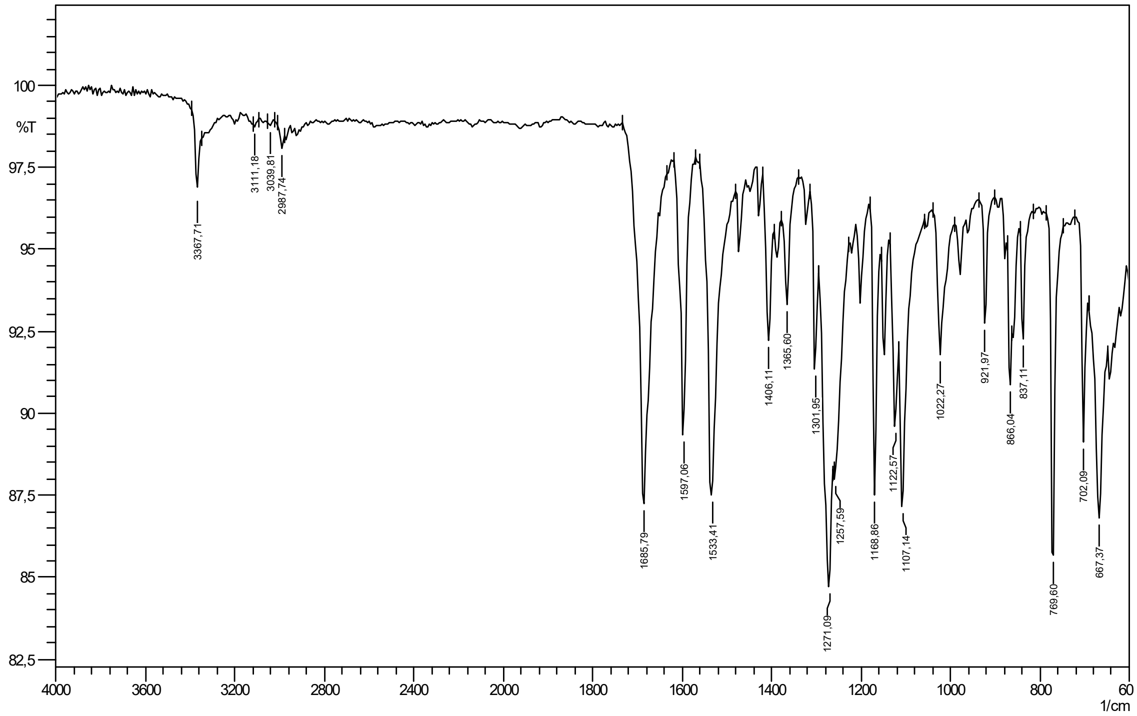

Figure S37. Infrared Spectrum (ATR) of Ethyl 4-(3-Chloropropanamide) Benzoate (NC18).

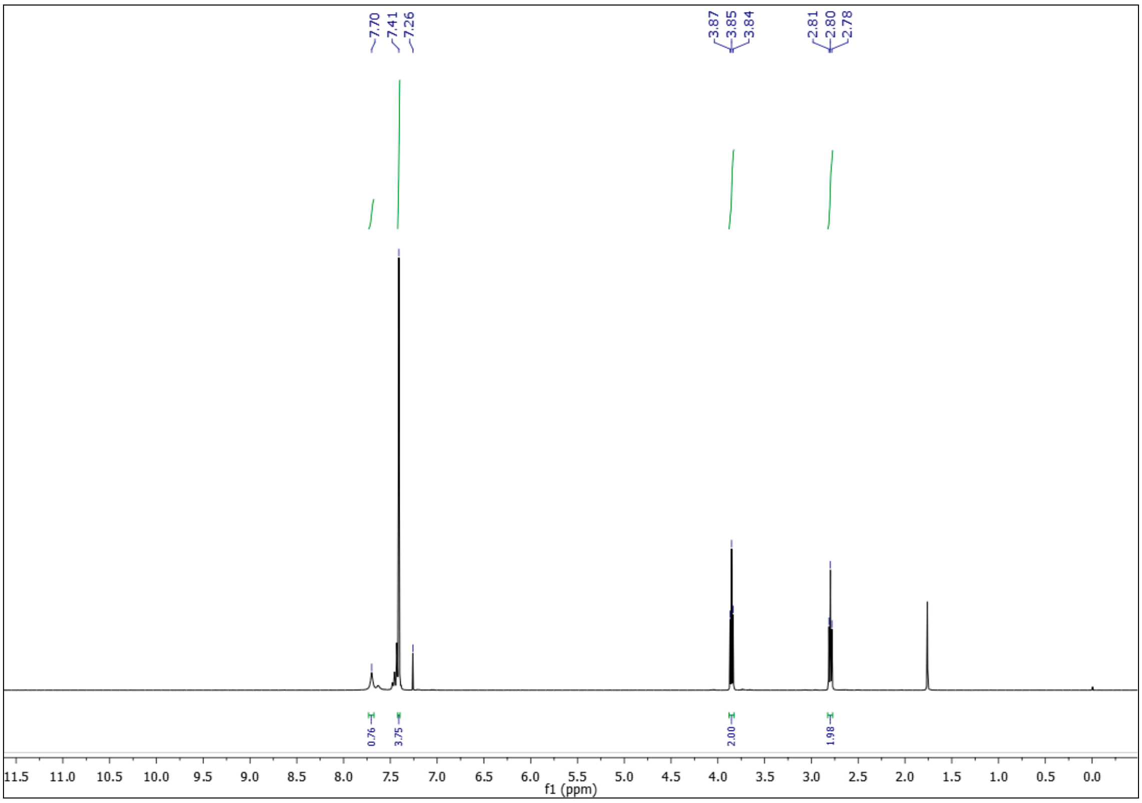

Figure S38. 1H NMR spectrum (400 MHz, CDCl 3) of 3-chloro-N-(4-bromophenyl) propanamide (NC19) (Bromine).

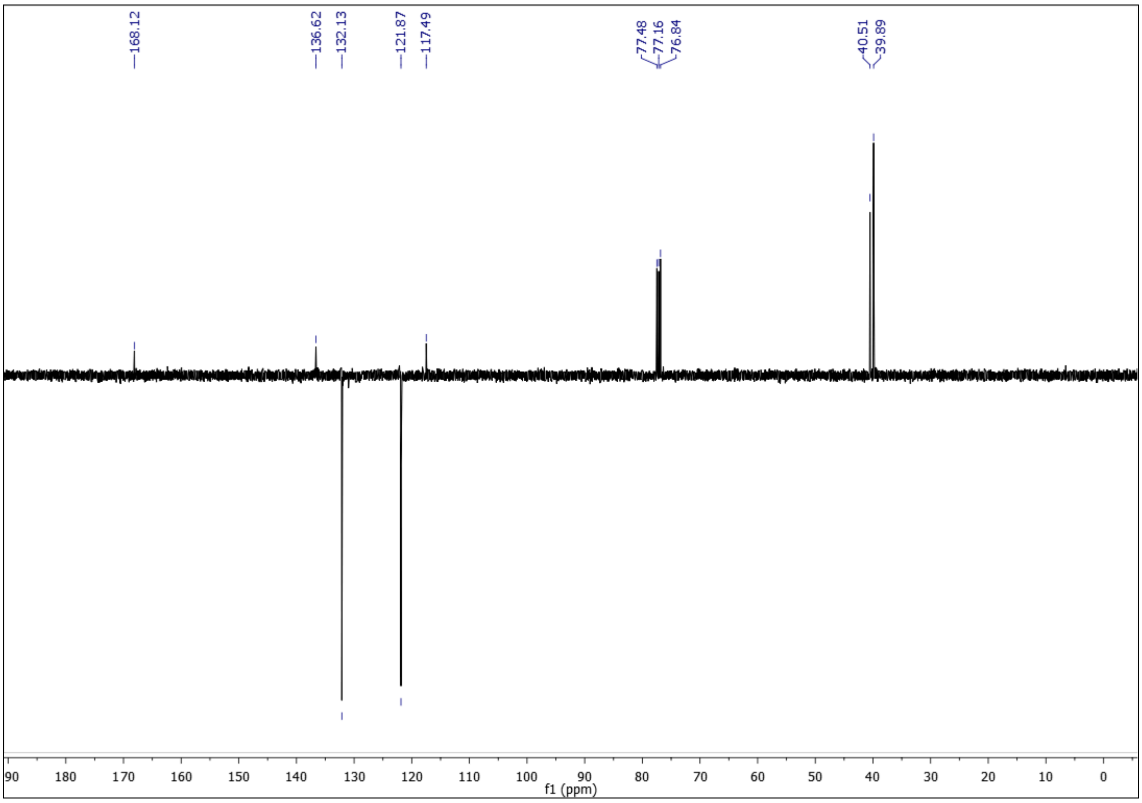

Figure S39. 13 C NMR spectrum (101 MHz, $CDCl_3$) of 3-chloro-N-(4-bromophenyl)propanamide (NC19) (Bromine).

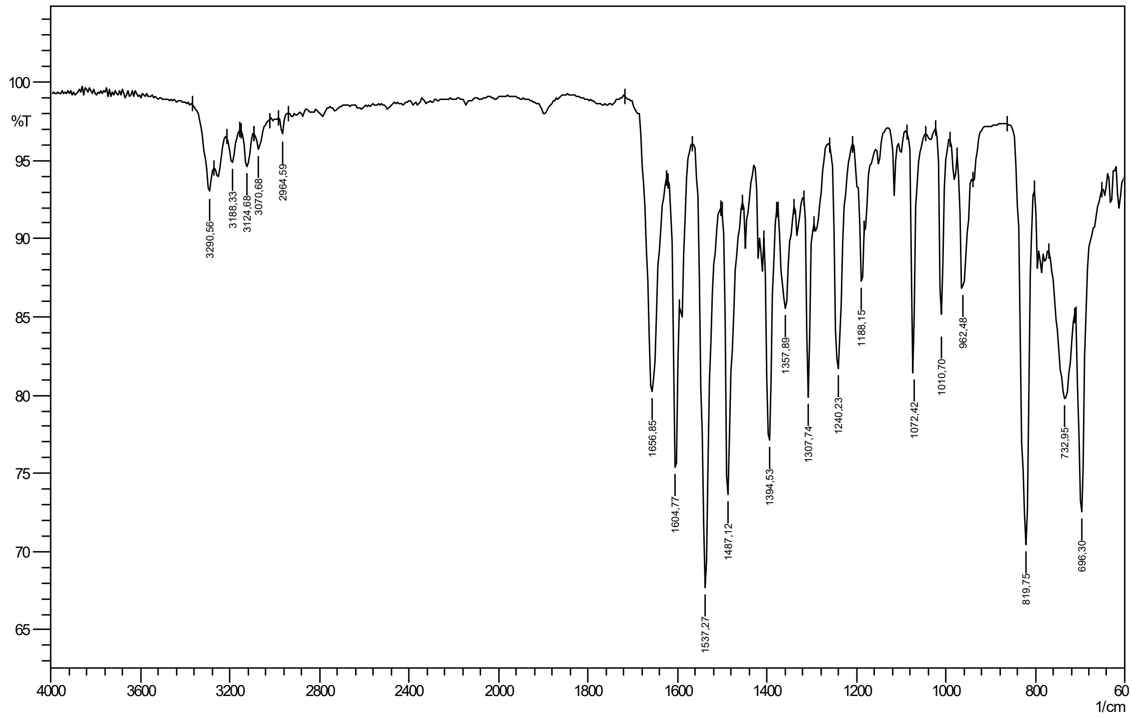

Figure S40. Infrared spectrum (ATR) of 3-chloro-N-(4-bromophenyl)propanamide (NC19) (Bromine).

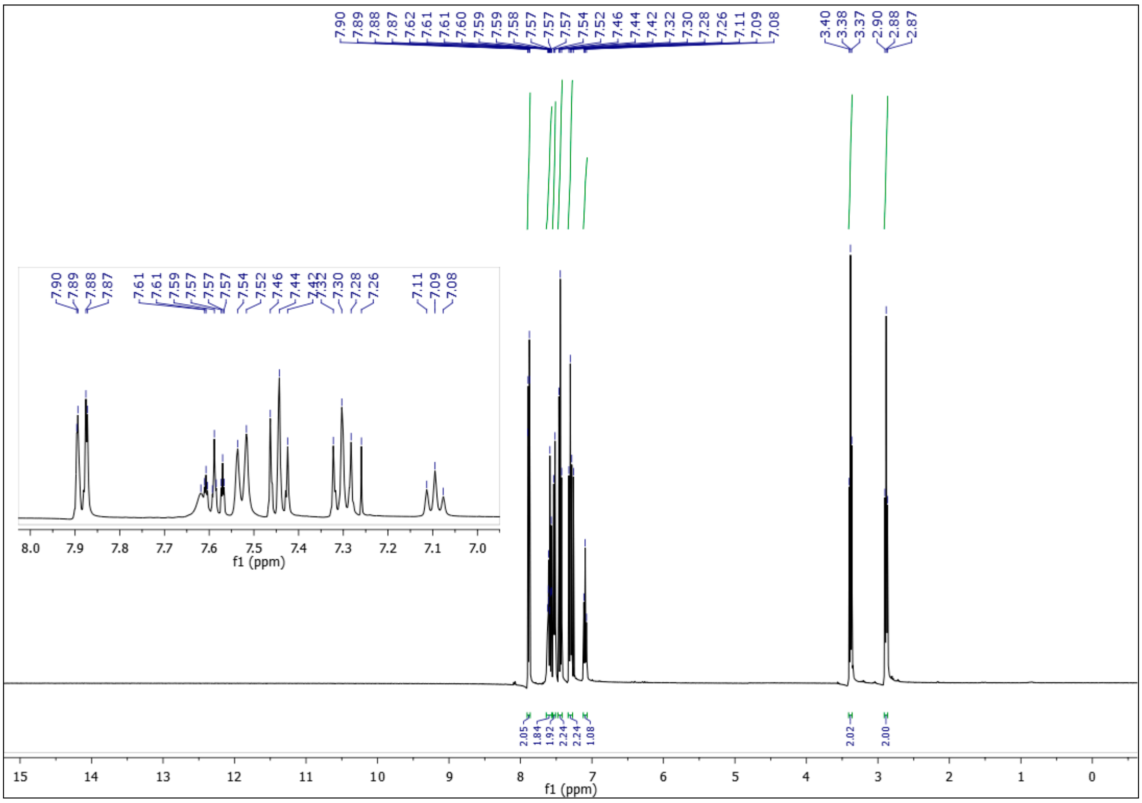

Figure S41. Hydrogen spectrum (400 MHz, CDCl3) of N-phenylbenzoselenoethylenelacticamide (NC30).

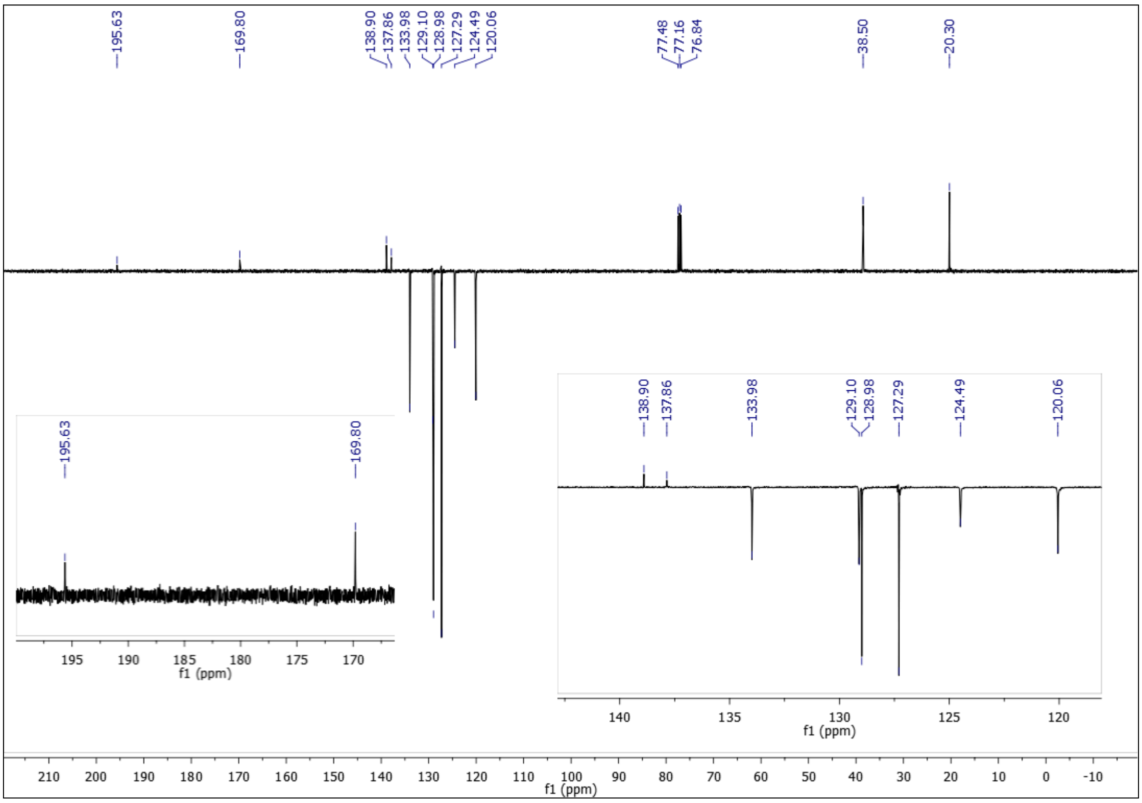

Figure S42. Carbon spectrum (101 MHz, CDCl3) of N-phenylbenzoselenoethylenelacticamide (NC30).

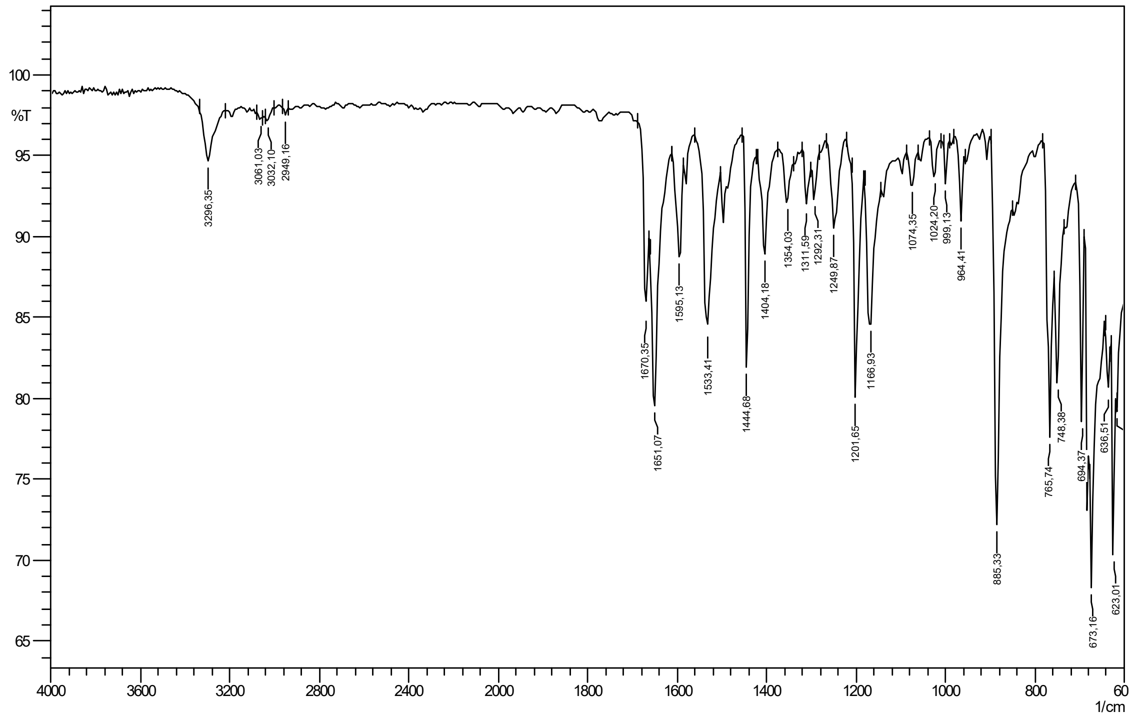

Figure S43. Infrared spectrum (ATR) of N-phenylbenzoselenoethylenelacticamide (NC30).

Display Report

Analysis Info

Analysis Name	D:\Data\laiane\Natalia\Natalia_SeB1_1-1_01_394.d	Acquisition Date	26/11/2019 16:17:20
Method	tune_low_POS_ID+LC.m	Operator	BDAL@DE
Sample Name	Natalia_SeB1	Instrument / Ser#	micrOTOF 10338
Comment			

Acquisition Parameter

Source Type	ESI	Ion Polarity	Positive	Set Nebulizer	49.3 psi
Focus	Not active			Set Dry Heater	300 °C
Scan Begin	50 m/z	Set Capillary	4500 V	Set Dry Gas	8.0 l/min
Scan End	1500 m/z	Set End Plate Offset	-500 V	Set Divert Valve	Waste

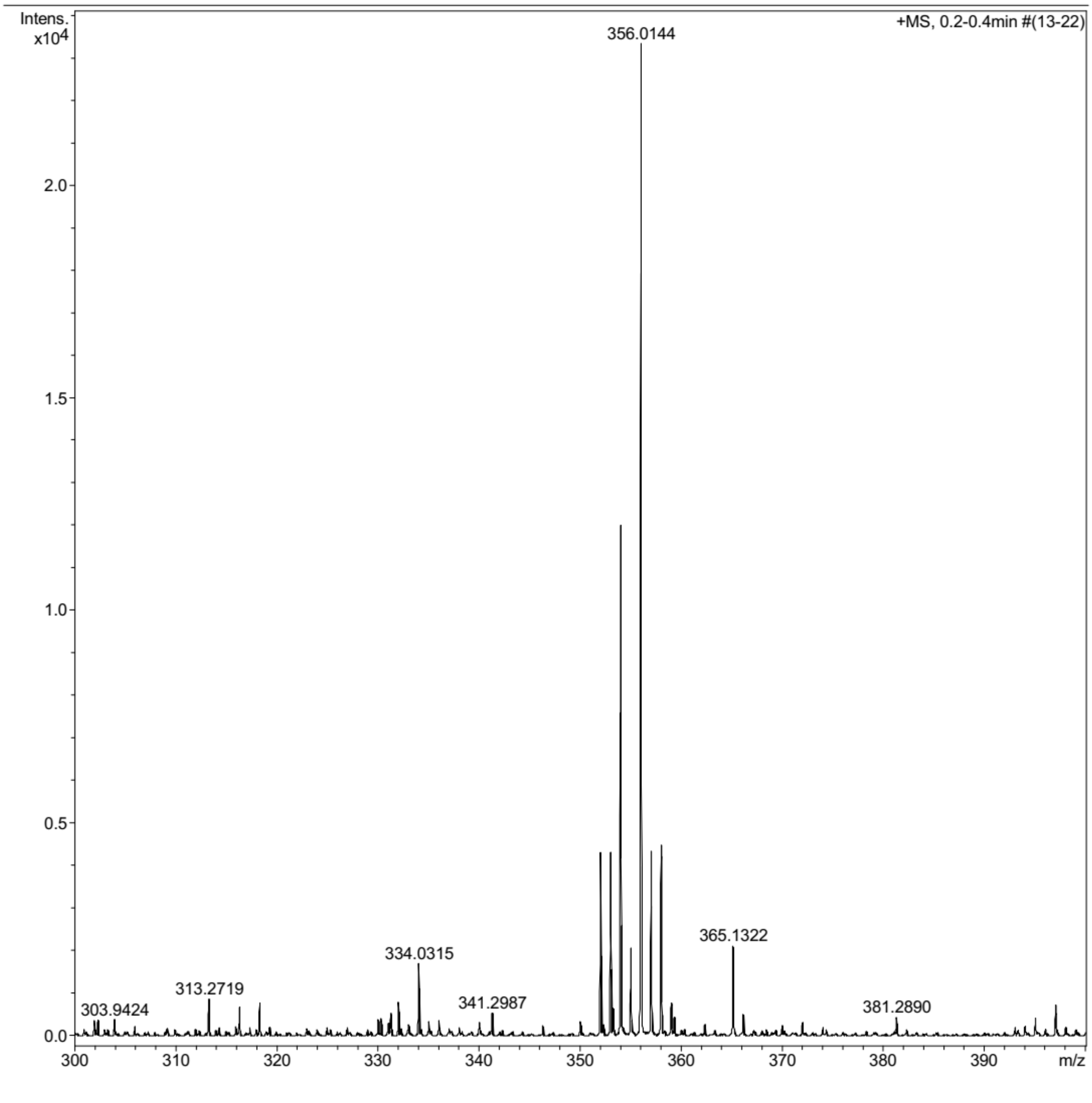

Bruker Compass DataAnalysis 4.0 printed: 26/11/2019 16:31:11 Page 1 of 1

Figure S44. Mass Spectrum of N-phenylbenzoselenoethylenelactamide (NC30).

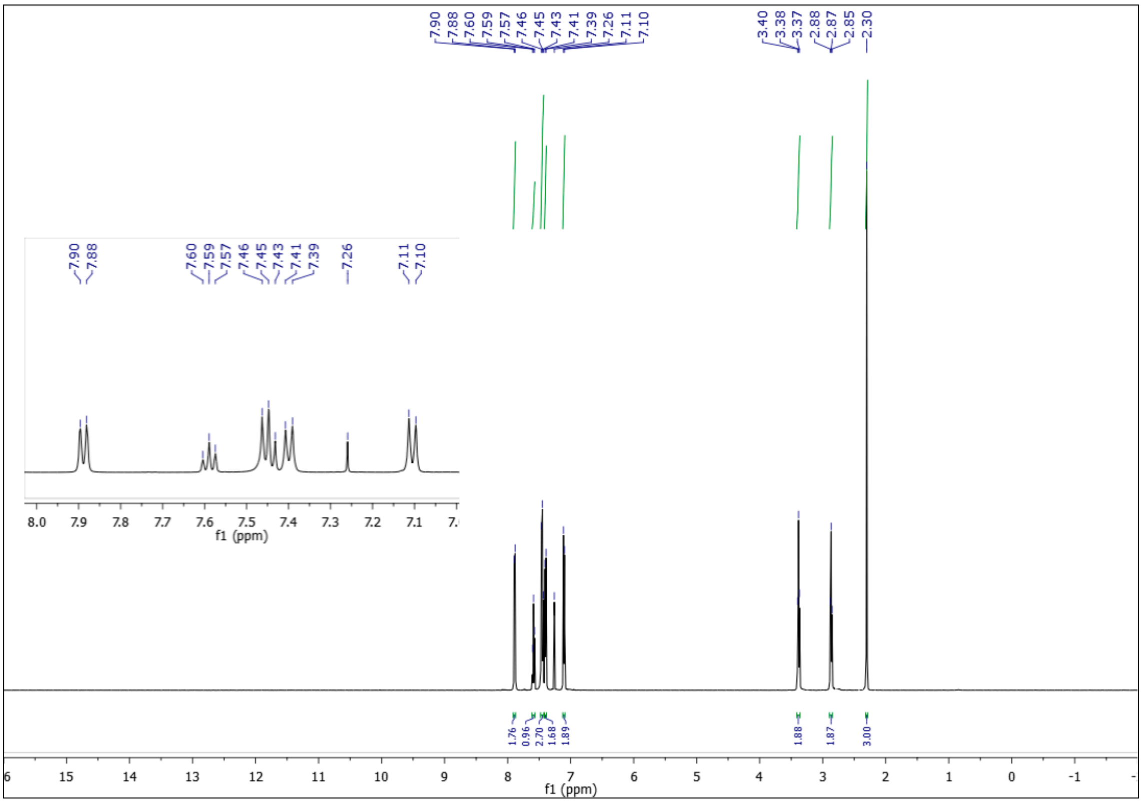

Figure S45. Hydrogen spectrum (500 MHz, CDCl3) of N-(4-methylphenyl)benzoseleneethylenelactamide NC31.

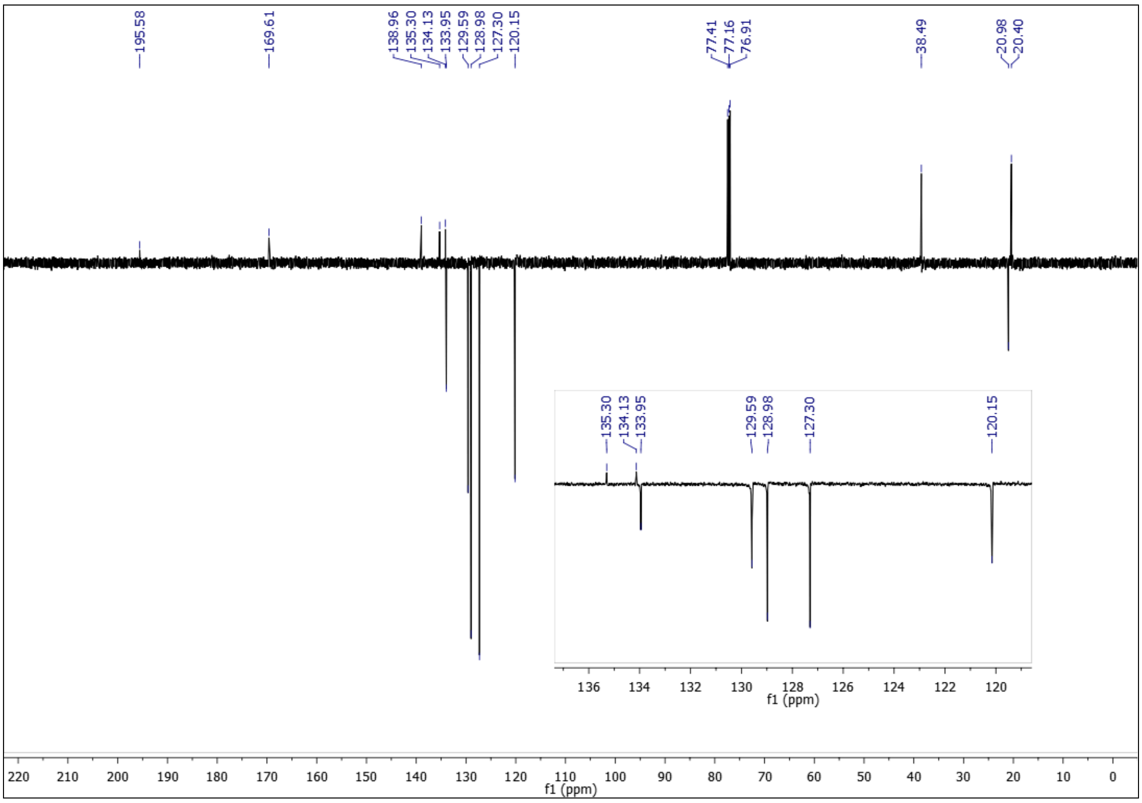

Figure S46. Carbon spectrum (101 MHz, CDCl3) of N-(4-methylphenyl)benzoseleneethylenelacticamide (NC31).

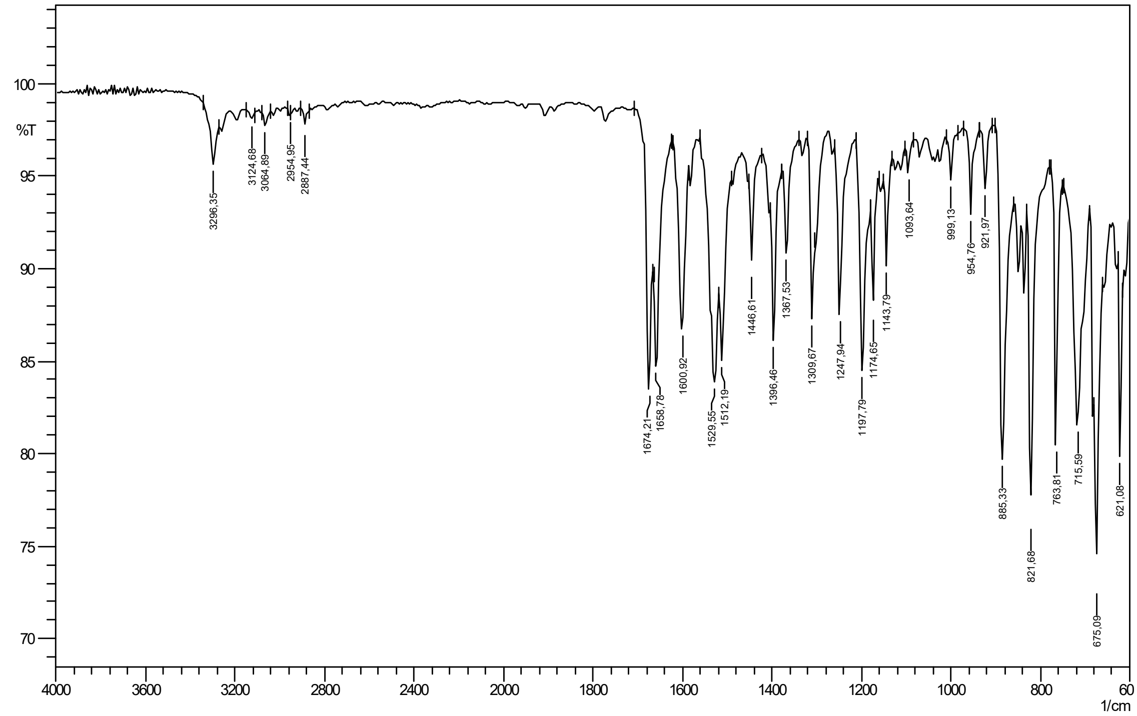

Figure S47. Infrared spectrum (ATR) of N-(4-methylphenyl) benzoselenoethylene lacticamide (NC31).

Display Report

Analysis Info

Analysis Name	D:\Data\laiane\Natalia\Natalia_SeB2_1-5_01_398.d	Acquisition Date	26/11/2019 16:32:06
Method	tune_low_POS_ID+LC.m	Operator	BDAL@DE
Sample Name	Natalia_SeB2	Instrument / Ser#	micrOTOF 10338
Comment			

Acquisition Parameter

Source Type	ESI	Ion Polarity	Positive	Set Nebulizer	49.3 psi
Focus	Not active			Set Dry Heater	300 °C
Scan Begin	50 m/z	Set Capillary	4500 V	Set Dry Gas	8.0 l/min
Scan End	1500 m/z	Set End Plate Offset	-500 V	Set Divert Valve	Waste

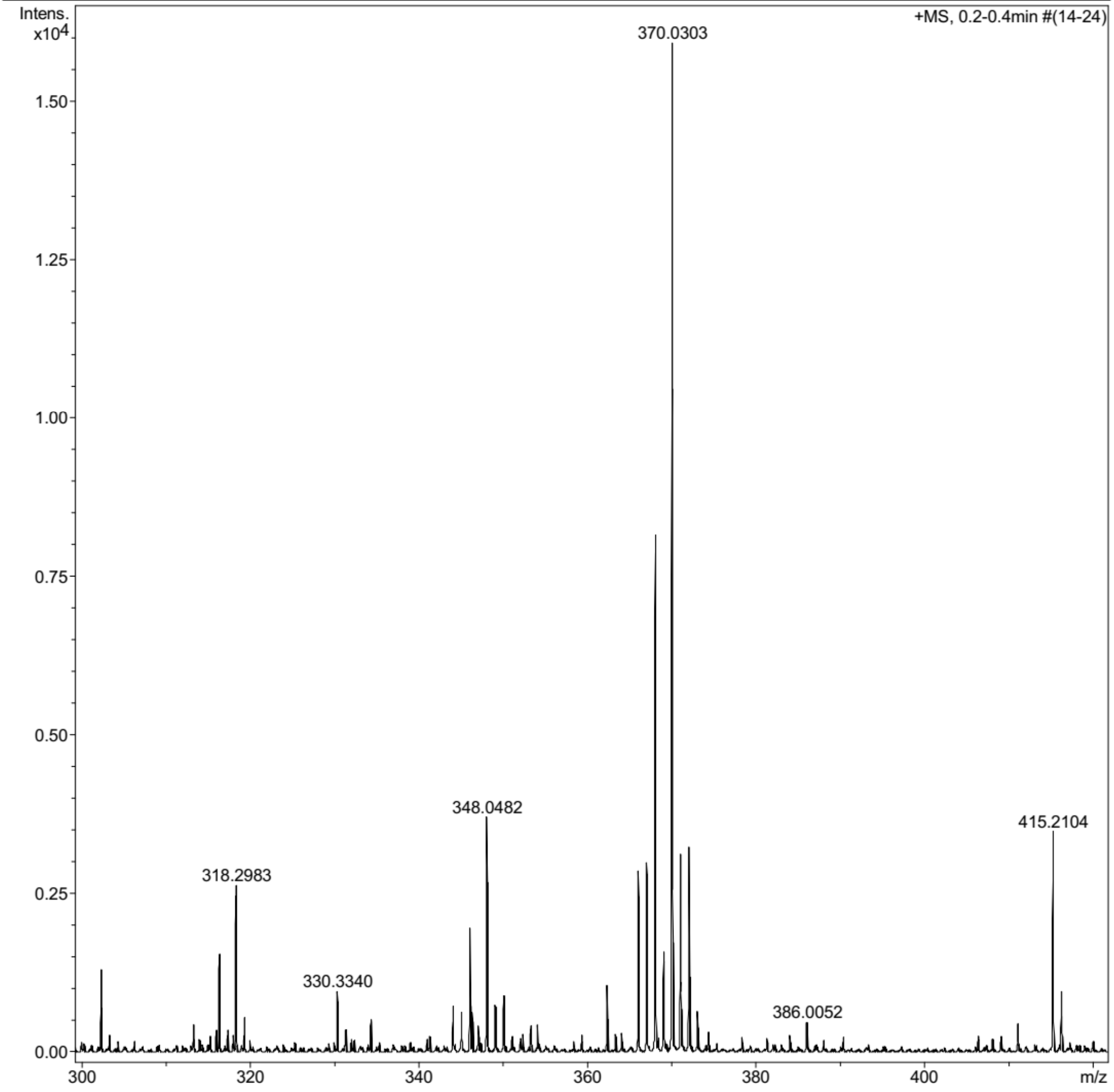

Bruker Compass DataAnalysis 4.0 printed: 26/11/2019 16:47:26 Page 1 of 1

Figure S48. Mass spectrum of N-(methylphenyl)benzoseleneethylenelactamide (NC31).

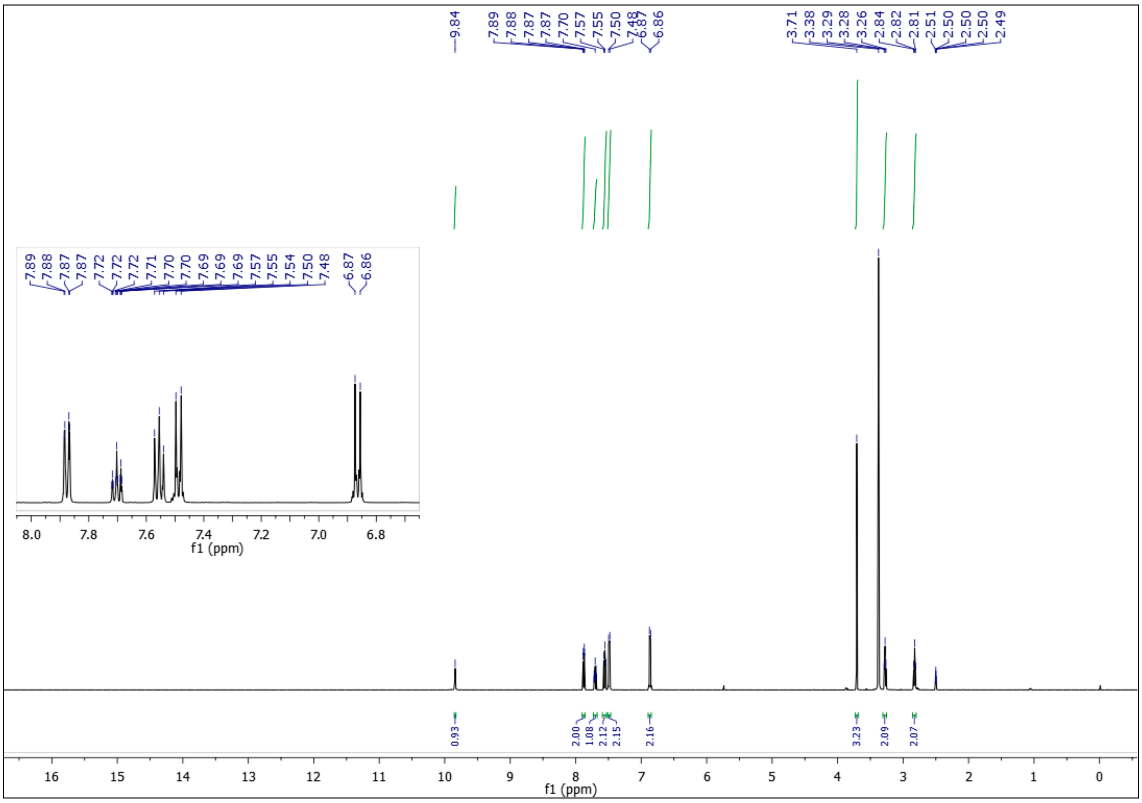

Figure S49. Hydrogen spectrum (500 MHz, DMSO) of N-(4-methoxyphenyl) benzoselenoethylenelacticamide (NC34).

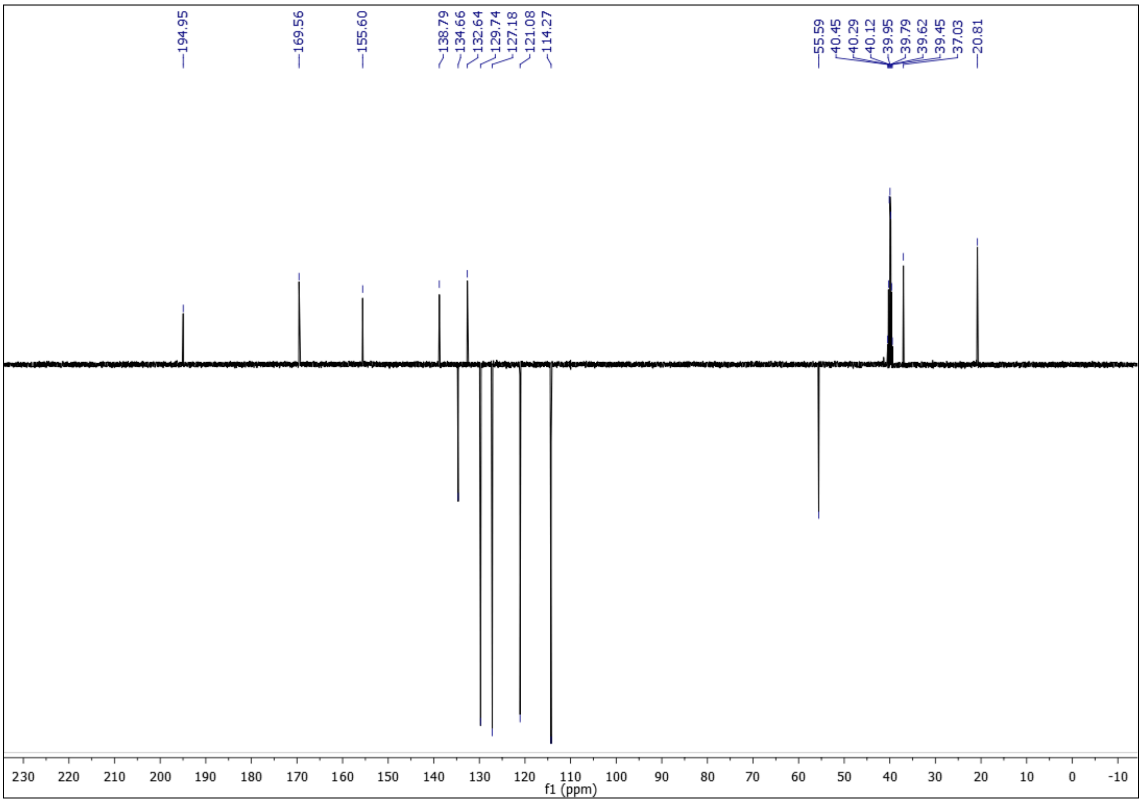

Figure S50. Carbon spectrum (126 MHz, dmso) of N-(4-methoxyphenyl)benzoseleneethylenelacticamide (NC34).

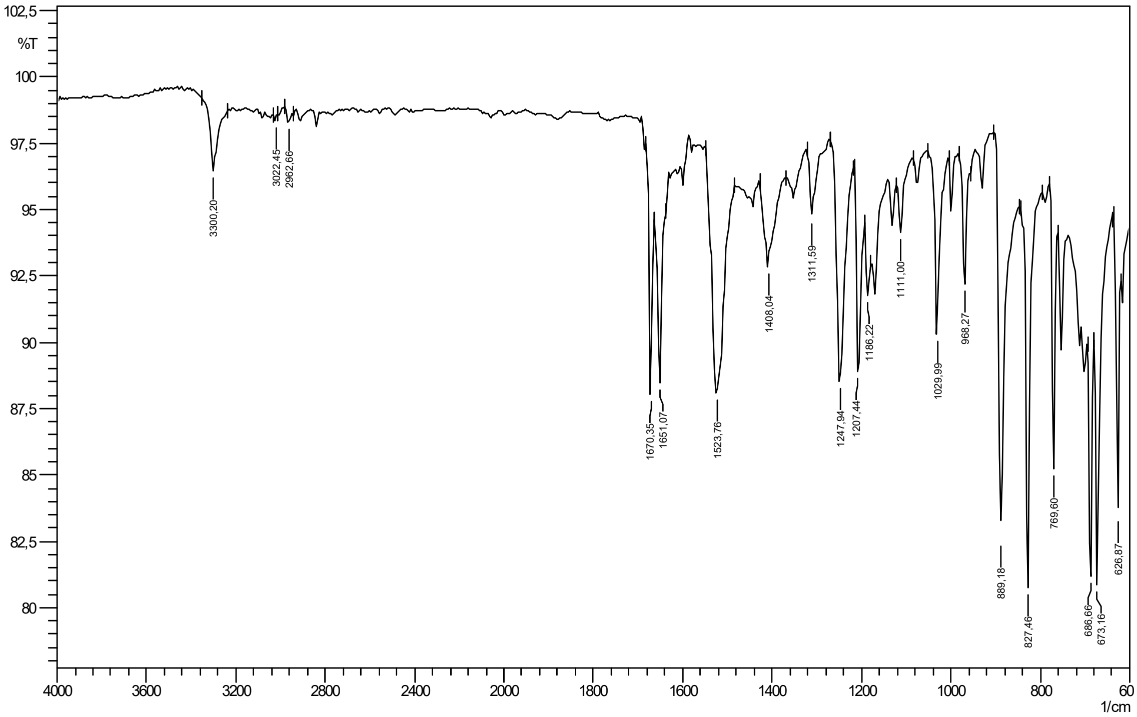

Figure S51. Infrared spectrum (ATR) of N-(4-methoxyphenyl) benzoselenoethylene lacticamide (NC34).

Display Report

Analysis Info

Analysis Name	D:\Data\laiane\Natalia\Natalia_SeB3_1112_1-2_01_441.d	Acquisition Date	11/12/2019 16:07:13
Method	tune_low_POS_ID+LC.m	Operator	BDAL@DE
Sample Name	Natalia_SeB3_1112	Instrument / Ser#	micrOTOF 10338
Comment			

Acquisition Parameter

Source Type	ESI	Ion Polarity	Positive	Set Nebulizer	49.3 psi
Focus	Not active			Set Dry Heater	300 °C
Scan Begin	50 m/z	Set Capillary	4500 V	Set Dry Gas	8.0 l/min
Scan End	1500 m/z	Set End Plate Offset	-500 V	Set Divert Valve	Waste

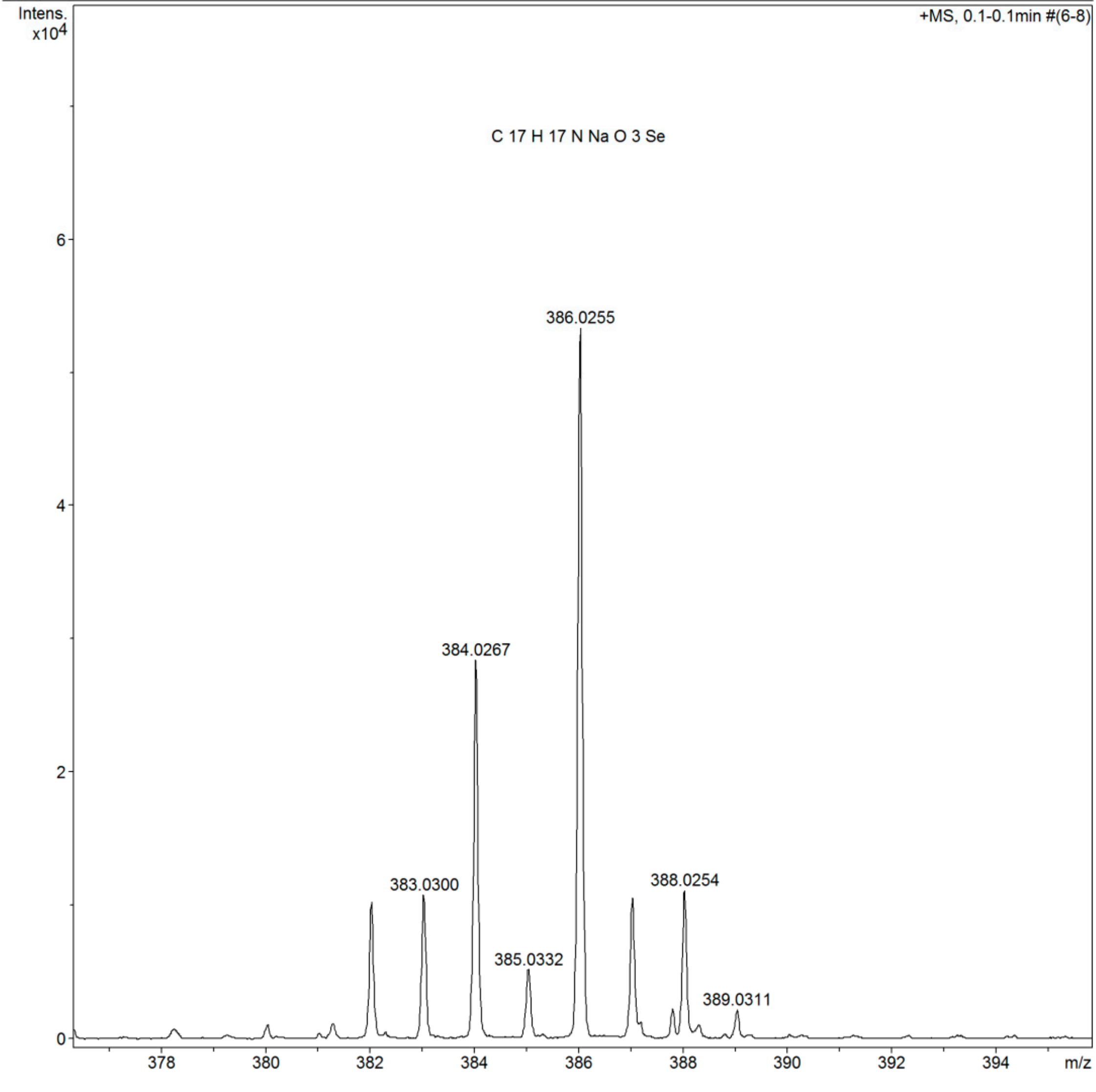

Bruker Compass DataAnalysis 4.0 printed: 18/12/2019 14:38:28 Page 1 of 1

Figure S52. Mass Spectrum of N-(4-methoxyphenyl)benzoselenoethylenelactamide (NC34).

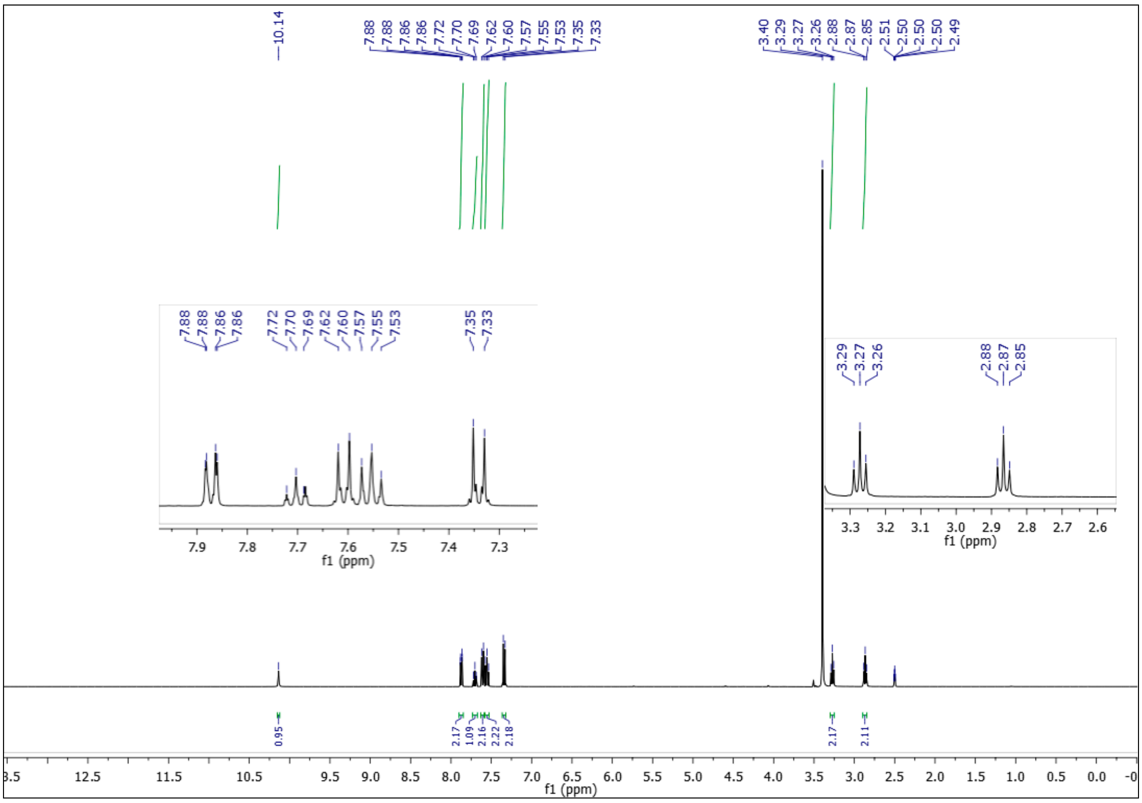

Figure S53. Hydrogen spectrum (400 MHz DMSO-d6) of N-(4-chlorophenyl)benzoseleneethylenelacticamide (NC36).

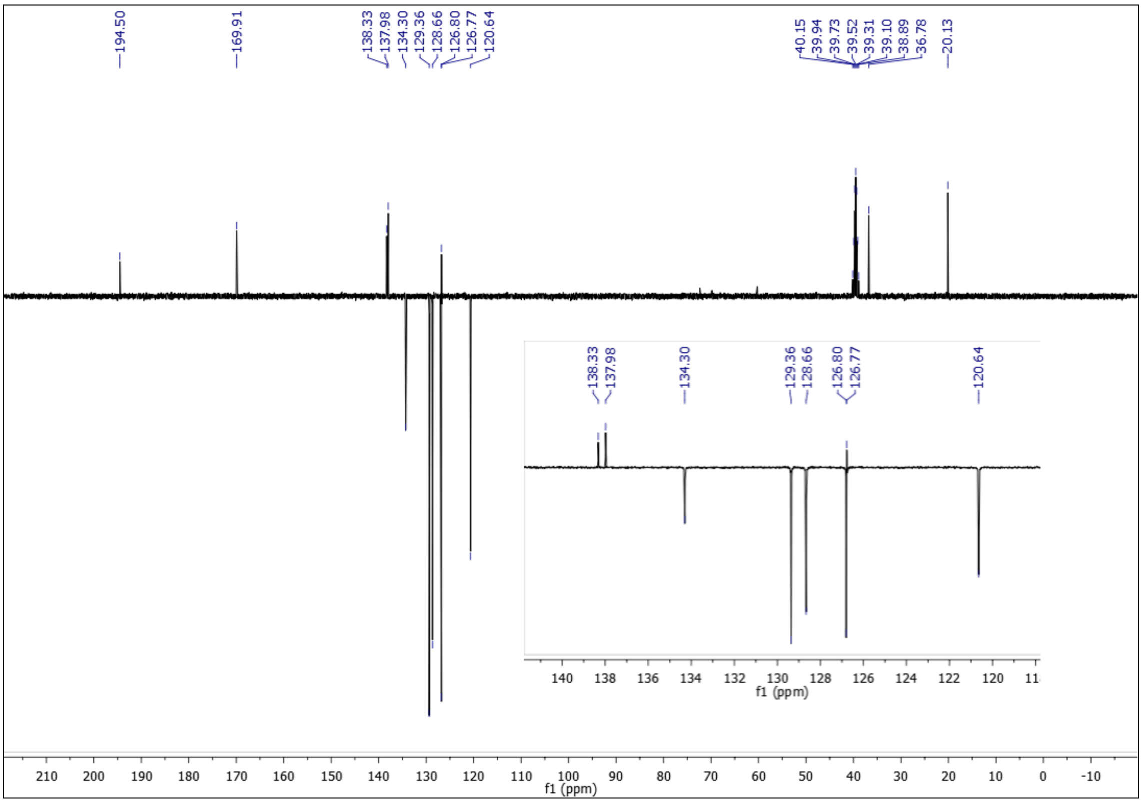

Figure S54. Carbon spectrum (101 MHz, DMSO-d6) of N-(4-chlorophenyl)benzoseleneethylenelacticamide (NC36).

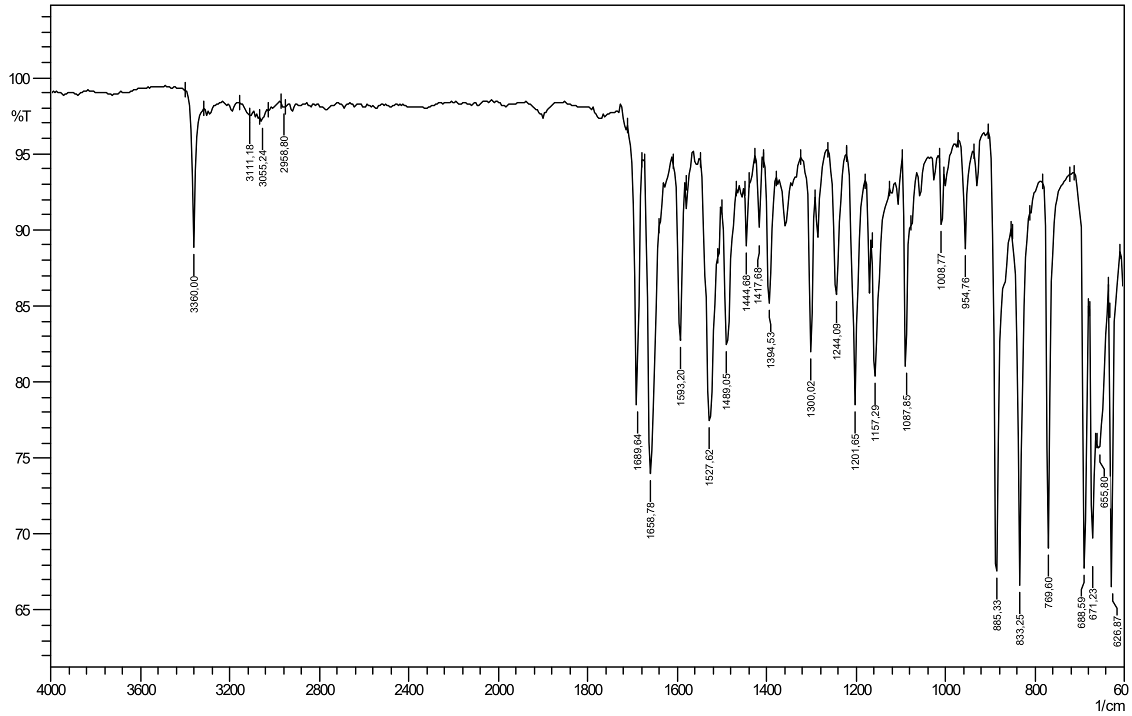

Figure S55. Infrared spectrum (ATR) of N-(4-chlorophenyl)benzoselenoethylenelacticamide (NC36).

Display Report

Analysis Info

Analysis Name	D:\Data\laiane\Natalia\Natalia_SeB5_2_1-3_01_442.d	Acquisition Date	11/12/2019 16:10:54
Method	tune_low_POS_ID+LC.m	Operator	BDAL@DE
Sample Name	Natalia_SeB5_2	Instrument / Ser#	micrOTOF 10338
Comment			

Acquisition Parameter

Source Type	ESI	Ion Polarity	Positive	Set Nebulizer	49.3 psi
Focus	Not active			Set Dry Heater	300 °C
Scan Begin	50 m/z	Set Capillary	4500 V	Set Dry Gas	8.0 l/min
Scan End	1500 m/z	Set End Plate Offset	-500 V	Set Divert Valve	Waste

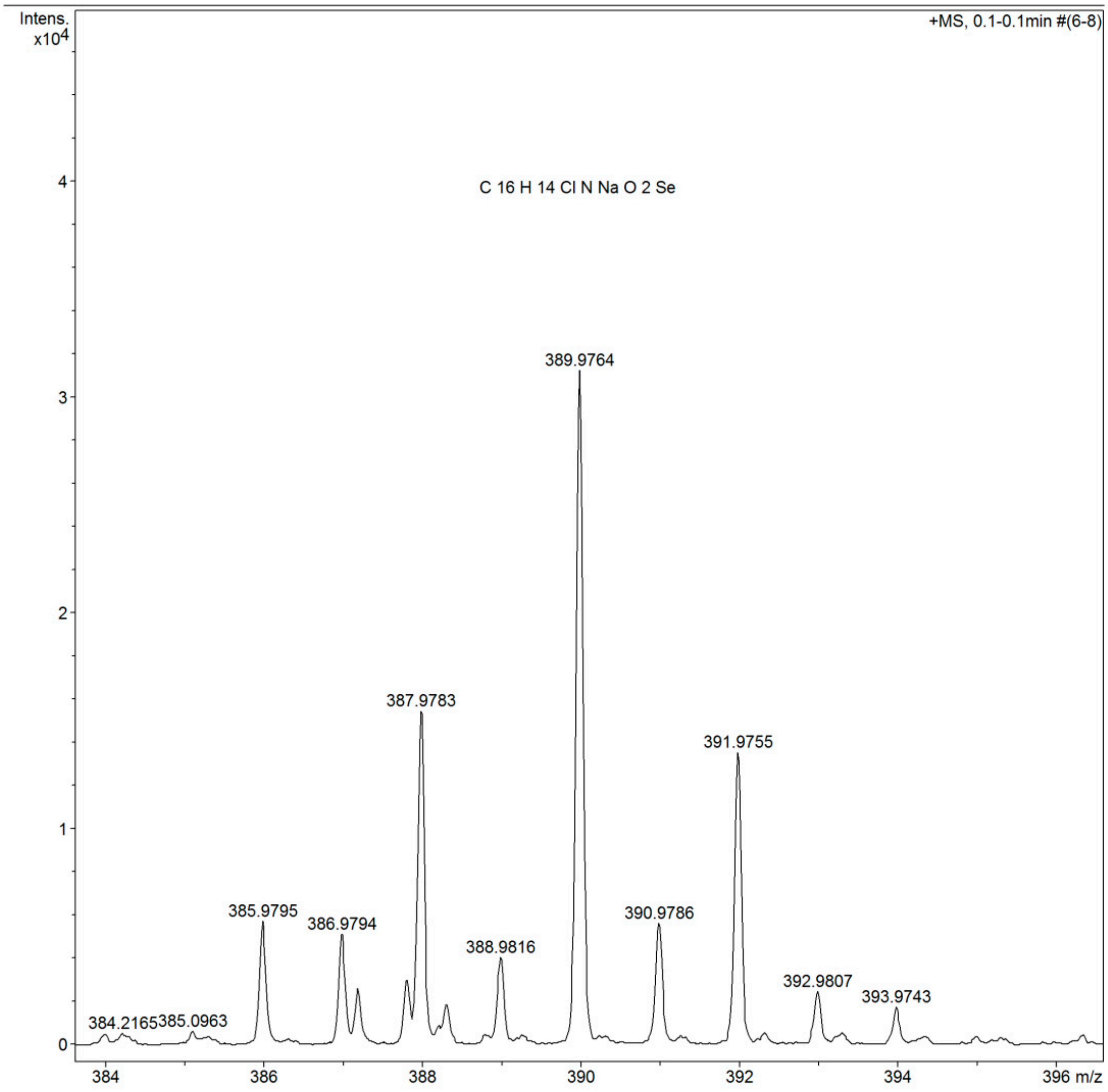

Bruker Compass DataAnalysis 4.0 printed: 18/12/2019 15:07:43 Page 1 of 1

Figure S56. Mass Spectrum of N-(4-chlorophenyl)benzoseleneethylenelactamide (NC36).

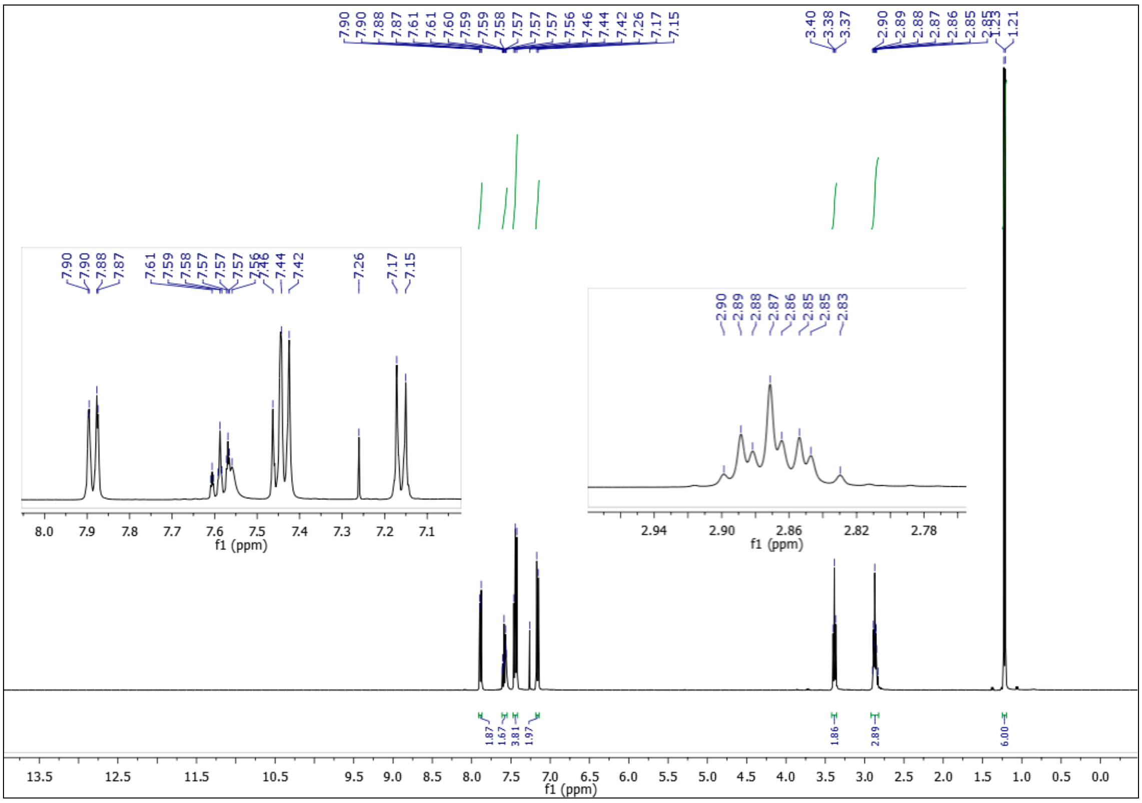

Figure S57. Hydrogen spectrum (500 MHz, CDCl3) of N-(4-isopropylphenyl)benzoseleneethylenelacticamide (NC40).

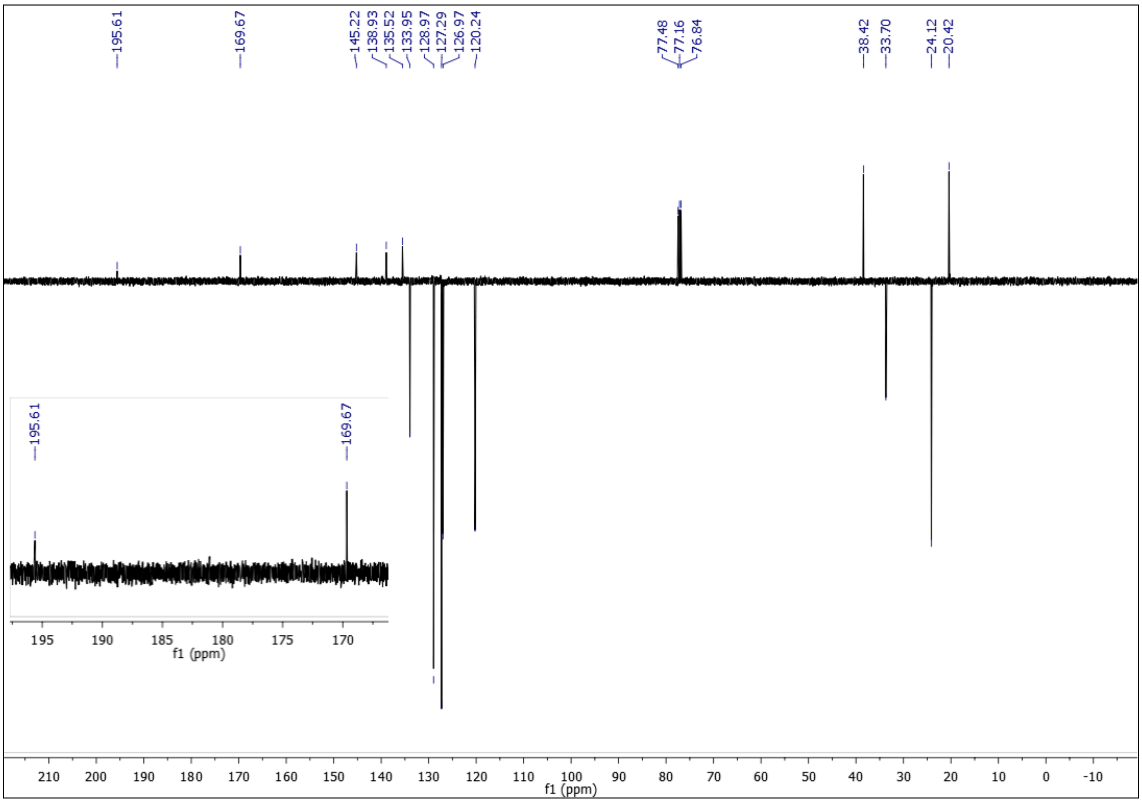

Figure S58. Carbon spectrum (101 MHz, CDCl3) of N-(4-isopropylphenyl)benzoselenoethylenelacticamide (NC40).

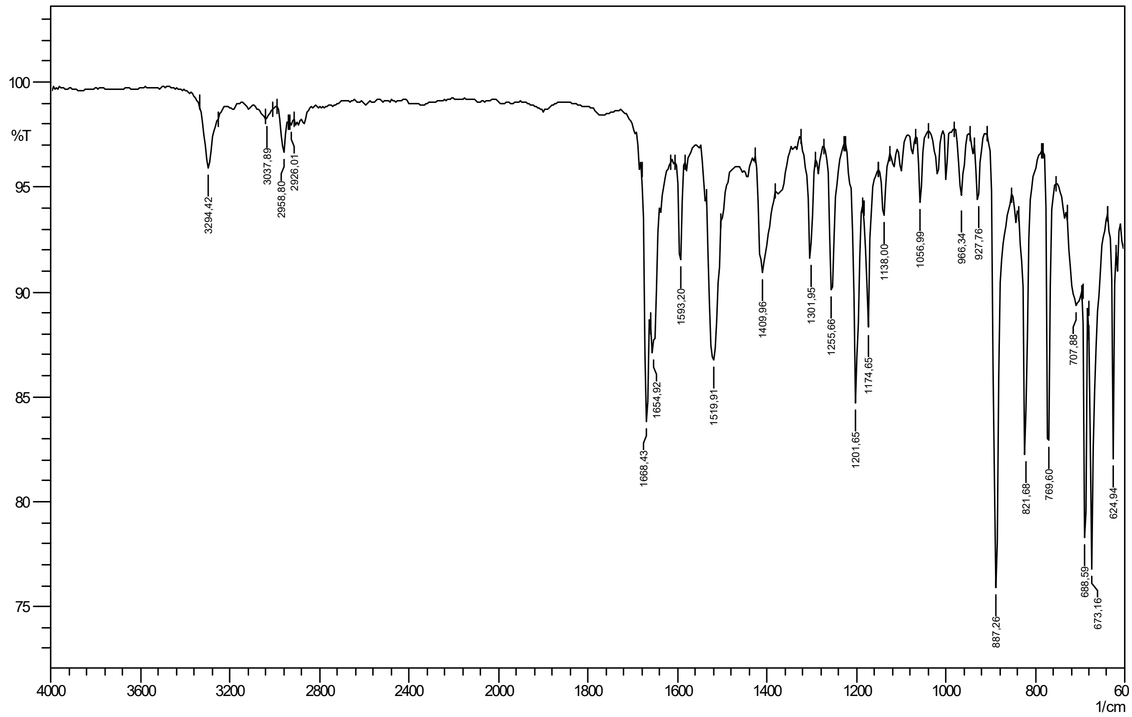

Figure S59. Infrared (ATR) spectrum of N-(4-isopropylphenyl)benzoselenoethylenelacticamide (NC40).

Display Report

Analysis Info

Analysis Name	D:\Data\laiane\Natalia\Natalia_SeB4_1-4_01_397.d	Acquisition Date	26/11/2019 16:28:25
Method	tune_low_POS_ID+LC.m	Operator	BDAL@DE
Sample Name	Natalia_SeB4	Instrument / Ser#	micrOTOF 10338
Comment			

Acquisition Parameter

Source Type	ESI	Ion Polarity	Positive	Set Nebulizer	49.3 psi
Focus	Not active			Set Dry Heater	300 °C
Scan Begin	50 m/z	Set Capillary	4500 V	Set Dry Gas	8.0 l/min
Scan End	1500 m/z	Set End Plate Offset	-500 V	Set Divert Valve	Waste

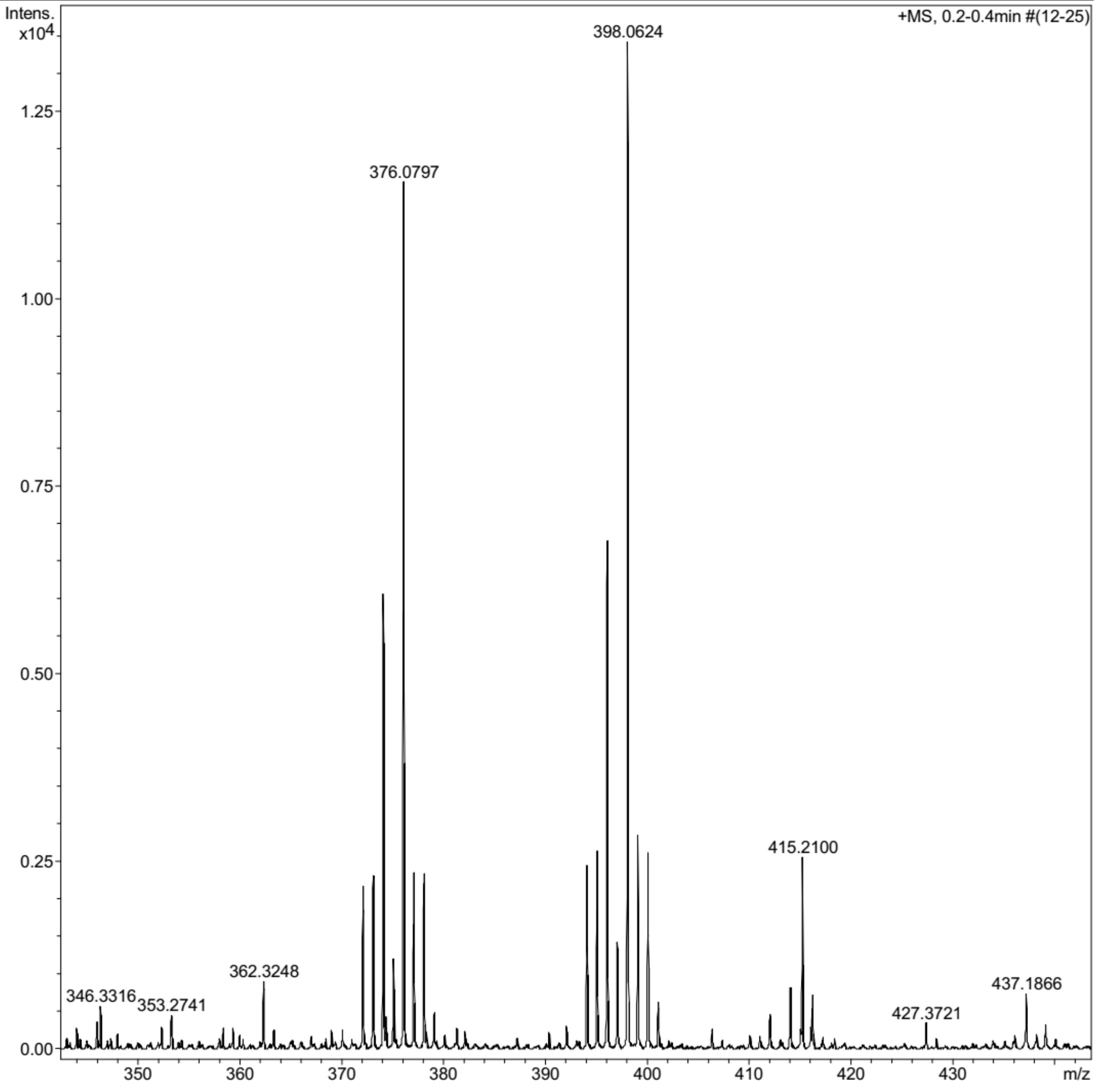

Bruker Compass DataAnalysis 4.0 printed: 26/11/2019 16:39:44 Page 1 of 1

Figure S60. Mass Spectrum of N-(4-isopropylphenyl)benzoselenoethylenelactamide (NC40).

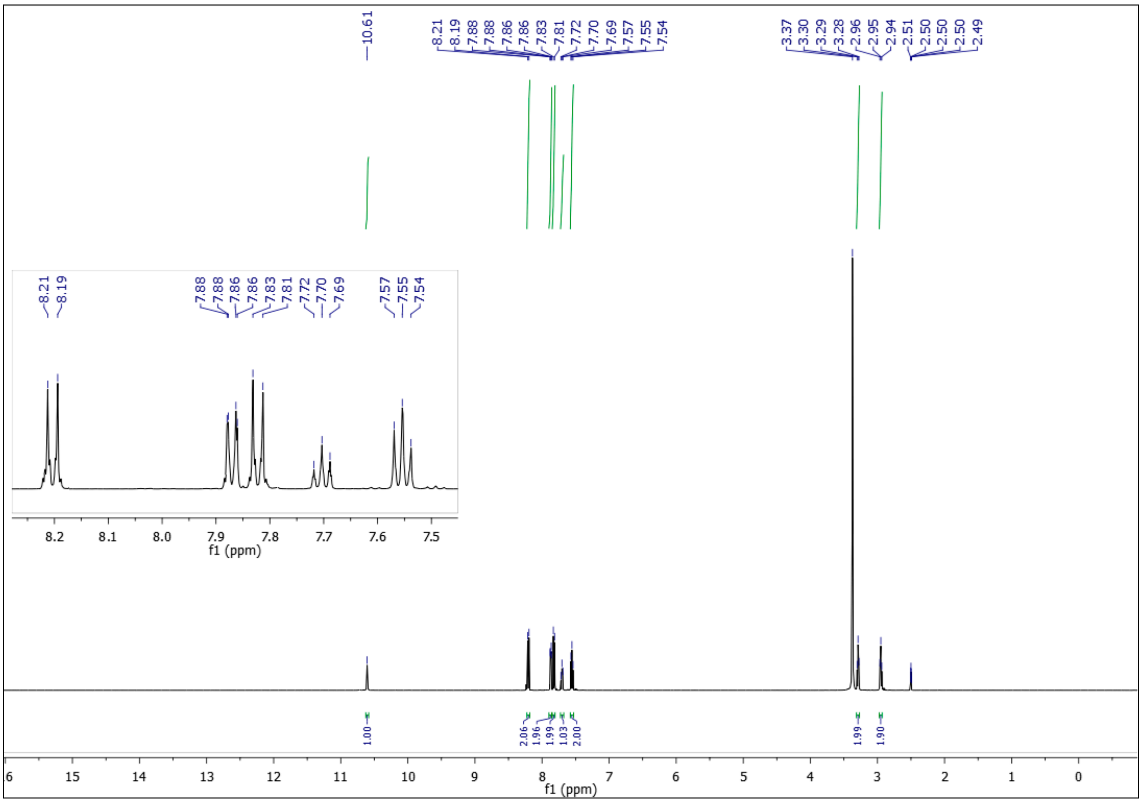

Figure S61. Hydrogen Spectrum (500 MHz, DMSO-d6) of N-(4-nitrophenyl)benzoselenoethylenelacticamide (NC41).

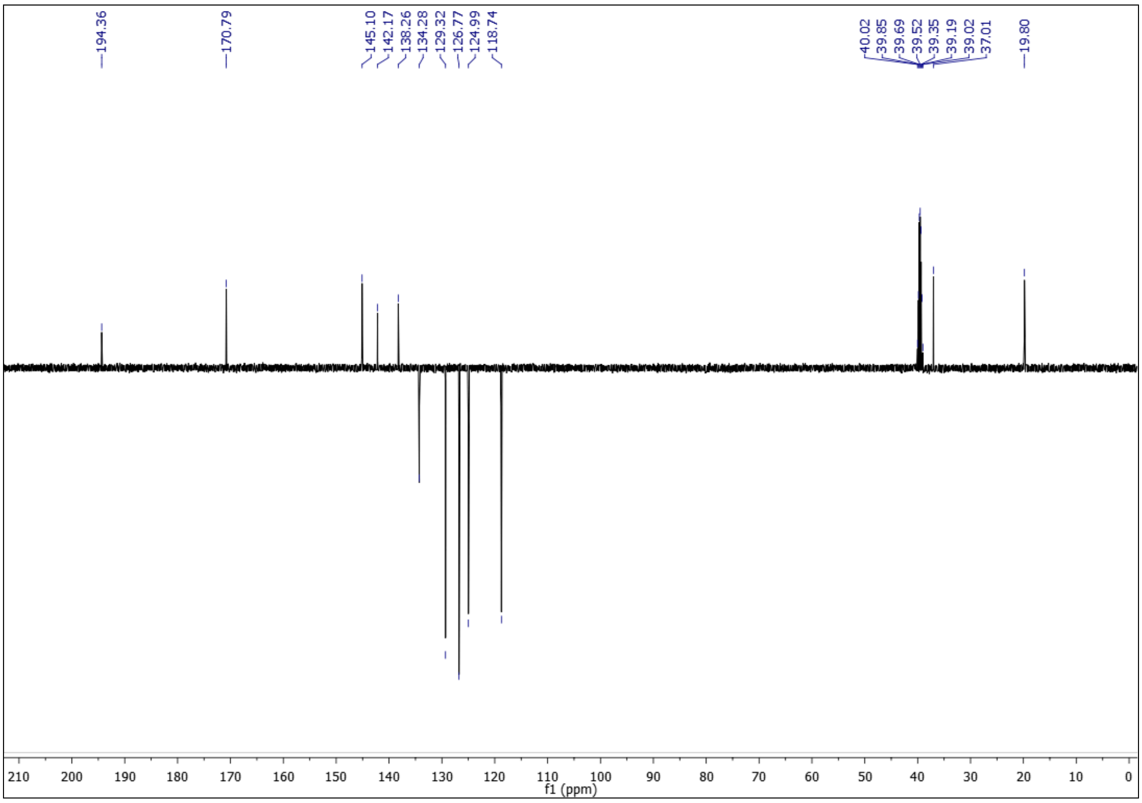

Figure S62. Carbon spectra (126 MHz, DMSO-d6) of N-(4-nitrophenyl)benzoseleneethylenelacticamide (NC41).

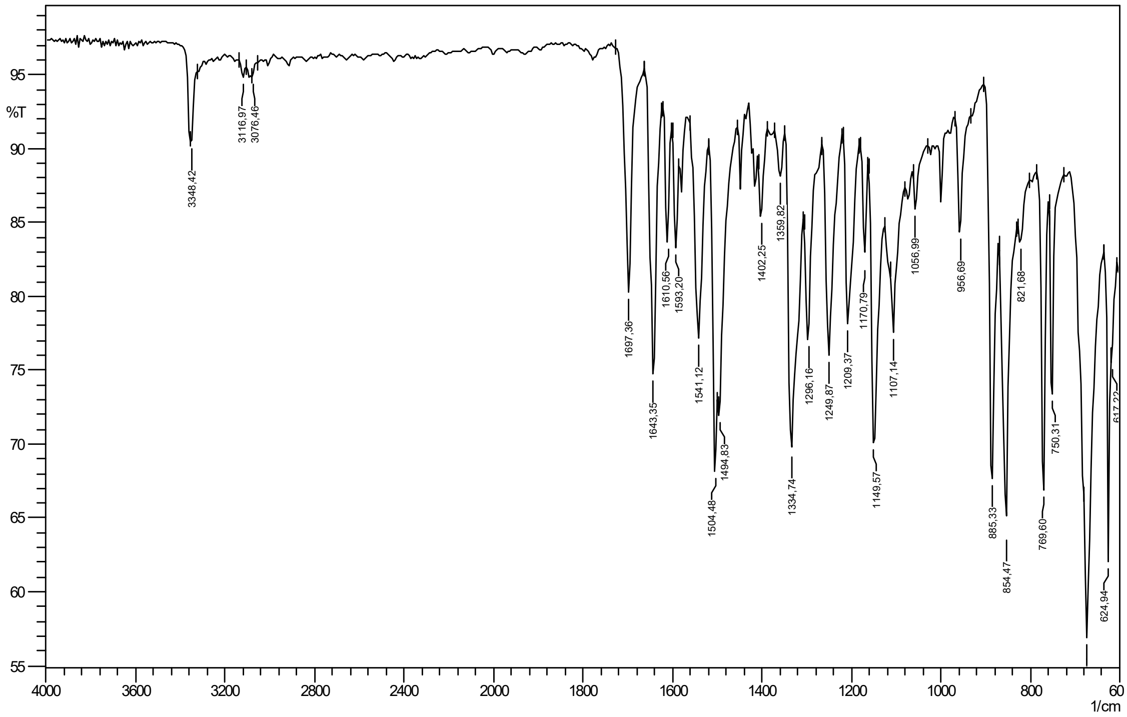

Figure S63. Infrared (ATR) spectrum of N-(4-nitrophenyl)benzoselenoethylenelacticamide (NC41).

Display Report

Analysis Info

Analysis Name	D:\Data\laiane\Natalia\Natalia_SeH1_Nitro_lcms_pos_1-2_01_480.d	Acquisition Date	18/12/2019 14:07:22
Method	tune_low_POS_LC_65MIN.m	Operator	BDAL@DE
Sample Name	Natalia_SeH1_Nitro_lcms_pos	Instrument / Ser#	micrOTOF 10338
Comment			

Acquisition Parameter

Source Type	ESI	Ion Polarity	Positive	Set Nebulizer	49.3 psi
Focus	Not active			Set Dry Heater	300 °C
Scan Begin	50 m/z	Set Capillary	4500 V	Set Dry Gas	8.5 l/min
Scan End	1500 m/z	Set End Plate Offset	-500 V	Set Divert Valve	Waste

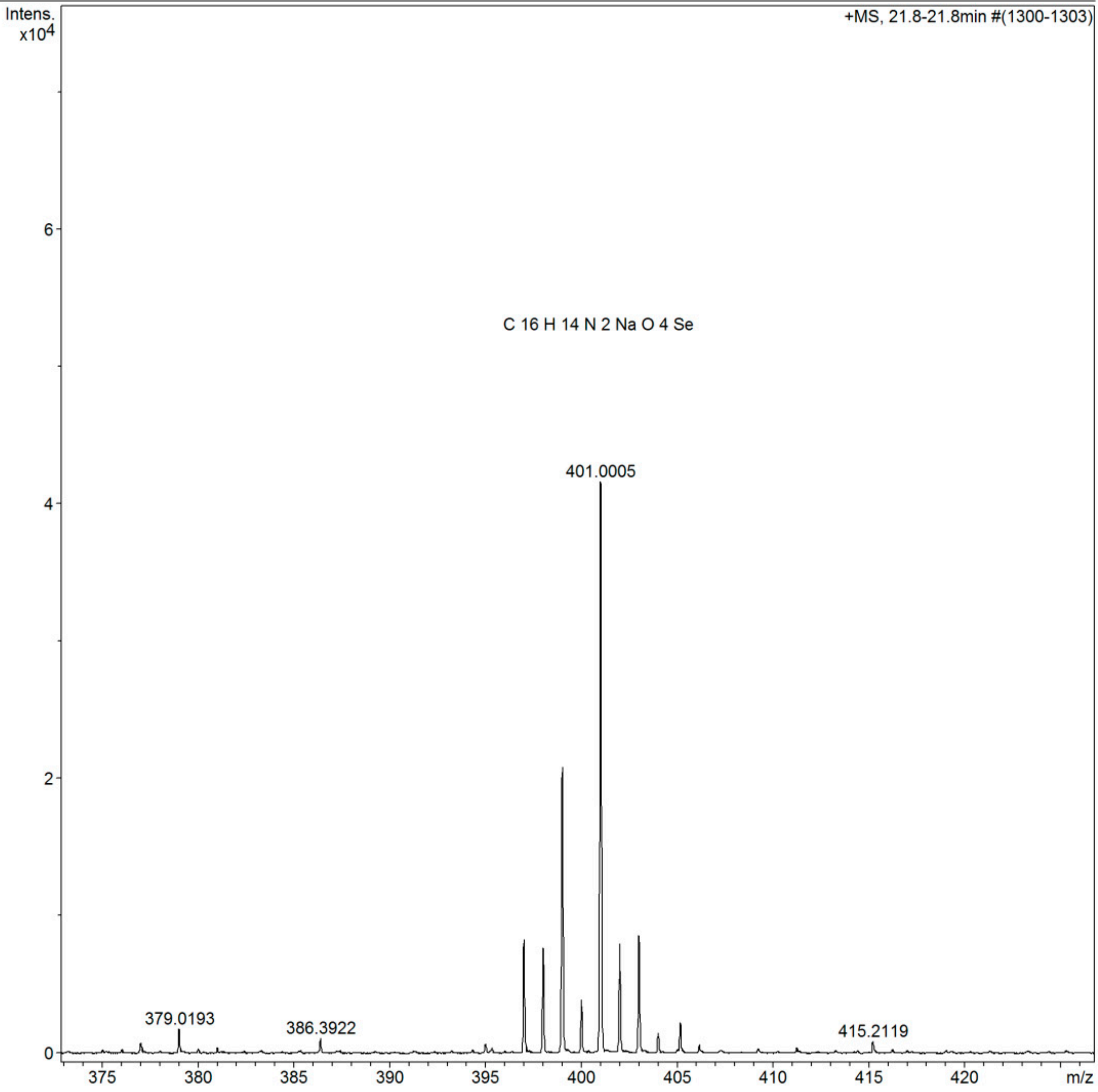

Figure S64. Mass Spectrum of N-(4-nitrophenyl)benzoseleneethylenelactamide (NC41).

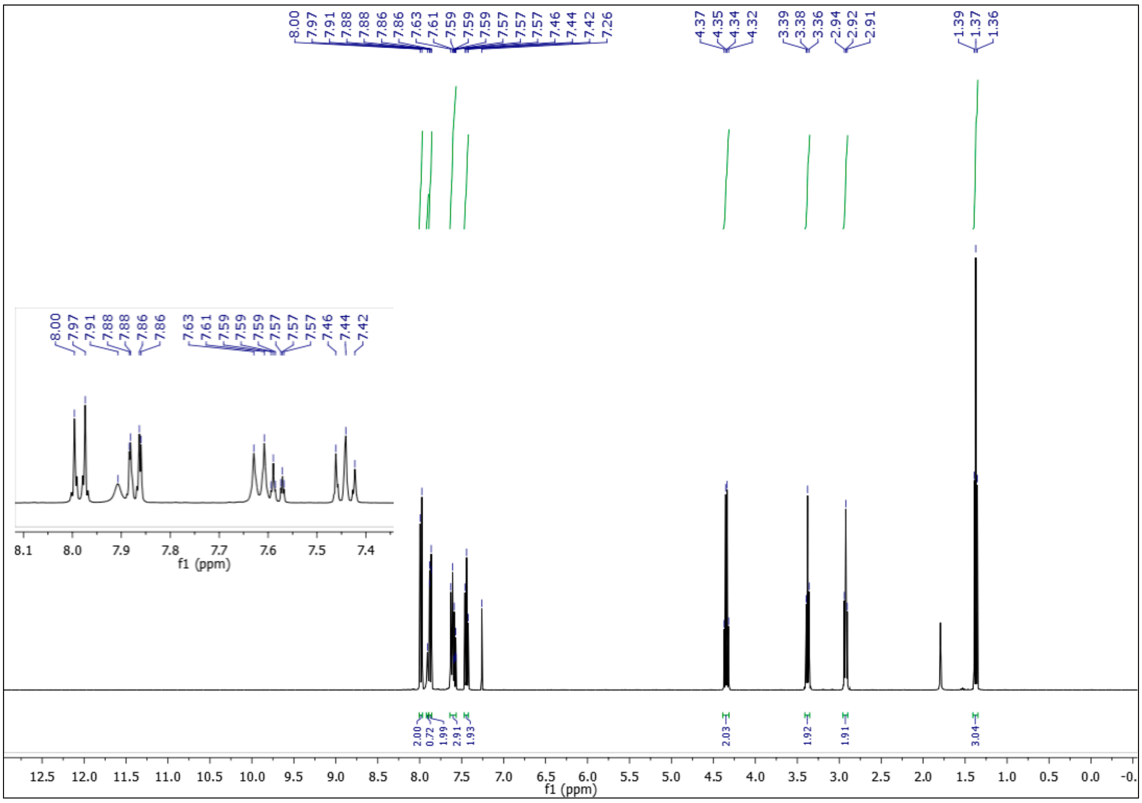

Figure S65. Hydrogen spectrum (400 MHz, CDCl3) of Ethyl (4-benzoselenoethylenelacticamide) Benzoate (NC51).

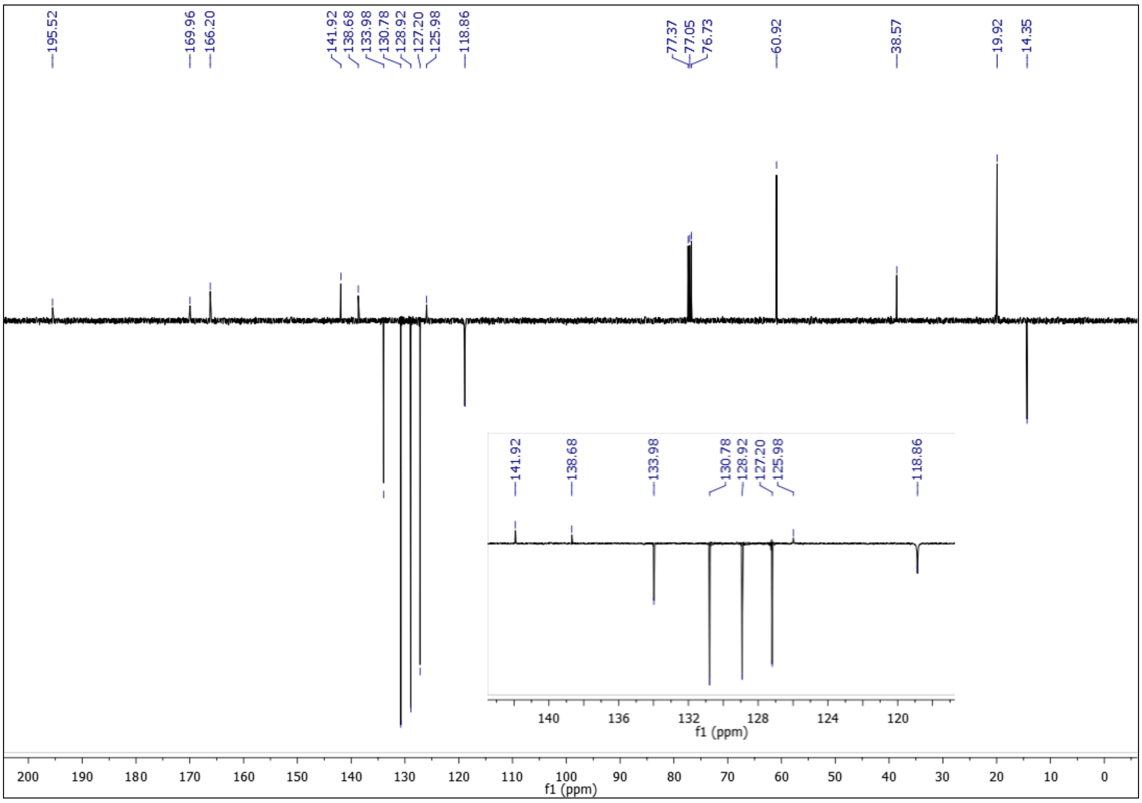

Figure S66. Carbon Spectrum (101 MHz, CDCl 3) of Ethyl (4-benzoselenoethylene lacticamide) Benzoate (NC51).

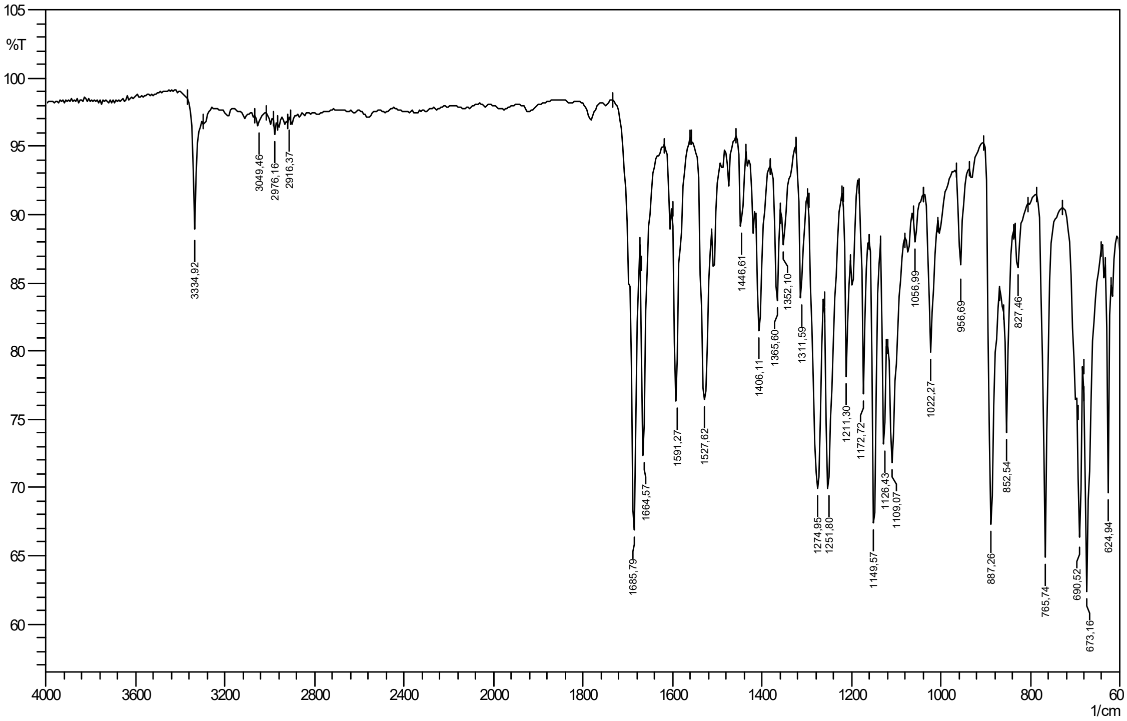

Figure S67. Infrared Spectrum (ATR) of Ethyl (4-benzoselenoethylenelacticamide) Benzoate (NC51).

Display Report

Analysis Info

Analysis Name	D:\Data\laiane\Natalia\Natalia_Se_Benzo_lcms_pos_1uL_1-1_01_479.d	Acquisition Date	18/12/2019 12:58:58
Method	tune_low_POS_LC_65MIN.m	Operator	BDAL@DE
Sample Name	Natalia_Se_Benzo_lcms_pos_1uL	Instrument / Ser#	micrOTOF 10338
Comment			

Acquisition Parameter

Source Type	ESI	Ion Polarity	Positive	Set Nebulizer	49.3 psi
Focus	Not active			Set Dry Heater	300 °C
Scan Begin	50 m/z	Set Capillary	4500 V	Set Dry Gas	8.5 l/min
Scan End	1500 m/z	Set End Plate Offset	-500 V	Set Divert Valve	Waste

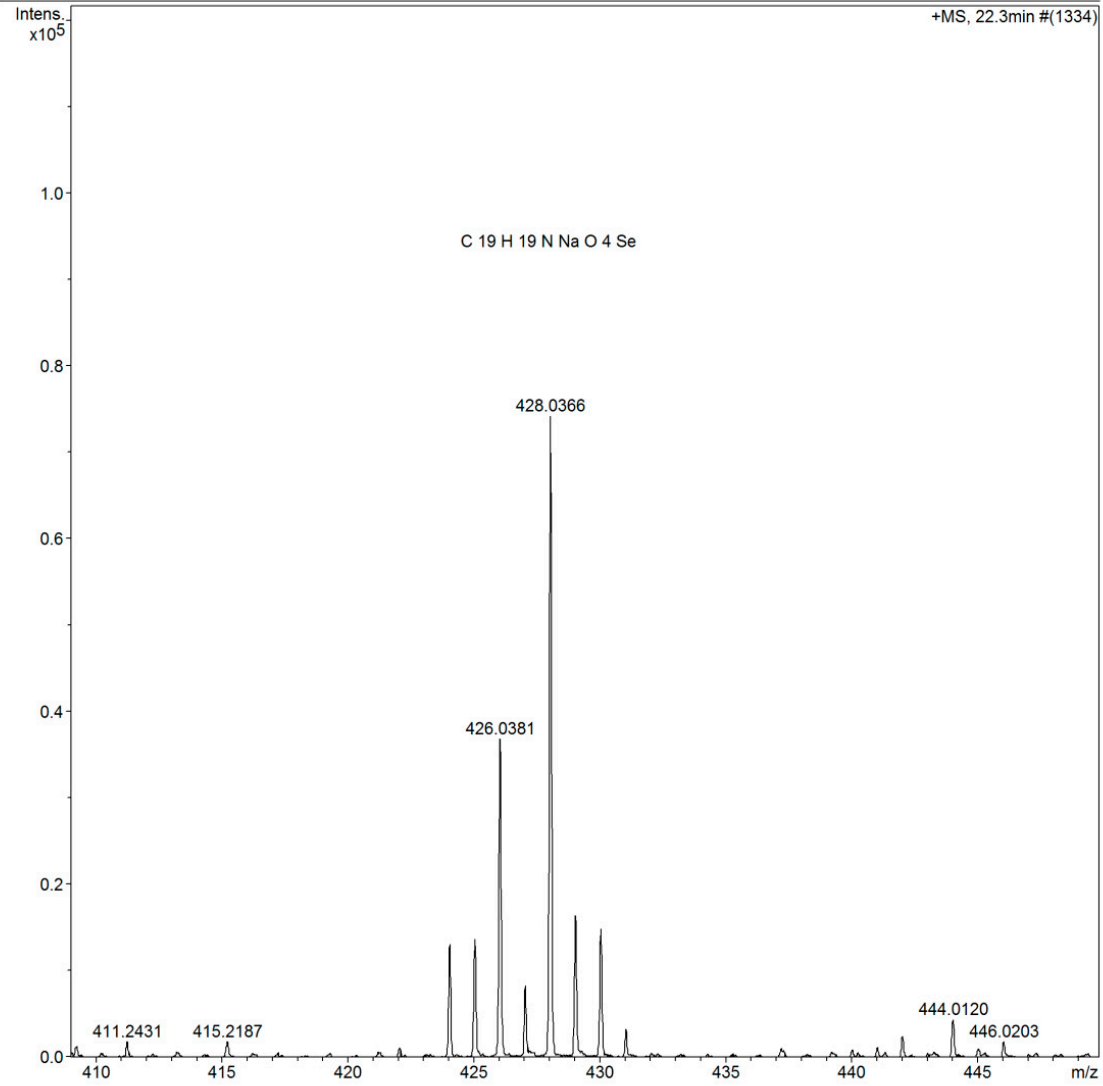

Bruker Compass DataAnalysis 4.0 printed: 18/12/2019 15:32:51 Page 1 of 1

Figure S68. Mass Spectrum Ethyl (4-benzoselenoethylenelactamide) benzoate (NC51).

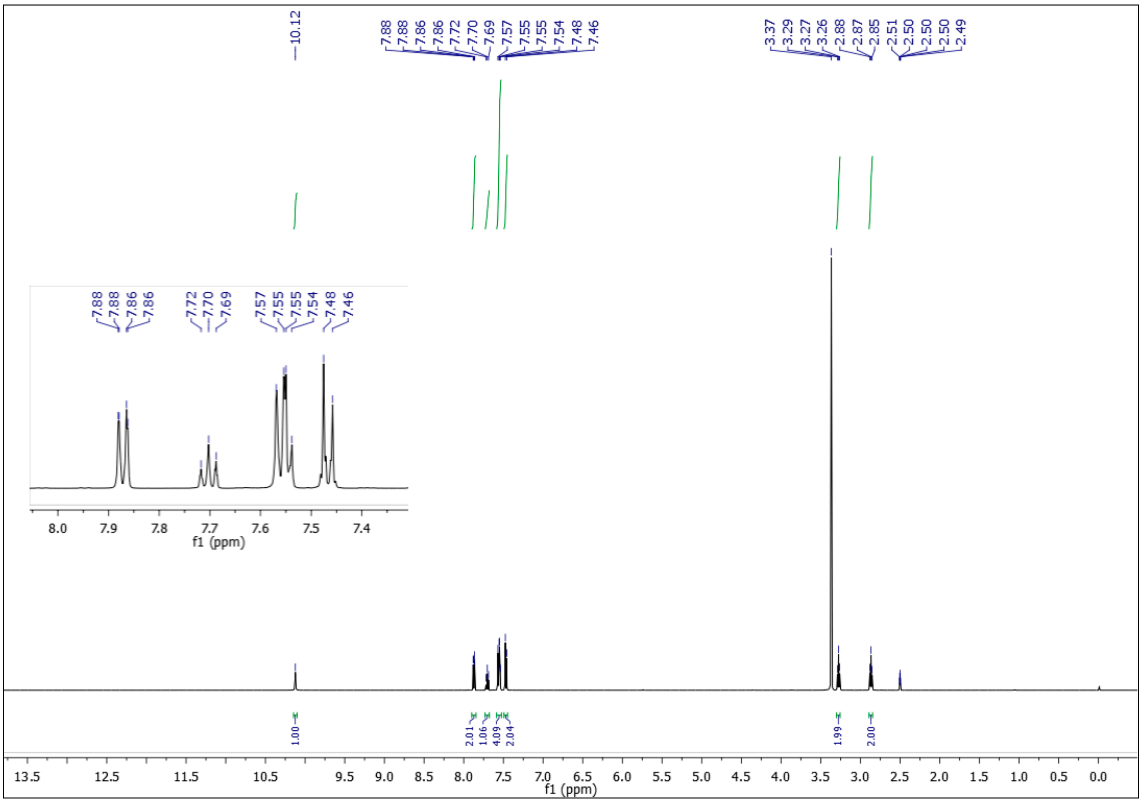

Figure S69. Hydrogen spectrum (500 MHz, DMSO-d6) of N-(4-bromophenyl)benzoselenoethylenelacticamide (NC53).

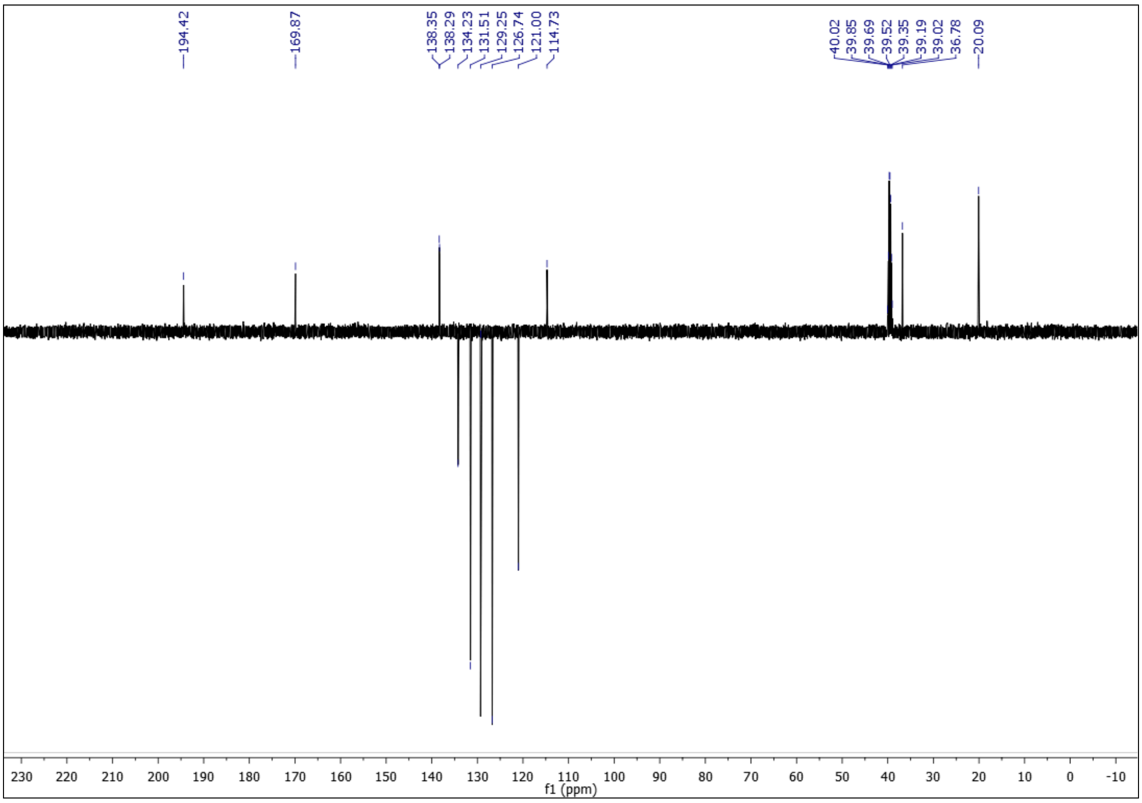

Figure S70. Carbon spectrum (126 MHz, DMSO-d6) of N-(4-bromophenyl)benzoseleneethylenelacticamide (NC53).

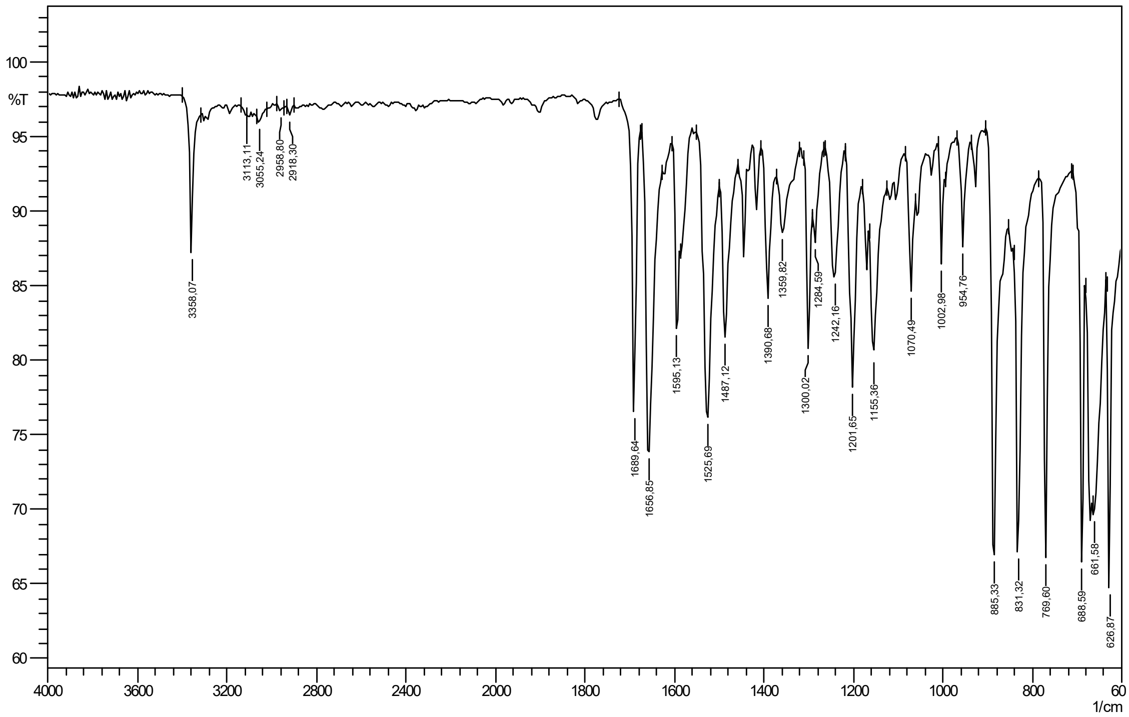

Figure S71. Infrared spectrum (ATR) of N-(4-bromophenyl)benzoselenoethylenelacticamide (NC53).

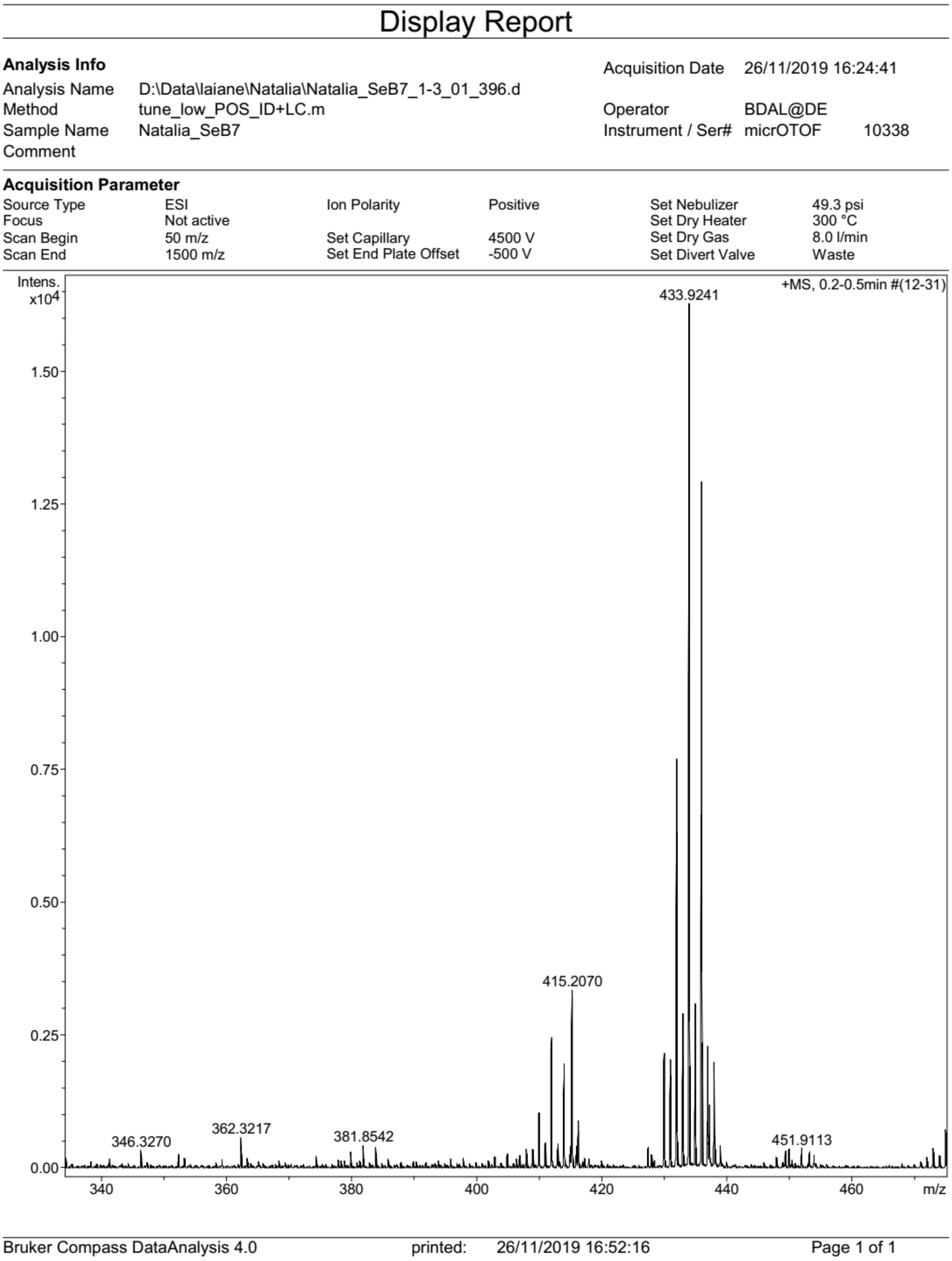

Figure S72. Mass Spectrum of N-(4-bromophenyl)benzoseleneethylenelactamide (NC53).
